# An applied noise model for scintillation-based CCD detectors in transmission electron microscopy

**DOI:** 10.1038/s41598-025-85982-4

**Published:** 2025-01-30

**Authors:** Christian Zietlow, Jörg K. N. Lindner

**Affiliations:** Nanopatterning-Nanoanalysis-Photonic Materials Group, Department of Physics, Paderborn University, Warburgerstr. 100, 33098 Paderborn, Germany

**Keywords:** Noise model, Scintillation detector, Electron microscopy, Non-linearity correction, Brighter-fatter effect, Point spread function, Transmission electron microscopy, Imaging techniques, Transmission electron microscopy, Statistics, Transmission electron microscopy

## Abstract

Measurements in general are limited in accuracy by the presence of noise. This also holds true for highly sophisticated scintillation-based CCD cameras, as they are used in medical applications, astronomy or transmission electron microscopy. Further, signals measured with pixelated detectors are convolved with the inherent detector point spread function. The Poisson noise, arising from the quantized nature of the beam electrons, gets correlated by this convolution, which allows to reconstruct the detector PSF based on the Wiener–Khinchin theorem and the Pearson correlation coefficients under homogeneous illumination conditions. However, correlation also has a strong impact on the noise statistics of basic operations like the binning of signals, as it is usually done in electron energy-loss spectroscopy. Thus, this paper aims to give an insight into the different noise contributions occurring on such detectors, into their underlying statistics and their correlation. Detectors usually suffer from gain non-linearities and quantum efficiency deviations, which must be corrected for optimal results. All these operations influence the noise and are influenced by it, vice versa. In this work, we mathematically describe all these changes and show them experimentally. Methods on how to measure individual noise and correlation parameters are described allowing readers to implement routines for finding them. Sufficient knowledge on the noise of a measurement is not only crucial for classifying its quality and meaningfulness, but also allows for better post-processing operations like deconvolution, which is a common practice in spectroscopy to enhance signals.

## Introduction

Every measurement is subject to noise. The most prominent ones for electron microscopy and especially transmission-electron-microscopy (TEM) certainly are Poisson noise, arising from the quantized signal itself^1,2^, and Gaussian read-out noise from the detector electronics^3,4^. Further, gains that influence the Poisson noise are generated by the parts of the scintillation-based detector^5^ and differences in quantum efficiency between pixels on the detector^6,7^ alter the noise. Correlation effects between pixels caused by the point spread function (PSF) of the detector and mathematical operations in order to correct the signal from detector artifacts further complicate the above mentioned signal alterations. To top it all of, the detector suffers from non-linearities with increasing signal strength.

To an operator, who does not have year-long experience in the subject of noise and statistics, noise statistics in general looks confusing and overwhelming. However, understanding the noise helps to interpret artifacts in the data, helps designing measurement conditions under which certain effects become visible and helps manufacturers to improve their detectors and image acquisition in the first place. Having a valid noise model also allows for modern denoising or deconvolution techniques, which improve the evaluation or make it even possible to evaluate sensitive materials such as organic or biological materials that degrade at very short exposure times in an electron microscope^8–12^. Thus, there is a broad and interdisciplinary need for algorithms in TEM, which require such a noise model. In^13^, we have shown how to design and use ADMM algorithms for denoising and deconvolution in a scientific context, such that they operate unbiased by the user and purely on the measurable noise parameters of a detector and a suitable noise model. Scintillation-based CCD detectors are not limited to electron microscopy, they have use-cases in X-ray detection for astronomy^14^, nuclear physics^15^ as well as in medicine^16–20^. Since the detector architecture uses a CCD, the noise model that we will derive in the following is in large parts valid for general CCD cameras as well.

A ‘rather complex’ statistical framework is needed to describe the noise and all the alterations of measurements on such a detector. Such frameworks can be found in the references^21,22^ and especially in^23^. A typical TEM user, however, may not want to deal with all contributions to the statistics of his detector. Here, we would like to focus on such contributions that are relevant or may appear relevant for the usage of a scintillation-based CCD camera attached to a TEM. Further, a typical TEM user cannot open a CCD detector without the risk of damaging it immediately. This restricts the ability to analyze the detector layers in detail. Thus, many of the parameters such as coupling efficiencies, gains, input and output quanta needed to utilize noise models for cascaded systems from Rabbani^24^ or Cunningham^25^ are not directly accessible. While these are sophisticated statistical descriptions, they cannot easily be applied in practical TEM work.

This paper presents a novel approach to understanding detector noise, one that integrates both theoretical and experimental perspectives to provide a comprehensive framework for noise analysis. Unlike previous studies, which often focus on either theoretical models or experimental measurements, our work seeks to bridge the gap between these two approaches. By identifying the most significant noise contributions relevant to experimentalists, we aim to develop a coherent theoretical framework that can be applied to real-world detectors.

This article starts with a general analysis of key noise sources, such that our approach can be used even with limited statistical expertise. By presenting a comprehensive treatment of the subject, we hope to facilitate a deeper understanding of the overall framework, highlighting the interconnectedness of all components and providing a cohesive framework for noise analysis.

While this paper may be lengthy and detailed, we believe that its comprehensive nature is essential for providing a thorough understanding of detector noise. By integrating theoretical and experimental perspectives, we aim to provide a valuable resource for experimentalists seeking to optimize their detectors and minimize the impact of noise on their measurements.

It is clear that this paper simplifies the vast body of statistical work on noise analysis, which spans thousands of pages of published research. To develop a practical model that can be applied under common conditions, we must identify useful approximations that enable us to isolate and separate the different noise components, allowing us to validate our model through straightforward measurements.

In statistical analysis, the reference frame in which noise is determined is crucial. The measurements proposed in this paper to quantify noise are no exception, and therefore, we strive to provide precise descriptions of our experimental methodology. By doing so, we aim to ensure that our results are reliable and reproducible, and that our model can be applied in real-world scenarios.

**Fig. 1:**
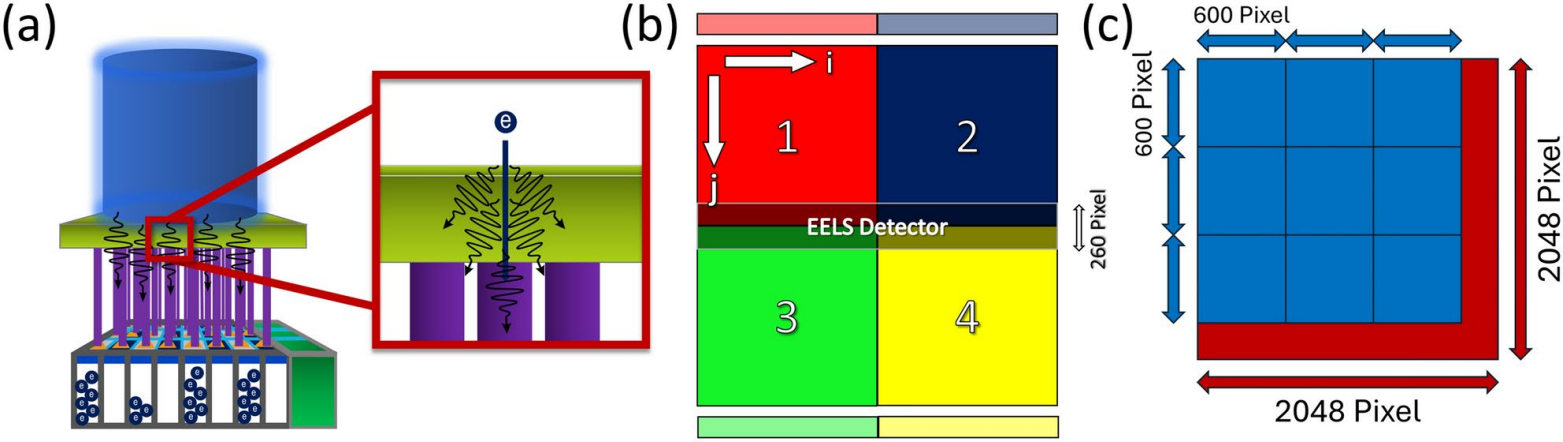
(**a**) Schematic of the scintillation-based detector with the incident electron beam (blue), the fluorescent scintillation layer (green) generating optical photons and the fiber optic (purple) connecting the fluorescence layer with the 2D CCD detector, which reconverts the optical photons in charge carriers and with the analog-digital-converter (dark green) into counts. The red box shall point out, that several photons are generated per incident electron and spread across the fluorescence layer. (**b**) Schematic of the *US1000FT-XP 2* from *Gatan*^26^ with 4-port read-out electronics indicated. The respective ADC ports are shown in faded colors above or below the segments. We define the respective column of the CCD to be indicated by the index *i* and the respective row of the detector by the index *j*. The region for EELS detection is located in the middle of the detector spanning across all four segments with 260 pixels in height. (**c**) Schematic for an exemplary 600 times 600 pixel binning on a 2048 times 2048 pixel detector, where the blue boxes represent the binned pixels and the red regions represent the rest that cannot fully be binned into a 600 times 600 pixel. One can easily see, that for a 2048 times 2048 pixel detector, binning with a number that contains a prime factor unequal to two leads to leftover pixels.

First of all, the general architecture of the detector is needed. In our setup, we use the *US1000FT-XP 2* detector installed in a *Gatan GIF Quantum ER* image filter^26^, which is shown schematically in Fig. 1a. The analysis of this detector is the basis that we will use to evaluate our noise model. The scintillation-based detector mainly consists of three layers: a fluorescence layer, that converts incident electrons into photons; a fiber optic, which guides the photons to a CCD camera; and the CCD camera itself, converting photons into charge carriers. Such systems were statistically analyzed e.g. by Cunningham et al.^25,27^. As shown in Fig. 1b, the CCD camera consists of four segments with respectively 1024 times 1024 pixels, connected to separate read-out ports, where the charge carriers are converted into counts via analogue-digital-converters (ADC). The segmentation increases read-out speeds, but comes at the cost of having different noise properties, which must be regarded individually. In the image filter, the CCD camera is used for both, recording images and spectra. By guiding the electron beam through a series of magnets, the electrons disperse with respect to their kinetic energy allowing to investigate energy losses induced by a specimen. Dispersing the beam in TEM mode is called energy-filtered TEM (EFTEM) and for scanning electron transmission microscopy (STEM) this method is referred to as electron-energy loss spectroscopy (EELS). The features of such loss spectra are of special interest, as they allow to determine e.g. the elemental composition of a given material or to analyze its electronic structure including light-matter interactions. In the middle of the detector, the EELS signal is measured and then summed across all rows *j* to convert a 2D image into a 1D spectrum. We indicated the region on the detector in Fig. 1b, where a typical EELS signal is measured. This measuring process with the CCD introduces a further spreading of the signal, leading to correlations, which was analyzed e.g. by Stierstorfer et al.^28^. Eventually, the summation process can be seen as a post-binning of the image. So, for a full analysis of the noise model useful for both TEM and STEM mode, it is necessary to understand the impact of binning. It is obvious that there are leftover pixels for any binning number containing a prime factor that is unequal to two, considering the analysis of the full 2048 times 2048 pixel detector. A schematic of such a binning is shown as an example in Fig. 1c.

In general, all scintillation-based CCD detectors are quite similar in their design, with the devil being in the ‘details’. There are detectors employing less segments or a lens optics instead of fiber optics. This however does not change the general noise model but only the extend of contributing factors.

In this paper, we focus on the transmission electron microscopy (TEM) mode and give a short outlook on the impact of those findings for STEM-EELS. It is crucial for the measurement of noises to perform measurements without a specimen in the beam path to minimize variations of the signal due to external factors other than noises. This is why we performed all noise measurements in vacuum. The principles of the noise model found here can be applied to any signals acquired from structures of interest.

This paper is organized as follows: following this introduction, “Section Fundamentals of different noise statistics” provides an overview of the statistical formulas and mathematical principles exploited in this paper. This section serves as a reference, to which we will frequently refer during the derivation of our noise model in “Section The noise model”. In this section, we present the proposed noise model, which encompasses the mathematical description of the image acquisition process and the various image corrections required to address deviations and non-linearities in the scintillation-based CCD detector, as well as their influence on the noise model. Additionally, we demonstrate how binning, a crucial step in the formation of EEL spectra, affects correlations and gain in the data. In “Section Evaluation of the noise model”, we describe the procedure for measuring the parameters of the proposed noise model and experimentally validate the mathematical expressions. Moreover, we outline the process for acquiring the necessary detector corrections and present a method for determining the detector PSF. In “Section Theoretical considerations on STEM-EELS measurements”, we examine the implications of our findings for EEL spectra, and “Section Conclusions presents a comprehensive discussion of the overall results and conclusions.

## Fundamentals of different noise statistics

Noise is a stochastic process, allowing for a description in a general statistical framework, even though knowing its exact representation in the data is impossible. This publication aims to provide a comprehensive understanding of the fundamental properties of noise in data generated by scintillation-based CCDs. To establish a solid foundation for our discussion, we briefly outline the underlying mathematical principles in the following sections. In “Sections Gaussian noise” and “Poisson noise”, we introduce the respective probability distributions and examine their behavior under various mathematical operations that are essential for developing the noise model. “Section Noise correlation effects” delves into the impact of correlations on the measured sample standard deviation, a crucial aspect for our analysis. Finally, in “Section Noise and convolution”, we explore the effects of convolutional operations on the noise, providing a thorough understanding of this critical component of the noise model.

### Gaussian noise

The most commonly known statistics describing noise in signal processing certainly is the Gaussian distribution. Coming from statistical independent random processes, it can be described with the probability density function (PDF) $\mathscr {N}$^29^:

1 $$\begin{aligned} \mathscr {N} \! \left[ J\! \left( X\right) = n \, , \, \mu \,,\, \sigma ^{2} \right] = \frac{1}{\sqrt{2\pi \sigma ^{2}}}\cdot \exp \left\{ -\frac{\left( n- \mu \right) ^{2}}{2\sigma ^{2}} \right\} \text { ,} \end{aligned}$$

with the mean value $\mu$ and the variance $\sigma ^{2}$. In general, the normal distribution gives the probability to measure *n* noise counts as a result of a measurement *J* of a random and thus independent variable *X*, which, in our case, might be a pixel number.

To shorten notation, we will use $\mathscr {N} \! \left[ \mu \,,\, \sigma ^{2} \right]$ as a representation of the above described measurement.

Working with Gaussian distributions requires several important mathematical operations, such as addition, subtraction, multiplication and so on. These operations change the mean value and variance to different extents, but fortunately most are commonly found in standard text books of statistics for students. For our work, we need the following operations for our noise model:

Adding two or more independent random variables $X_{w}$ leads to the addition of their mean values and their variances. The resulting distribution also corresponds to a Gaussian distribution^30^. Adding Gaussian distributions is also equal to convolving them^31,32^:

2 $$\begin{aligned} \sum _{w=1}^{W} X_{w} = X_{1} \otimes X_{2} \otimes \cdots \otimes X_{W} = \sum _{w=1}^{W} \mathscr {N}\!\left[ \mu _{w}\, , \,\sigma ^{2}_{w}\right] = \mathscr {N}\!\left[ \sum _{w=1}^{W} \mu _{w}\, , \,\sum _{w=1}^{W} \sigma ^{2}_{w}\right] \text { ,} \end{aligned}$$

where $\otimes$ is the convolution operator.

The multiplication of a Gaussian distributed variable *X* by a constant *c* is given as^29^:

3 $$\begin{aligned} cX = \mathscr {N}\! \left[ c \mu \, ,\,c^{2}\sigma ^{2} \right] \text { ,} \end{aligned}$$

where it is important to note that the factor *c* appears squared in the variance.

The distribution $f_{Z}$ of the product of two uncorrelated random variables $Z=XY$ with the expectation value $E\left( Z\right)$ and variance $\mathop {\textrm{VAR}}\limits \left( Z\right)$ are given as^33^:

4 $$\begin{aligned}\quad\; f_{Z}\left( z\right)&= \int _{-\infty }^{\infty } \frac{1}{|x|}\,f_{X}\left( x\right) \,f_{Y}\left( z/x\right) \, dx\quad \quad\;\text{,}\end{aligned}$$

5 $$\begin{aligned} \quad \;E\left( Z\right)&= E\left( X\right) \cdot E\left( Y\right)\qquad\qquad\qquad\quad\quad\, \text{,} \end{aligned}$$

6 $$\begin{aligned} \mathop {\textrm{VAR}}\limits \left( Z\right)&= \left( \sigma _{X}^{2} +\mu _{X}^{2}\right) \cdot \left( \sigma _{Y}^{2} +\mu _{Y}^{2}\right) - \mu _{X}^{2}\mu _{Y}^{2}\text{ ,} \end{aligned}$$

with the respective probability density functions $f_{X}(x)$ and $f_{Y}(y)$. Here, *x*, *y* and *z* denote the position in the distribution. Note that the resulting distributions are still quite elaborate and often result in infinite sums. Simplifications of these exact representations are a current topic of research^34^.

For the ratio of a random variable $\nicefrac {1}{X}$, one can utilize Díaz-Francés et al.^35^ who showed that the ratio with a Gaussian can be approximated by a normal distribution, if the denominator is closely distributed about its mean value:

7 $$\begin{aligned} \mathscr {N}\!\left[ \mu ,\sigma ^{2}\right] ^{-1}\approx \mathscr {N}\!\left[ \mu ^{-1},\frac{\sigma ^{2}}{\mu ^{4}}\right] \text { .} \end{aligned}$$

### Poisson noise

Another important process occurring as a consequence of the discrete nature of photons or electrons is the Poisson process. Similar to tossing a coin, there are only two possible outcomes: measuring or not measuring an event. This leads to a PDF that is slightly skewed towards higher counts^32,36^:

8 $$\begin{aligned} \mathscr {P}\! \left[ J\!\left( \hat{S}\right) = n \right] = \frac{\hat{S}^{\,n}}{ n!} \exp \left\{ - \hat{S} \right\} \text { ,} \end{aligned}$$

with the probability $\mathscr {P}$ to measure *n* counts of a signal *S* with its expectation value $E\left[ S\right] = \hat{S}$ and *J* again denoting the measurement. We shorten the notation to $\mathscr {P}\! \left[ \hat{S}\right]$. For a sufficiently high count regime, the Poisson distribution converges to the Gaussian distribution, with both the mean value and the variance equal to the expectation value^36^:

9 $$\begin{aligned} \lim _{\hat{S}\rightarrow \infty } \mathscr {P}\! \left[ \hat{S}\right] \approx \mathscr {N} \! \left[ \hat{S} \,,\, \hat{S} \right] \text { .} \end{aligned}$$

In fact, a main characteristic of the Poisson distribution is that the noise variance always equals the expectation value of the signal $\sigma ^{2}=\mu$^36^.

The summation rule for Poisson distributions is given as^31,36^:

10 $$\begin{aligned} \sum _{w} \mathscr {P}\left[ \hat{S}_{w}\right] = \mathscr {P}\left[ \sum _{w} \hat{S}_{w}\right] , \end{aligned}$$

where the sum of Poisson distributions equals the Poisson distribution of the sum of the expectation values. Subtracting Poisson distributions in contrast leads to the Poisson-difference distribution, also known as Skellam distribution^37^:

11 $$\begin{aligned} \mathscr {S}\left[ J\!\left( \hat{S}_{1}\right) -J\!\left( \hat{S}_{2}\right) =n\;,\hat{S}_{1},\hat{S}_{2}\right] = \exp \left\{ -\left( \hat{S}_1+\hat{S}_2\right) \right\} \,\left( \frac{\hat{S}_{1}}{\hat{S}_{2}}\right) ^{\nicefrac {n}{2}} \mathscr {I}_{|n|}\left( 2\sqrt{\hat{S}_{1}\cdot \hat{S}_{2}}\right) \text { ,} \end{aligned}$$

with $\mathscr {I}_{|n|}(x)$ denoting the modified Bessel function of the first kind and $\hat{S}_{1,2}$ the expectation values of $S_{1,2}$. For a shorter notation, we will write $\mathscr {S}\left[ \hat{S}_{1},\hat{S}_{2}\right]$.

Multiplying a Poisson distributed signal $S^{*}$ with a gain factor *g*, such that $S = g\cdot S^{*}$, leads to the distribution^38–40^:

12 $$\begin{aligned} g\cdot \mathscr {P}\! \left[ \hat{S}^{*}\right] = g\cdot \mathscr {P}\! \left[ \frac{\hat{S}}{g}\right] = \mathscr {P}_{g}\! \left[ \hat{S}\right] \quad \text {, with}\quad \mathscr {P}_{g}\! \left[ \hat{S}\right] = \frac{\left( g^{-1}\cdot \hat{S}\right) ^{g^{-1}\cdot n}}{\Gamma \left[ g^{-1}\cdot n +1 \right] } \exp \left\{ -g^{-1}\cdot \hat{S} \right\} \text { ,} \end{aligned}$$

which is denoted as the super-Poisson distribution for $g>1$ and as sub-Poisson distribution for $g<1$^40,41^. Here, the multiplication of the signal is comparable to Eq. 3, as it shifts the mean value of the distribution and changes its variance accordingly. To complete the trio, we will refer to the Poisson distribution as the true-Poisson distribution for the case $g=1$ in the following. This formula is valid for deterministic gains, where each incident electron generates the exact same number of photons. However, this would only allow for *n*-values, which are a multiple of *g*, and integer values of *g*. Usually, this is not the case for scintillation-based detectors, as the generation of photons from beam electrons is a statistical process itself and may vary for each incident electron^25^. Thus, we consider the gain of the detector to be the mean value of said generation process. This definition allows the gain to be a fractional number. As a consequence, in the above formula, we have replaced the factorial by the gamma function $\Gamma \left[ \cdot \right]$ to account for non-integer values within the argument, as $n!=\Gamma \left[ n+1\right]$. It is obvious that the above scaled Poisson distribution is a simple approximation to the true distribution^42^, but we found it to work quite well for us. A description of the true distribution is found in e.g.^25^, but its description is much more complex and requires many input parameters that are usually not directly measurable on a detector, as mentioned in the introduction.

### Noise correlation effects

So far, the above noise distributions assume the noise to be independently distributed. However, in a detector this is rarely the case. Thus we need to explain the impact of correlation on the variance of the noise distributions.

To differentiate the variance measured within a single frame $\sigma _{SF}^{2}$, which is subject to correlation effects, from the variance of a single pixel across many frames $\sigma ^{2}$, which we consider independent and thus ‘true’, we use different variables for them. This differentiation is important as the expected variances of both are not equal under the premise of correlation. In the experimental section of this work, we mainly determine the variances within a single frame $\sigma _{SF}^{2}$ and thus need to elaborate on the implications that this frame of reference offers.

The definition for the variance is given as^30,32^:

13 $$\begin{aligned} \sigma ^{2} = E\left[ S^{2}\right] - E\left[ S\right] ^{2} = E\big [\,S - E\left[ S\right] \,\big ]^{2} \text { ,} \end{aligned}$$

with the expectation value $E\left[ S\right]$ of a random variable *S*, that in our case could be a signal. For a finite number of pixels in a detector and a homogeneous signal, where all expectation values are equal $E\left[ S\right] =E\left[ s_{i,j}\right]$, the true variance is unknown but can be estimated using:

14 $$\begin{aligned} \sigma _{SF}^{2} = E\left[ \,s_{i,j} - \frac{1}{MN}\sum _{m^{*}=1}^{M}\sum _{n^{*}=1}^{N} s_{m^{*},n^{*}}\,\right] ^{2} \text { ,} \end{aligned}$$

with the value of a pixel $s_{i,j}$ and the latter term describing the mean value of all pixels with the double sum across all indices. Here, $i\in \left[ 1,\ldots ,N\right]$ describes the position in the row, and $j\in \left[ 1,\ldots ,M\right]$ describes the position in the column. By replacing $s_{i,j}$ with the difference between pixel and its expectation value $d\!s_{i,j} = s_{i,j} -E\left[ s_{i,j}\right]$ in the above expression, we obtain^43^:

15 $$\begin{aligned} \sigma _{SF}^{2} = E\left[ \,d\!s_{i,j} - \frac{1}{MN}\sum _{m^{*}=1}^{M}\sum _{n^{*}=1}^{N} d\!s_{m^{*},n^{*}} + E\left[ s_{i,j}\right] - \frac{1}{MN}\sum _{m^{*}=1}^{M}\sum _{n^{*}=1}^{N} E\left[ s_{m^{*},n^{*}}\right] \,\right] ^{2} \text { ,} \end{aligned}$$

where the first two terms describe deviations of the variance of a given pixel from the mean variance of the detector. The latter terms vanishes, if the signal is homogeneous, since the expectation value for all *s* is identical in this specific case. Utilizing Eq. 13 leads to^43^:

16 $$\begin{aligned} \sigma _{SF}^{2}&= E\left[ ds_{i,j}^{2} \right] - E \left[ \frac{1}{M \cdot N} \cdot \sum _{m^{*}=1}^{M}\sum _{n^{*}=1}^{N} ds_{m^{*},n^{*}} \right] ^{2}\nonumber \\&= \sigma ^{2} - \frac{1}{\left( M N\right) ^{2}} \cdot \sum _{m^{*}=1}^{M}\sum _{n^{*}=1}^{N}\sum _{p=1}^{M}\sum _{q=1}^{N} E\left[ ds_{m^{*},n^{*}} \cdot ds_{p,q} \right] \text{ ,} \end{aligned}$$

where $E\left[ ds_{i,j}^{2}\right] =\sigma ^{2}$ gives the expectation value of the variance of a given pixel, which is the true uncertainty of the data. The latter term describes the mean covariance of all pixels, with the covariance defined as^32^:

17 $$\begin{aligned} \mathop {\textrm{cov}}\limits \left[ s_{m^{*},n^{*}}\, , \, s_{p,q}\right] = E\Big [\underbrace{\left( s_{m^{*},n^{*}}-E\left[ s_{m^{*},n^{*}}\right] \right) }_{=\, ds_{m^{*},n^{*}}}\cdot \underbrace{\left( s_{p,q}-E\left[ s_{p,q}\right] \right) }_{=\, ds_{p,q}}\Big ] \text { .} \end{aligned}$$

As the noise is homogeneously distributed across the detector, the covariance $\mathop {\textrm{cov}}\limits \left[ s_{m^{*},n^{*}}, \, s_{p,q}\right]$ only depends on the horizontal and vertical separation $m= p-m^{*}$ and $n= q-n^{*}$, respectively, and not on the individual pixel $\text {s}_{i,j}$. Often, *m* and *n* are referred to as horizontal and vertical lag. We can thus simplify:

18 $$\begin{aligned} \mathop {\textrm{cov}}\limits \left[ s_{m^{*},n^{*}}, \, s_{p,q}\right]&= \mathop {\textrm{cov}}\limits \left[ s_{m^{*},n^{*}}, \, s_{m^{*},n^{*}}\right] \cdot \rho _{m,\,n} \qquad \text {, with} \qquad \rho _{m,\, n}=\frac{\mathop {\textrm{cov}}\limits \left[ s_{m^{*},n^{*}}, \, s_{m^{*}+m,\,n^{*}+n}\right] }{\mathop {\textrm{cov}}\limits \left[ s_{m^{*},n^{*}}, \, s_{m^{*},n^{*}}\right] }\nonumber \\&=\sigma ^{2}\cdot \rho _{m,\,n}\qquad\qquad\qquad\qquad\qquad\qquad\qquad\quad\, =\frac{\mathop {\textrm{cov}}\limits \left[ s_{m^{*},n^{*}}, \, s_{m^{*}+m,\,n^{*}+n}\right] }{\sigma ^{2}}\text{ ,} \end{aligned}$$

where $\rho _{m,n}$ are the Pearson correlation coefficients^44^, describing the autocovariance function of two pixels in the data set. In order to calculate the variance within the single frame, we need to combine Eq. 16 with Eq. 18. However, two double sums are hard to handle and thus we need a simpler solution. For the values $n^{*}=1$ and $q=1$ (thus $n=0$) within the total sum, we obtain:

19 $$\begin{aligned} \sum _{m^{*}=1}^{M}\sum _{p=1}^{M} E\left[ ds_{m^{*},1} \cdot ds_{p,1} \right] = \sigma ^{2}\cdot \sum _{m^{*}=1}^{M} \rho _{1-m^{*},0} + \rho _{2-m^{*},0} + \cdots + \rho _{M-m^{*},0} = \sum _{m=-\left( M-1\right) }^{M-1} \left( M-\left| m\right| \right) \cdot \rho _{m,0} \text { .} \end{aligned}$$

It can easily bee seen that by increasing $m^{*}$ from 1 to *M*, $\rho _{0,0}$ is contained *M* times within the sum. We further find $\rho _{1,0}$ and $\rho _{-1,0}$ to be contained $(M-1)$ times, $\rho _{2,0}$ and $\rho _{-2,0}$ $(M-2)$ times and so on, until we obtain $\rho _{M-1,0}$ and $\rho _{-\left( M-1\right) ,0}$ only once. Repeating this pattern for all the other values of $n^{*}$ and *q* to complete the two double sums in Eq. 16, leads to:

20 $$\begin{aligned} \sigma _{SF}^{2} = \sigma ^{2}\cdot \left( 1 - \frac{1}{\left( M\cdot N\right) ^{2}} \sum _{m=-\left( M-1\right) }^{M-1}\sum _{n=-\left( N-1\right) }^{N-1} \left( M-\left| m\right| \right) \cdot \left( N-\left| n\right| \right) \cdot \rho _{m,\,n}\right) \text { ,} \end{aligned}$$

where $\rho _{m,\,n} \in \left[ -1,1\right]$ ranges from -1, in case of total anti-correlation, through 0, for uncorrelated data, to 1, for total correlation of the data^32^. Naturally, a given pixel is always totally correlated with itself, so $\rho _{0,0} = 1$. In a case with uncorrelated noise between pixels, all other coefficients are zero $\rho _{m,\,n} =0$. By simplifying and rearranging the sample variance is revealed^45^:

21 $$\begin{aligned} \sigma ^{2}&= \frac{MN}{M\cdot N -1} \cdot \sigma _{SF}^{2}\nonumber \\&=\frac{1}{M\cdot N -1}\cdot \sum _{i=1}^{M}\sum _{j=1}^{N} E\left[ \,s_{i,j} - \frac{1}{MN}\sum _{m^{*}=1}^{M}\sum _{n^{*}=1}^{N} s_{m^{*},n^{*}}\,\right] ^{2}\text { ,} \end{aligned}$$

by utilizing Eq. 14. In a case of total anti-correlation, where all other coefficients $\rho _{m,\,n} = -1$, the single frame variance $\sigma _{SF}^{2} \rightarrow 2\sigma ^{2}$ approaches twice the true variance, as the number of pixels increases. Conversely, in case of total correlation of the data, with all $\rho _{m,\,n} = 1$, Eq. 20 equals zero as a logical consequence. Per definition all pixels must have the same value in this case. We can thus establish, that correlated data exhibits a smaller variance than uncorrelated data and that anti-correlation leads to higher measured sample variances.

Considering all of the above, we can define a factor $\beta _{corr}$ that accounts for the change in the sample variance due to correlation:

22 $$\begin{aligned} \beta _{corr} = \frac{MN}{MN-1}\cdot \left( 1 - \frac{1}{\left( M\cdot N\right) ^{2}} \sum _{m=-\left( M-1\right) }^{M-1}\sum _{n=-\left( N-1\right) }^{N-1} \left( M-\left| m\right| \right) \cdot \left( N-\left| n\right| \right) \cdot \rho _{m,n}\right) \text { ,} \end{aligned}$$

with $\beta _{corr} \in \left[ 0,\,2\right]$. It can easily be seen that for large sets of pixels $\beta _{corr} \rightarrow 1$ and thus the influence of correlation decreases. With the newly defined $\beta _{corr}$, we can rewrite Eq. 20 to:

23 $$\begin{aligned} \beta _{corr} \cdot \sigma ^{2} = \frac{1}{M\cdot N -1} \sum _{i=1}^{M}\sum _{j=1}^{N} E\left[ \,s_{i,j} - \frac{1}{MN}\sum _{m^{*}=1}^{M}\sum _{n^{*}=1}^{N} s_{m^{*},n^{*}}\,\right] ^{2}\text { .} \end{aligned}$$

where it can be seen that the measured variance within a single frame changes with correlation.

Correlation indicates a common process that links both variables. It thus does not influence the shape of a Gaussian or a Poisson distribution, except for their variance:

24 $$\begin{aligned} \mathscr {N}\!\left[ \mu \, , \,\sigma ^{2}\right] {\mathop {\longrightarrow }\limits ^{Correl.}} \mathscr {N}\!\left[ \mu \, , \,\beta _{corr} \cdot \sigma ^{2}\right] \quad \text {and} \quad \mathscr {P}\left[ \hat{S}\right] {\mathop {\longrightarrow }\limits ^{Correl.}} \mathscr {P}_{\beta _{corr}}\left[ \hat{S}\right] \text { .} \end{aligned}$$

For the true-Poisson distribution this necessarily leads to a sub-Poisson distribution for correlation or a super-Poisson in case of anti-correlation. This means that correlation of the signal acts like an additional gain-factor on the Poisson distribution, when considering the noise inside an image. In contrast, the statistics of a single pixel in a series of measurements is not altered. This is for instance the case for EELS-mapping, where a series of spectra is taken on the same detector.

**Fig. 2:**
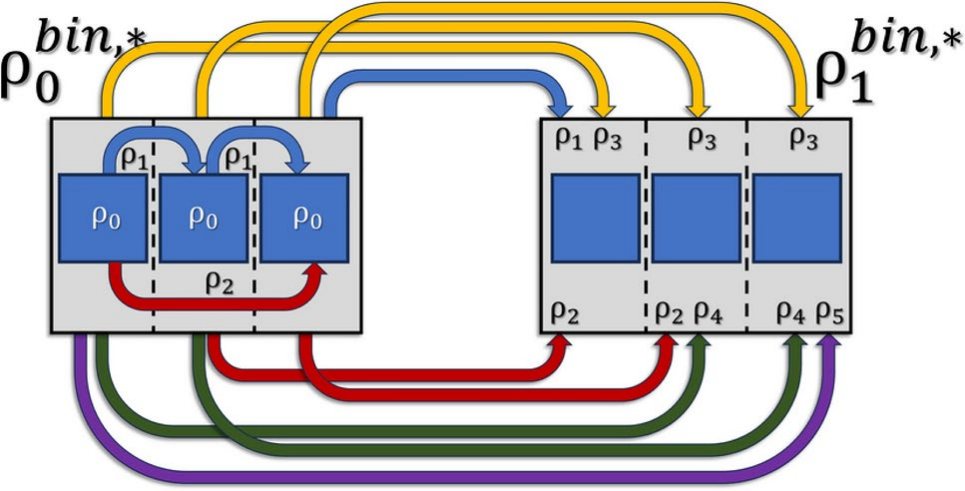
This schematic shows six pixels (blue) in a row, out of which the first three are binned into one pixel (grey). The same is done for the next three pixels. The indices of the Pearson correlation coefficient denote the distance between correlated pixels, thus one can easily count the amount of possible distances within a new or between new, added up pixels. By adding three neighboring pixels (blue) on the left side, one can see that the $\rho _{0}^{bin,*}$-coefficient of the new pixel (grey) must incorporate 3 times the $\rho _{0}$-coefficient, 2 times the $\rho _{1}$-coefficient, and once the $\rho _{3}$-coefficient. The correlations, expressed by their Pearson correlation coefficients, are indicated with a blue arrow for next neighbors, with a red arrow for the second next neighbors as well as yellow, green and purple for the more distant neighbors. Note that the distance between pixels works in both ways, so the negatively numbered Pearson correlation coefficients $\rho _{-1}$ and $\rho _{-2}$ must be added to the respective new coefficient in equal number as their positive counterparts. $\rho _{0}$, indicated in white, is the correlation of a pixel with itself and is thus defined as 1. Now, the correlation between the binned pixels is of interest. On the right side the new $\rho _{1}^{bin,*}$-coefficient must inherit 3 times the $\rho _{3}$-coefficient, 2 times the $\rho _{2}$-coefficient, and once the $\rho _{1}$-coefficient of the unbinned pixels. Additionally, one obtains 2 times the $\rho _{4}$-coefficient and once the $\rho _{5}$-coefficient. So, when binning *w* pixels, one sees that this requires $2w-1$ additions of the Pearson correlation coefficients of the former system. These principles extend on higher and on negative new coefficients. Now, $\rho _{0}^{bin,*}$ gives the multiplicand for the old true variance $\sigma ^{2}$ to form the new $\sigma _{true,bin}^{2}$. Normalizing all new coefficients by $\rho _{0}^{bin,*}$ gives the nth Pearson correlation coefficient $\rho _{n}^{bin}$ of the new system, with which a new $\beta _{corr}^{bin}$ can be calculated using Eq. 22.

Correlation also drastically changes the addition of correlated random variables. Adding pixels of a detector, e.g. by binning, leads to a reduction of the pixel-set, but also to additions within the Pearson correlation coefficients as shown schematically in Fig. 2. Under the influence of correlation, Eq. 2 changes into^32^:

25 $$\begin{aligned} \sum _{w=1}^{W} \mathscr {N}\!\left[ \mu _{w}\, , \,\sigma ^{2}_{w}\right] = \mathscr {N}\!\left[ \sum _{w=1}^{W} \mu _{w}\, , \, \sum _{w=1}^{W} \sum _{w^{*}=1}^{W} \mathop {\textrm{cov}}\limits \left[ S_{w},S_{w^{*}}\right] \right] \text { ,} \end{aligned}$$

in the general case, where the covariances between variables add to the total variance. Under the assumption that all pixels have the same variance and the same expectation value, we can state for a detector:

26 $$\begin{aligned} \sum _{h=1}^{H}\sum _{v=1}^{V} \mathscr {N}\!\left[ \mu _{h,v}, \,\sigma ^{2}_{h,v}\right]&= \mathscr {N}\!\left[ \sum _{h=1}^{H}\sum _{v=1}^{V} \mu _{h,v}, \,\sigma ^{2} \cdot \rho _{0,0}^{bin,*} \right]\qquad\qquad\quad \;\;\text { , with} \nonumber\\  \rho _{0,0}^{bin,*}&= \sum _{h=-\left( H-1\right) }^{H-1}\sum _{v=-\left( V-1\right) }^{\left( V-1\right) } \left( H-\left| h\right| \right) \left( V-\left| v\right| \right) \cdot \rho _{h,v} \text { ,} \end{aligned}$$

with the summation being limited by the number of available pixels $H \in \left[ 1,M\right]$ and $V \in \left[ 1,N\right]$. In general, the new Pearson coefficients $\rho _{m,n}^{bin}$ of a system after vertical summation of *V* rows and horizontal summation of *H* columns are given as:

27 $$\begin{aligned} \rho _{m,n}^{bin} = \frac{\sum _{h=-\left( H-1\right) }^{H-1}\sum _{v=-\left( V-1\right) }^{V-1} \left( H-\left| h\right| \right) \left( V-\left| v\right| \right) \cdot \rho _{H\cdot m + h,V\cdot n + v}}{\rho _{0,0}^{bin,*}} \text { ,} \end{aligned}$$

where the central Pearson correlation coefficient is always defined as $\rho _{0,0}^{bin}=1$.

The Wiener–Khinchin theorem^46,47^ states that the autocovariance function *K* of a random process and the power spectral density (PSD) form a Fourier-transform pair, which allows to determine all the Pearson coefficients $\rho _{n,m}$ of an image $\xi$ by normalizing with respect to the maximum entry:

28 $$\begin{aligned} \rho \left( \xi \right) = \frac{K\left( \xi \right) }{\sigma ^{2}} = \frac{\mathscr {F}^{-1}\left[ PSD\left( \xi \right) \right] }{\mathop {\textrm{max}}\limits \left\{ \mathscr {F}^{-1}\left[ PSD\left( \xi \right) \right] \right\} } \qquad \text {, with} \qquad PSD\left( \xi \right) = \frac{\left| \, \mathscr {F}\left[ \xi - \frac{1}{N\cdot M} \sum _{i=1}^{M}\sum _{j=1}^{N}\xi _{i,j}\right] \, \right| ^{2}}{N\cdot M} \text { ,} \end{aligned}$$

where $\mathscr {F}\left[ \cdot \right]$ and $\mathscr {F}^{-1}\left[ \cdot \right]$ denote the 2D Fourier transform and its inverse, while $\left| \cdot \right|$ denotes the absolute value. Autocovariance and autocorrelation are two terms that are often used synonymously^48^. However, the autocovariance function $K\!\left( \xi \right) _{x,y,x',y'}$ between the positions $\left( x,y\right)$ and $\left( x',y'\right)$ equals the autocorrelation function $R_{x,y,x',y'}\!\left( \xi \right)$ with the mean values of both positions multiplied and subtracted^49^:

29 $$\begin{aligned} K_{x,y,x',y'}\!\left( \xi \right) = R_{x,y,x',y'}\!\left( \xi \right) -\mu _{x,y}\!\left( \xi \right) \mu _{x',y'}\!\left( \xi \right) \text { .} \end{aligned}$$

### Noise and convolution

So far, we have described the fundamentals of different noise distributions and how performing mathematical operations with them changes their respective distributions. We further discussed, how correlation changes the measured variances within a single frame and the resulting variances, when adding variables. What we have not discussed yet, is how convolution changes the noise on a detector.

From a mathematical perspective, convolution and cross-correlation are closely related, with the only difference being the direction with which the kernel is applied^50^. Revisiting the last section, autocorrelation is nothing else than cross-correlation between a signal and itself^50,51^. In discrete form, convolution and cross-correlation are given as^51^:

30 $$\begin{aligned} \left[ f \otimes g\right] _{m,n} = \sum _{m^{*}=-\infty }^{\infty }\sum _{n^{*}=-\infty }^{\infty } f_{m-m^{*},\,n-n^{*}}\,g_{m^{*},\,n^{*}} \qquad \text {and}\qquad \left[ f \star g\right] _{m,n} = \sum _{m^{*}=-\infty }^{\infty }\sum _{n^{*}=-\infty }^{\infty } \overline{f_{m^{*}-m\,,\,n^{*}-n}}\,g_{m^{*},\,n^{*}} \text { ,} \end{aligned}$$

where $\overline{f_{m^{*}-m,\,n^{*}-n}}$ denotes the complex conjugate of $f_{m^{*}-m,\,n^{*}-n}$. If f and g are Hermitian, both convolution and cross-correlation are equal $\left[ f \otimes g\right] _{m,n} =\left[ f \star g\right] _{m,n}$^51,52^. Knowing this and considering the last section, where we discussed the Pearson correlation coefficients, the idea is obvious that correlation and convolution are closely related. And indeed, one can show how convolutions changes the power spectral density of the noise, which is greatly explained in i.e. reference^21^. In this section, we will elaborate on this and try to summarize the most important points.

Since the convolution process satisfies the distributive property^50^, we can separate a given signal *S* into a pure-signal $\hat{S}$ and a pure-noise component. Approximating the Poisson distribution by a Gaussian $\mathscr {N}\!\left[ 0,\sigma _{S}^{2}\right]$ and convolving, leads to both the signal and the noise being convolved with the same kernel $\Omega ^{*}$:

31 $$\begin{aligned} \Omega ^{*}\otimes (\hat{S}+\mathscr {N}\!\left[ 0,\sigma _{S}^{2}\right] )= \Omega ^{*} \otimes \hat{S} + \Omega ^{*} \otimes \mathscr {N}\!\left[ 0,\sigma _{S}^{2}\right] \text { .} \end{aligned}$$

From this point on, we focus on the noisy part of the equation and set aside the convolved signal. Considering a homogeneous signal, the Poisson noise $\sigma _{S}$ is evenly distributed. We can split the convolution into a gain *g* and a normalized kernel $\Omega$, which we apply on the noise. For noise smoothing to occur by convolution, a scattering process is needed that generates multiple particles per incident electron, with $g \gg 1$, enabling them to distribute laterally while remaining correlated due to their common origin. This criterion is more than fulfilled for the TEM, as every incident beam electron creates a cloud of hundreds to thousands of photons in the scintillation layer.

In contrast, a smoothing of the noise would not be possible if only one photon was created^24,25^. In this case, some lateral deviation from the designated path would indeed lead to image blurring, but not affect the noise at all. Further, Cunningham et al.^25^ pointed out that the conversion gain from electrons to photons is a statistical process itself and thus subject to variations, which they described as Poisson distributed. For lower gains, Eq. 12 must be altered by an noise excess term^25^ to account for the correct noise variance. Thus, we need to consider a high conversion gain such that the overall influence of these deviations is small for a sufficient approximation.

Given every pixel of this image is a representation drawn from the noise distribution $\zeta _{i,j} \in \mathscr {N}\!\left[ 0,\sigma _{S}^{2}\right]$, we can write:

32 $$\begin{aligned} \Omega ^{*} \otimes \zeta = g\cdot \Omega \otimes \zeta \qquad \text {, with}\qquad g = \sum _{m,n}^{M,N}\Omega _{m,n}^{*} \quad \text {and}\quad \Omega =\frac{\Omega ^{*}}{g} \text { .} \end{aligned}$$

Autocorrelation yields the Pearson coefficients, as described in the previous section, multiplied by the variance. It is defined as the cross-correlation of a signal with itself. So, the autocorrelation function of convolved noise is given as:

33 $$\begin{aligned} \Big [g\cdot \Omega \otimes \zeta \star g\cdot \Omega \otimes \zeta \Big ]_{m,n} = g^{2}\cdot \Big [\Omega \star \Omega \otimes \zeta \star \zeta \Big ]_{m,n} \text { ,} \end{aligned}$$

where we use the associative property for a scalar multiplication^50^ and Eq. 3 for the gain *g*. Further, we can rearrange the equation, in case both $\Omega$ and the distribution of noise in the image are Hermitian, due to the associative property of the convolution^50^. Scattering processes are of symmetrical nature and the detector inherits a x-/y- symmetry of the detector-pixels, thus we can assume that $\Omega$ is in close approximation to being symmetric with respect to its main diagonal. Additionally, we can assume that all elements $\Omega _{m,n} \in \mathbb {R}$, which then satisfies a Hermitian matrix.

In case the noise is uncorrelated, the autocovariance function yields the Pearson correlation coefficients multiplied by the variance of the image $\sigma _{S}^{2}$. The Pearson correlation coefficients can be written as a Dirac delta functional $\rho _{m,n} = \delta _{m,n}$, as only the $\rho _{0,0}=1$. The only functional *H* obeying $\left[ H \star H\right] _{m,n} = \delta _{m,n}$ is again a Dirac delta functional, which also satisfies a Hermitian matrix:

34 $$\begin{aligned} g^{2}\cdot \Big [\Omega \star \Omega \otimes \zeta \star \zeta \Big ]_{m,n} = g^{2}\cdot \left[ \Omega \star \Omega \otimes \delta \cdot \sigma _{S}^{2}\right] _{m,n}= g^{2}\sigma _{S}^{2}\cdot \left[ \Omega \star \Omega \right] _{m,n} \text { ,} \end{aligned}$$

where the convolution with $\delta$ gives the original expression. The equation can be rewritten:

35 $$\begin{aligned} g^{2}\sigma _{S}^{2}\cdot \left[ \Omega \star \Omega \right] _{m,n} = g^{2}\sigma _{S}^{2}\cdot \left[ \Omega \otimes \Omega \right] _{m,n} \text { .} \end{aligned}$$

We see, that the autocorrelation takes the shape of the convolution kernel convolved with itself. Considering a process with a sufficiently high gain, we see that convolving noise with a kernel broader than a Dirac delta peak $\delta$ leads to a reduction of the central element $\left[ \Omega \otimes \Omega \right] _{0,0}\le 1$. Since autocorrelation gives the Pearson coefficients and their central element is defined as $\rho _{0,0}=1$, we need to rescale it to 1:

36 $$\begin{aligned} g^{2}\sigma _{S}^{2}\cdot \left[ \Omega \otimes \Omega \right] _{0,0}\frac{\left[ \Omega \otimes \Omega \right] _{m,n}}{\left[ \Omega \otimes \Omega \right] _{0,0}} = g^{2}\cdot \sigma _{S}^{2}\cdot \beta _{conv}\cdot \rho _{m,n} \quad \text {, with} \quad \rho _{m,n} =\frac{\left[ \Omega \otimes \Omega \right] _{m,n}}{\left[ \Omega \otimes \Omega \right] _{0,0}} \text { ,} \end{aligned}$$

by introducing a smoothing factor $\beta _{conv}$ for the correlation, given as:

37 $$\begin{aligned} \beta _{conv} =\left[ \Omega \otimes \Omega \right] _{0,0} = \left( \sum _{m=-\left( M-1\right) }^{M-1}\sum _{n=-\left( N-1\right) }^{N-1} \frac{\left[ \Omega \otimes \Omega \right] _{m,n}}{\left[ \Omega \otimes \Omega \right] _{0,0}}\right) ^{-1} =\left( \sum _{m=-\left( M-1\right) }^{M-1}\sum _{n=-\left( N-1\right) }^{N-1} \rho _{m,n}\right) ^{-1}\text { ,} \end{aligned}$$

where $\beta _{conv} \in \left[ 0,1\right]$. We obtain a new variance $\sigma _{\Omega \, \otimes \, S}^{2} = \beta _{conv}\cdot \sigma _{S}^{2}$ for the convolved signal, which is reduced compared to the original.

Again, regarding Eq. 33 for the case the noise is somehow correlated, we obtain the Pearson correlation coefficients to be Hermitian as a result of the autocorrelation function^51^. By utilizing the associative property of the convolution^50^, the equation can be rewritten as:

38 $$\begin{aligned} \sigma _{S}^{2}\cdot \rho _{m,n}= \sigma _{S}^{2} \cdot \left[ \Omega _{1}\otimes \Omega _{0}\otimes \delta \star \Omega _{1}\otimes \Omega _{0}\otimes \delta \right] _{m,n} = \sigma _{S}^{2}\cdot \left[ \Omega \otimes \Omega \right] _{m,n} \quad \text {, with}\qquad \Omega =\Omega _{0}\otimes \Omega _{1} \text { .} \end{aligned}$$

We have shown that the convolution of a signal *S* with a kernel $\Omega$ leads to a gain *g*, a smoothing factor $\beta _{conv}$ reducing the variance of the signal $\sigma _{S}$, and to correlation, which further smoothens the variance with a factor $\beta _{corr}$, if measured within the same image (see Eq. 22). In Eq. 31, we approximated the Poisson distribution by a Gaussian to separate noise and signal. We need to reassemble both again in order to determine the effect of convolution on the Poisson distribution. Considering that the noise inherently follows the signal, due to the quantized nature of the electron, and a gain combined with a convolution changes the expected variance, we can state that convolution leads to a super- or sub-Poisson distribution, depending on the gain and the smoothing factor. Again, for a Poisson distributed signal $\hat{S}^{*}$ and an unnormalized convolution kernel $\Omega ^{*} = g\cdot \Omega$, with $S=g\cdot S^{*}$, we obtain:

39 $$\begin{aligned} \Omega ^{*} \otimes \mathscr {P}\left[ \hat{S}^{*}\right] = \Omega \otimes \mathscr {P}_{g}\left[ \hat{S}\right] = \mathscr {P}_{g\cdot \beta _{conv}\cdot \beta _{corr}}\left[ \Omega \otimes \hat{S}\right] \quad \text {, given that} \quad g \gg 1 \text{ ,} \end{aligned}$$

as multiple particles are needed as a result of a scattering event to spread out.

Owing to the similarity between correlation and convolution, one can determine the PSF, or in other words the convolution kernel, of the entire detector with all its complex architecture as the inverse Fourier transform of the square root of the PSD function Eq. 28:

40 $$\begin{aligned} \Omega = \mathscr {F}^{-1}\left[ \Big (\mathscr {F}\left[ \rho \left( \xi \right) \right] \Big )^{\nicefrac {1}{2}}\right] \text { .} \end{aligned}$$

So, if the signal convolved with the detector PSF $\Omega \otimes \hat{S}$ is sufficiently known, e.g. homogeneously distributed, the mean value of every pixel can be subtracted from a noisy image, following Eq. 28, and the detector PSF can easily be found hidden within the noise. Later in this paper, we will show how this is done under experimental conditions.

However, summing the Pearson coefficients of a stationary process $\rho ^{*}$, such as found on a CCD, eventually yields zero^53^:

41 $$\begin{aligned} \sum _{m=-\left( M-1\right) }^{M-1}\sum _{n=-\left( N-1\right) }^{N-1} \rho ^{*}_{m,n} = 0\text { .} \end{aligned}$$

Unfortunately, this leads to an underestimation of the higher coefficients and even produces negative coefficients, such that the above equation is satisfied.

Now, that we have outlined the mathematical and statistical principles, we can apply them to a real detector to develop a valid noise model.

## The noise model

As is shown in the following, the acquisition of images with a scintillation-based CCD detector like the *US1000FT-XP 2* detector employed in the *‘Gatan Image Filter (GIF) Quantum ER’* is a rather complex process. In these detectors, several transformation processes, from electrons to photons to excitons to counts, are necessary to gather an image. Starting with a general description of different types of noises and their origins, the following section will guide through the acquisition process of the camera itself and the mathematical description of the noises connected to it. Usually these images are further processed to improve the quality of data. Gain normalization and dark frame subtractions are standard procedures to clear the images of detector induced artifacts. Pixel binning and summations of the 2D images to individual spectra, like in EELS, are done to improve the visual understanding of the data, to reduce storage space and processing time - but all these procedures change the noise level and the corresponding noise distribution. To describe all these processes mathematically, we need a lot of different variables. We found it helpful to have a list, where all following variables are explained briefly, to increase the readability of the paper. This list can be found in the supplementary information.

**Fig. 3:**
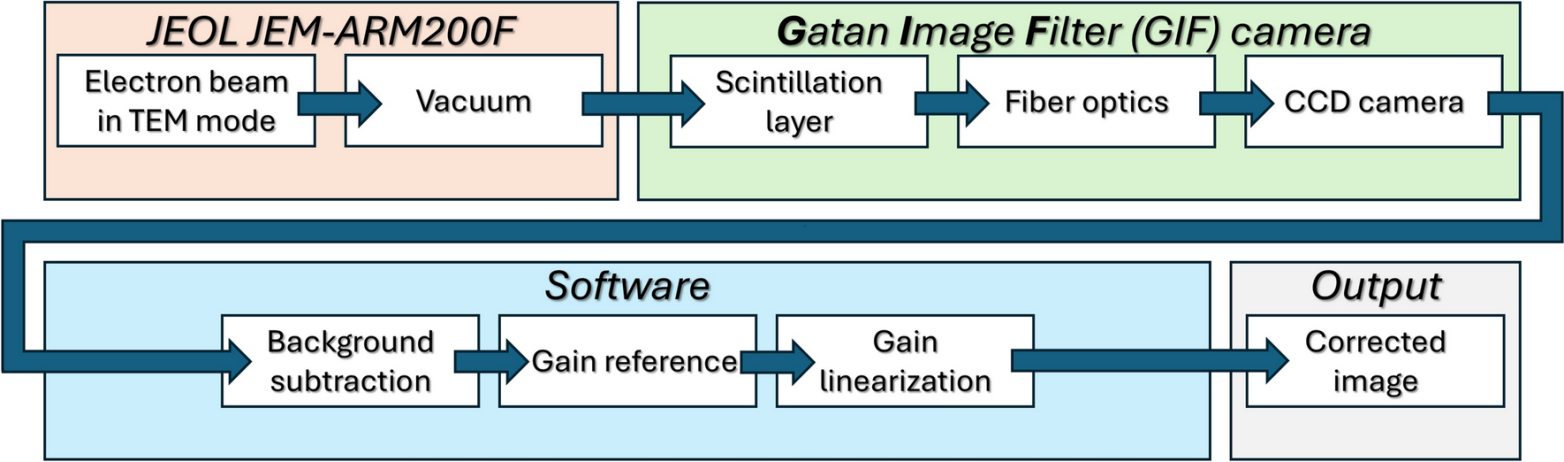
Schematic flow chart of the image acquisition process. Our experimental setup utilizes a *‘JEOL JEM-ARM200F’* transmission electron microscope with no specimen within the beam path, since we are only interested in the image noises. The microscope constitutes the initial stage of the image acquisition process (depicted in orange). The image acquisition process then splits into two primary components, the detector hardware, comprising the *‘Gatan Image Filter (GIF) Quantum ER’* camera, which generates the raw image data (indicated in green); and the subsequent software post-processing (depicted in blue). The combination of hardware and software components ultimately yields the corrected image as output (depicted in gray). The hardware component can be further divided into three distinct detector layers: First, the scintillation layer, where incident beam electrons are converted into photons. Second, the fiber optics system, which guides these photons to the subsequent layer. Third, the CCD camera, where the photons are converted into excitons and ultimately into counts. Following the hardware processing, the software post-processing corrects for offsets in the image by subtracting a background frame. To compensate for quantum deviations, the resulting image is then multiplied by a gain reference acquired under homogeneous illumination conditions prior to the experiment. Finally, the counts are corrected via gain linearization, which accounts for the non-linear behavior of the CCD detector with increasing count numbers.

To facilitate understanding of the noise model, we provide a flow chart of the image acquisition process in Fig. 3, serving as a guide for the reader throughout this paper.

Our experimental setup consists of a *‘JEOL JEM-ARM200F’* microscope, with no specimen in the beam path, as we focus on noise analysis. The beam is directed into the GIF camera, where it interacts with a scintillation layer, converting the incident electrons into photons. These photons are subsequently transmitted through fiber optics to a CCD camera. In “Section Signal and detector noise”, we discuss beam correlations and demonstrate how convolution with the detector’s point-spread function (PSF) affects the resulting Pearson correlation coefficients of this correlation phenomenon. We also introduce multiple gain factors associated with individual detector layers and the corresponding smoothing factors arising from convolution operations. A simple mathematical framework is provided to describe detector deviations and non-linearities, which require corrections. Furthermore, we identify and describe various components of detector noise.

Subsequently, the detector characteristics are removed through multiple post-processing steps, which are implemented in software and affect both signal and detector noises. We begin by examining the impact of background subtraction on detector noise in “Section Dark frame subtraction”. Next, we describe the formation of a gain reference image in “Section The acquisition of a gain reference”, which is crucial for determining the uncertainty associated with such a measurement. In “Section The application of a gain reference”, we discuss how the application of the gain reference alters both signal and detector noises. Furthermore, we address the non-linearity effects of the camera, which must be incorporated into the noise model. In “Section Gain non-linearities”, we explain how to include these corrections and demonstrate their impact on signal and detector noises. Notably, the non-linearity correction must be applied not only to the measurement data but also to the gain reference, as it is also acquired using a non-linear detector. Additionally, we discuss the brighter-fatter effect, a phenomenon that alters the width of the detector’s point-spread function (PSF) with increasing signal strength, driven by charge diffusion processes in the CCD camera. This effect is considered essential in our noise model.

As binning of multiple detector pixels is a commonly employed feature, we examine its impact on correlation, detector PSF, signal, and detector noises in “Section Binning of the detector”.

A comprehensive understanding of the individual noise processes is essential for describing the entire image formation process. Therefore, we will provide a detailed outline of the complete noise model in the subsequent sections, following the framework described above. This thorough explanation will facilitate a deeper understanding of the complex interactions between various noise components and their effects on the imaging process.

### Signal and detector noise

Due to the quantization of electrons, the probability of measuring an electron leaving the electron gun in a given time interval can be modeled as a pure-Poisson distribution $\mathscr {P}\! \left[ \hat{S}_{src,el}\right]$ (see Eq. 8). However, during the acceleration phase, Coulomb forces between beam electrons cause spacio-temporal correlations in the electron beam^54,55^, such that the electron beam is correlated within itself to some degree. This depends on beam currents and the correlation time, within which consecutively emitted electrons are correlated. Indeed, these beam correlations are quite important for the measurements and necessary to regard, as will be shown later in this paper. By broadening the beam in TEM mode, the electrons from the electron gun, which can be modeled as a point-source, are deflected with a kernel $\Omega _{TEM}^{*}$ to form a disc. It is thus symmetric, which allows us to interchangeably use convolution and autocorrelation. Since the uncorrelated electrons are independent, the Poisson statistics is not affected by the deflection, which is represented by the convolution being applied inside the Poisson term $\mathscr {P}\! \left[ \Omega _{TEM}^{*} \otimes \hat{S}_{src,el}\right]$. This changes for the correlated electrons, for which the convolution acts on the Poisson term itself $\Omega _{TEM}^{*} \otimes \mathscr {P}\! \left[ \hat{S}_{src,el}\right]$, since the electrons are influenced by the previous ones. We obtain the electron beam for the TEM mode $B_{TEM}$ consisting of both, correlated and uncorrelated electrons hitting the fluorescence layer of the CCD as:

42 $$\begin{aligned} B_{TEM} = p\cdot \left( \Omega _{TEM}^{*} \otimes \mathscr {P}\! \left[ \hat{S}_{src,el}\right] \right) + \left( 1-p\right) \cdot \mathscr {P}\! \left[ \Omega _{TEM}^{*} \otimes \hat{S}_{src,el}\right] \text { ,} \end{aligned}$$

where *p* gives the probability for correlated electrons within the beam. The broadening of the point-source to a parallel beam happens without additional gain. However, since we are interested in the broadened signal rather than the total beam intensity we define $\Omega _{TEM}^{*}$ to have the height of one, indicated by $*$. This is in contrast to all following convolutions, which are normalized to the sum of all entries. For this type of signal, we obtain the Pearson correlation coefficients following Eq. 36 as:

43 $$\begin{aligned} \rho _{TEM,m,n}&= \left[ \left( p\cdot \Omega _{TEM}^{*} + \left( 1-p\right) \cdot \delta \right) \otimes \left( p\cdot \Omega _{TEM}^{*} + \left( 1-p\right) \cdot \delta \right) \right] _{n,m} \nonumber \\&\approx \left[ p^{2}\cdot \Omega _{TEM}^{*}\otimes \Omega _{TEM}^{*} \, + \,2\cdot p\cdot \left( 1-p\right) \cdot \Omega _{TEM}^{*} + \left( 1-p\right) ^{2}\cdot \delta \right] _{n,m}\text{ ,} \end{aligned}$$

where $\delta$ is the Dirac delta, since the Poisson distributions of the uncorrelated electrons is unchanged by the beam deflection. For a sufficiently large beam disc $\Omega _{TEM}^{*}$ with respect to the detector, we can approximate the first term as a constant.

In the fluorescence layer of the detector, every incident electron produces a cascade of photons (see Fig. 1a). The generation of photons in the scintillation layer is rather complex, as multiple factors like thickness, reflections at the scintillator interface and scattering events, defects, etc. influence the electron path and thus shape the signal locally^1,6^. Verbeeck and Bertoni^5^ pointed out that under the assumption of a rather homogeneous fluorescence layer, this is negligible. Following Eq. 39, this process can be modeled as another convolution of the electron beam $B_{TEM}$ with a kernel $\Omega _{fl}$ and with a fluorescence gain $g_{fl}\gg 1$. This convolution affects both the Poisson statistics of the correlated and the uncorrelated electrons, since all electrons produce multiple photons, which are then spread by the convolution. This leads to a smoothing of the noise, as was shown in “Section Noise and convolution”. With $S_{el} = \Omega _{TEM}^{*}\otimes S_{src,el}$, we obtain:

44 $$\begin{aligned} g_{fl}\cdot \Omega _{fl} \otimes B_{TEM}= \mathscr {P}_{ g_{fl}\cdot \beta _{fl}\cdot \beta _{TEM}}\left[ \Omega _{fl}\otimes \hat{S}_{el}\right] \quad \text {, with} \quad \beta _{TEM}= \beta _{TEM,conv} \cdot \beta _{TEM,corr}\text{ }\nonumber\\ \text {and} \quad \; \beta _{fl}\quad\, =\beta _{fl,conv}\;\;\;\;\,\cdot \beta _{fl,corr}\;\;\; \text { .} \end{aligned}$$

As a result, we obtain a smoothing of the variance as described in Eqs. 37 and 22, which depends on the Pearson correlation coefficients. This smoothing of the noise by $\beta _{TEM,conv}$ and $\beta _{TEM,corr}$ as well as $\beta _{fl,conv}$ and $\beta _{fl,corr}$ is the one mentioned by several authors^5,6^. Every following spreading of the correlated signal will thus add on the PSF applied to the signal, as well as on the smoothing of the formerly true-Poisson noise, which changes into a super-Poisson distribution, as the gain $g_{fl}$ by far exceeds the smoothing.

After the fluorescence layer, the created photons are guided by the fiber optic, which absorbs or loses some of the photons, indicating a second gain $g_{opt}<1$ and an additional PSF $\Omega _{opt}$. The photons finally arrive at the CCD camera and are absorbed and converted back into charge carriers. As not every photon creates an exciton, this leads to a third gain $g_{CCD}<1$, also known as the Fano factor^40,41^. Obviously, the wells of the pixels are finite, thus creating a third PSF for the actual CCD camera $\Omega _{CCD}$. Implementing all these considerations into a formula, leads to a convolution of the detector kernel $\Omega _{d}$ with the different Poisson noise distributions of the electron beam $B_{TEM}$:

45 $$\begin{aligned} g_{d}\cdot \Omega _{d} \otimes B_{TEM}=\mathscr {P}_{\beta _{conv}\cdot \beta _{corr}\cdot g_{d}}\left[ \Omega _{d} \otimes \hat{S}_{el}\right] \qquad \text {, with} \qquad \Omega _{d}= \Omega _{fl}\otimes \Omega _{opt}\otimes \Omega _{CCD} \text { .} \end{aligned}$$

So a total detector gain value $g_{d} = g_{fl} \cdot g_{opt}\cdot g_{CCD}$ is obtained for the system as well as a smoothing factor for the convolution $\beta _{conv}= \beta _{TEM,conv}\cdot \beta _{fl,conv} \cdot \beta _{opt,conv}\cdot \beta _{CCD,conv}$ and a smoothing factor for the correlation $\beta _{corr}= \beta _{TEM,corr} \cdot \beta _{fl,corr} \cdot \beta _{opt,corr}\cdot \beta _{CCD,corr}$ altering the noise. We combine both to a total smoothing factor $\beta =\beta _{conv}\cdot \beta _{corr}$ for shorter notation. As a result of Eq. 38, we can simplify the individual convolution kernels of the layers to a combined kernel for the entire detector.

Correspondingly, we obtain the Pearson correlation coefficients of the image formation change from Eq. 43 into:

46 $$\begin{aligned} \rho _{m,n} \approx \left[ \Omega _{d}^{*}\otimes \Omega _{d}^{*}\otimes \left( p^{2}\cdot \Omega _{TEM}^{*}\otimes \Omega _{TEM}^{*} + 2\cdot p\cdot \left( 1-p\right) \cdot \Omega _{TEM}^{*} + \left( 1-p\right) ^{2}\cdot \delta \right) \right] _{n,m} \text { ,} \end{aligned}$$

where $\Omega _{d}^{*}$ describes the detector PSF normalized to the height of one. The convolution with a rather constant first term again yields a constant.

The gain acts as a conversion factor from incident beam electrons to charge carriers in the detector, with the detector signal given as $S_{d}=g_{d} \cdot S_{el}$. In a real-world system, quantum efficiencies vary in the fluorescence layer as well as in the CCD detector. Assuming that the direct path, rather than internal reflections, predominantly contributes to the intensity of a given detector pixel (*i*, *j*) and the others are negligible, the total gain can be written as an unknown distribution $\mathscr {X}$ varying around the mean value of all gains $G_{d,i,j} = g_{d} \cdot \frac{\mathscr {X} \left[ g\right] _{i,j}}{g_{d}}= g_{d} \cdot \mathscr {X} \left[ \bar{g}\right] _{i,j}$, with $\mathscr {X}\left[ \bar{g}\right] _{i,j} \in [0,\infty )$. The quantum efficiency variations are fixed and therefore are commonly referred to as fixed-pattern-noise^56,57^. This leads to the overall probability to measure *n* signal counts in a detector pixel:

47 $$\begin{aligned} \mathscr {P}_{\beta \cdot G_{d,i,j}}\left[ \Omega _{d} \otimes \hat{S}_{el}\right] _{i,j} &\approx \frac{ \left( g_{d}^{-1} \cdot \mathscr {X}\left[ \bar{g}\right] _{i,j}^{-1} \cdot \left[ \Omega _{d} \otimes \hat{S}_{d}\right] _{i,j}\right) ^{g_{d}^{-1}\mathscr {X}\left[ \bar{g}\right] _{i,j}^{-1}\beta ^{-1} \cdot n}}{\left( g_{d}^{-1} \cdot \mathscr {X}\left[ \bar{g}\right] _{i,j}^{-1} \cdot \beta ^{-1} \cdot n \right) !} \exp \left\{ -g_{d}^{-1} \cdot \mathscr {X}\left[ \bar{g}\right] _{i,j}^{-1}\cdot \beta ^{-1} \cdot n \right\} \quad\nonumber\\ & = g_{d}\cdot \mathscr {X}\left[ \bar{g}\right] _{i,j} \cdot \beta \cdot \mathscr {P}\left[ \frac{\left[ \Omega _{d} \otimes \hat{S}_{d}\right] _{i,j}}{g_{d}\cdot \mathscr {X}\left[ \bar{g}\right] _{i,j} \cdot \beta }\right] \end{aligned}\text{ ,} $$

which can be rescaled to a pure-Poisson distribution by factoring the gains and $\beta$ out (see Eq. 12). Taking into account the simplification that only the direct path contributes to the signal, we use the approximate sign.

Operating a CCD camera always produces heat, and even if the camera is cooled, this thermal energy is likely to produce excitons in the course of the acquisition time $t_{acq}$. The generation of these is known to produce dark currents $I_{dark}$ and the longer the acquisition time, the higher is the accumulated charge and thus the offset $\mu _{therm} =I_{dark} \cdot t_{acq}$ in charge^7^. These dark currents are especially favored by certain defects within the material^58^, which are not uniformly distributed across the CCD array, leading to another unknown distribution $\Upsilon \!\left[ \mu _{therm}\right] _{i,j}$ for the conversion of thermal energy into excitons. Owing to the quantized nature of charge, these thermal excitons further add Poisson noise^6,7,59^.

Finally, charges are converted into counts by the analog-digital-converter (ADC). This process is widely known to add further Gaussian distributed read-out noise to a measurement^6,7,60^. The Gaussian distribution is given in Eq. 1. To avoid negative counts, an external bias voltage offsets the read-out process^61^. Fluctuations of bias in between rows occur as row artifacts. We also consider these to be Gaussian distributed, with the mean value of the respective row $\mu _{row,j}$ and the variance $\sigma _{read}^{2}$ of the read-out noise. Here, the mean value itself varies from row to row with $\mathscr {N}\!\left[ \bar{\mu }_{read},\,\sigma _{row}^{2}\right]$. Combining both, the read-out noise can be written as $\mathscr {N}\!\left[ \bar{\mu }_{read}\, ,\,\sigma _{read}^{2} + \sigma _{row,j}^{2}\right]$.

With a conversion gain $g_{c}$ from charge carriers to counts, a total mean gain $g = g_{c} \cdot g_{d}$ and a total varying gain $G_{i,j} = g \cdot \mathscr {X} \left[ \bar{g} \right] _{i,j}$ can be found. The signal in counts is then given as $S_{c}=g_{c} \cdot S_{d} = g\cdot S_{el}$, where $S_{el}$ gives the electron signal of the beam and $S_{d}$ gives the detector charge carriers.

However, the gain of the pixels is not a constant, but changes with the intensity due to saturation effects besides other non-linearities^61,62^. As a pixel can be seen as a capacitor, higher levels of accumulated charge restrain the probability of creating additional excitons, thereby decreasing the overall gain. So, the gain of the system does not only depend on the camera system itself, but also on the level of the acquired signal, thus $\mathscr {X} \left[ \bar{g}\right] _{i,j} \rightarrow \mathscr {X} \left[ \bar{g}\!\left( S_{el}\right) \right] _{i,j}$.

Considering all of the above, we can express the image formation process of the image $\xi$ as follows:

48 $$\begin{aligned} \xi _{i,j} \approx g \cdot \mathscr {X}\!\left[ \bar{g}\!\left( \Omega _{d} \otimes \hat{S}_{el}\right) \right] _{i,j} \cdot \beta \cdot \mathscr {P}\! \underbrace{\left[ \frac{\left[ \Omega _{d} \otimes \hat{S}_{el}\right] _{i,j}}{\beta } \right] }_{ \textstyle \begin{matrix} = \frac{\left[ \Omega _{d} \otimes \hat{S}_{c}\right] _{i,j}}{g \cdot \mathscr {X}\!\left[ \bar{g}\!\left( S_{c}\right) \right] _{i,j}\cdot \beta } \end{matrix} } + \; g_{c} \cdot \left( \mathscr {P}\Big [\Upsilon \!\left[ \mu _{therm}\right] _{,i,j}\Big ] + \mathscr {N}\! \left[ \bar{\mu }_{read}\, ,\,\sigma _{read}^{2} + \sigma _{row,j}^{2} \right] \right) \text { ,} \end{aligned}$$

assuming homogeneous read-out noise across the CCD camera and an additional noise term in the vertical and only in the vertical direction of the image columns *i*, as it is fixed per row *j*. Using the convolution of noise Eq. 2, we can write the total noise as the sum of the individual noise distributions. It is important to note that the values for $\bar{\mu }_{read}$, $\sigma _{read}$, $\sigma _{row}$ and $\mu _{therm}$ vary between detector segments, as each segment has its own ADC (see Fig. 1b). In the following, we will abbreviate $\hat{S}_{\Omega ,el}= \left[ \Omega _{d}\otimes \hat{S}_{el}\right]$ to shorten notation.

We consider this image formation as the general case for our detector for measurements in TEM mode and under reserve for STEM mode, which we will elaborate on later.

### Processing operations

We have described the image formation process for a measurement on a typical scintillation-based CCD detector, but often the quality of the acquired images is enhanced by techniques, such as background subtractions, applying gain references or gain non-linearity corrections. All these corrections do influence the noise in the corrected images, which is the subject of this section.

#### Dark frame subtraction

**Fig. 4:**
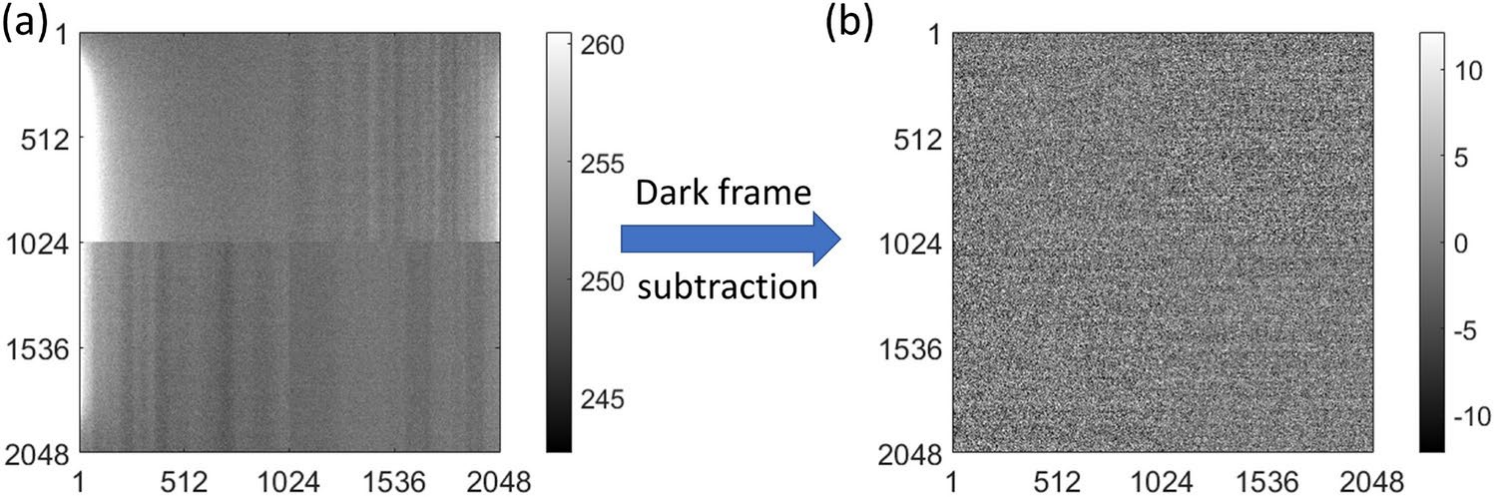
(**a**) Bias frame averaged across 30 unprocessed images without signal at zero exposure time. It can be seen that the image is offsetted by $\mu \approx \text {252}$ counts indicated by the grey value in between a 5$\sigma$ range displayed. Further, brighter areas can be spotted on the detector, where dark currents $\sigma _{therm}$ are increased and vertical columns are visible, where the Ohmic resistance changes currents. The four quadrants can be seen at their boundaries, where image features are interrupted. (**b**) After subtracting an image without signal, the result appears without offset, brighter areas are cleared and the image in general has fewer features than before subtraction. Horizontal lines become visible, where the bias of the entire line varies between read-out, because image variation in general decreases. Most of the image variation now is given by the read-out noise $\sigma _{read}$ changing between pixels on top of the row noise $\sigma _{row,j}$. This procedure of getting from (a) to (b) is referred to as ‘dark frame subtraction’.

To clear an image from the offset and additional dark currents, a second image is acquired with a closed shutter $\hat{S}_{el}= 0$, the so-called dark frame. This dark frame is then subtracted from the original image^6,7^ (see Fig. 4a,b), leading to:

49 $$\begin{aligned} \xi _{DS,i,j}=\xi _{i,j}-\xi _{dark,i,j} \qquad \text {, with} \qquad \xi _{dark,i,j}&= g_{c}\cdot \left( \mathscr {P}\Big [\Upsilon \!\left[ \mu _{therm}\right] _{i,j}\Big ] + \mathscr {N}\! \left[ \bar{\mu }_{read},\,\sigma _{read}^{2} + \sigma _{row,j}^{2} \right] \right) \text { .} \end{aligned}$$

Since both measurements are uncorrelated, the summation rule Eq. 2 can be utilized for the Gaussian distributed parts of Eq. 48. This increases read-out and row noise, but clears the image from the read-out offset $\bar{\mu }_{read}$. Further, subtracting Poisson distributions leads to a Skellam distribution (see Eq. 11) for the thermal noise. So, the image formation for a dark frame subtracted image is given as:

50 $$\begin{aligned} \!\!\!\! \xi _{DS,i,j} \approx g \cdot \mathscr {X}\!\left[ \bar{g}\!\left( \hat{S}_{\Omega ,el}\right) \right] _{i,j} \cdot \beta \cdot \mathscr {P}\! \left[ \frac{\hat{S}_{\Omega ,el,i,j}}{\beta } \right] + g_{c}\cdot \left( \mathscr {S}\Big [0\,,\,\Upsilon \left[ \hat{\mu }_{therm}\right] _{i,j},\Upsilon \left[ \hat{\mu }_{therm}\right] _{i,j}\Big ] + \mathscr {N}\! \left[ 0\, ,\,2\sigma _{read}^{2} + 2\sigma _{row,j}^{2} \right] \right) \text { ,} \end{aligned}$$

where the dark frame subtracted image $\xi _{DS,i,j}$ provides the counts of every pixel cleared by the offset.

Since the noise contribution of dark currents in a cooled CCD is rather small and the zero centered Skellam distribution with $S_{1}=S_{2}$ is symmetric, it can be (and usually is) approximated by a Gaussian distribution. Since $\Upsilon \!\left[ \mu _{therm}\right] _{i,j}$ is further assumed to be rather homogeneously distributed across the CCD, this yields:

51 $$\begin{aligned} \xi _{DS,i,j} \approx g \cdot \mathscr {X}\!\left[ \bar{g}\!\left( \hat{S}_{\Omega ,el}\right) \right] _{i,j} \cdot \beta \cdot \mathscr {P}\! \left[ \frac{\hat{S}_{\Omega ,el,i,j}}{\beta } \right] + \mathscr {N}\! \left[ 0\, ,\,2\sigma _{read}^{2} + 2\sigma _{row,j}^{2} + 2\sigma _{therm}^{2}\right] \text { ,} \end{aligned}$$

with $\sigma ^{2}_{therm}=g_{c}\cdot \mu _{therm}$ depending on the dark current $\mu _{therm}$. The factor of 2 reflects the fact that two images are subtracted from each other. However, it shall be noted that the Skellam distribution has larger tails than the Gaussian distribution. $\Upsilon \!\left[ \mu _{therm}\right] _{i,j}$ might also deviate locally due to the construction of the CCD, but as the noise contribution is generally low, the effect is negligible. To clear notation, in the following read-out, row and thermal noises will be referred to as ‘detector noise’:

52 $$\begin{aligned} \sigma _{d}^{2} = \sigma _{read}^{2} +\sigma _{row,j}^{2}+\sigma _{therm}^{2} \text { .} \end{aligned}$$

#### The acquisition of a gain reference

Detectors are often calibrated with respect to their gain distribution to compensate for variations in the quantum efficiency at each pixel. Generally, the gain reference^6,7^ is acquired with high counts and a uniform signal across the detector, which can easily be achieved in TEM mode by expanding the beam. The resulting signal frame image $\xi _{ref,SF}$ is then dark frame subtracted by a dark frame image $\xi _{ref,DF}$ and divided by the mean value of the image. The gain reference image formation of a scintillation-based CCD detector thus can be modeled as:

53 $$\begin{aligned} \xi _{ref,i,j} \approx \frac{ \mathscr {X}\!\left[ \bar{g}\!\left( S_{ref}\right) \right] _{i,j}\cdot g \cdot \beta \cdot \mathscr {P}\! \left[ \frac{\hat{S}_{ref}}{\beta } \right] + \mathscr {N}\!\left[ 0 \, ,\,2\sigma _{d}^{2} \right] }{g \cdot \bar{S}_{ref} } \text { ,} \end{aligned}$$

with the convolution of a homogeneous electron signal $\Omega _{d} \otimes \hat{S}_{ref}=\hat{S}_{ref}$ having no significant effect other than for correlation and noise smoothing as described in “Sections Noise correlation effects” and “Noise and convolution”. The noise term $\mathscr {N}\!\left[ 0 ,\,2\sigma _{d}^{2} \right]$ describes the dark frame subtracted detector noise. The denominator is given by the mean value of the reference signal $\bar{S}_{ref} \rightarrow \hat{S}_{ref}$ approaching its expectation value for large enough pixel sets and a high reference signal. Both assumptions hold for typical CCD cameras and gain calibration procedures. To mitigate the impact of saturation on deviations in the quantum efficiencies, this procedure is performed at $\sim$1/10 of the maximum count. As some pixels generate more counts per incident electron than others, they saturate faster, which leads to an underestimation of the quantum deviations at lower signal strengths. To maintain high signal strength, *w* images can be added up, increasing the signal but also the noises:

54 $$\begin{aligned} \xi _{ref,i,j} \approx \frac{ \mathscr {X}\!\left[ \bar{g}\!\left( \hat{S}_{ref}\right) \right] _{i,j}\cdot g \cdot \beta \cdot \mathscr {P}\! \left[ \frac{\sum _{w} \hat{S}_{ref}}{\beta } \right] + \mathscr {N}\!\left[ 0 \, ,\,2\, \sum _{w} \sigma _{d}^{2} \right] }{g \cdot \sum _{w} \hat{S}_{ref} } \text { ,} \end{aligned}$$

according to the summation rules for Gaussian and Poisson distributions in Eqs. 2 and 10. Further, we can approximate $\mathscr {X}\!\left[ \bar{g}\!\left( S_{ref}\right) \right] _{i,j} \rightarrow \mathscr {X}\!\left[ \bar{g}\!\left( \hat{S}_{ref}\right) \right] _{i,j}$ as the signal approaches its expectation value $S_{ref} \rightarrow \hat{S}_{ref}$ across several measurements *w*. The overall probability to measure a gain reference that resembles the quantum efficiency differences $\mathscr {X}\!\left[ \bar{g}\!\left( \hat{S}_{ref}\right) \right] _{i,j}$ is given as the convolution (see Eq. 2) of the Poisson distribution with the normal distributed part:

55 $$\begin{aligned} \Pr \left[ \xi _{ref,i,j}\!=\! \mathscr {X}\!\left[ \bar{g}\!\left( \hat{S}_{ref}\right) \right] _{i,j} \cdot \zeta _{ref,i,j} \right] \approx \mathscr {X}\!\left[ \bar{g}\!\left( \hat{S}_{ref}\right) \right] _{i,j} \cdot \frac{ g \cdot \beta \cdot \mathscr {P}\! \left[ \frac{\sum _{w} \hat{S}_{ref}}{\beta } \right] \otimes \mathscr {N}\!\left[ 0 \,,\,2\cdot \sum _{w} \sigma _{d}^{2}\right] }{g \cdot \sum _{w} \hat{S}_{ref}} \text { .} \end{aligned}$$

As for high signals, the Poisson distribution is in good approximation equal to the normal distribution (see Eq. 9). It can be rewritten:

56 $$\begin{aligned} \Pr \left[ \xi _{ref,i,j}\! =\! \mathscr {X}\!\left[ \bar{g}\!\left( \hat{S}_{ref}\right) \right] _{i,j} \cdot \zeta _{ref,i,j} \right] \approx \mathscr {X}\!\left[ \bar{g}\!\left( \hat{S}_{ref}\right) \right] _{i,j} \cdot \frac{ g \cdot \beta \cdot \mathscr {N}\!\left[ \frac{\sum _{w} \hat{S}_{ref}}{\beta }\,,\,\frac{\sum _{w} \hat{S}_{ref}}{\beta }\right] \otimes \mathscr {N}\!\left[ 0 \,,\,2\cdot \sum _{w} \sigma _{d}^{2}\right] }{g \cdot \sum _{w} \hat{S}_{ref}}\text { .} \end{aligned}$$

**Fig. 5:**
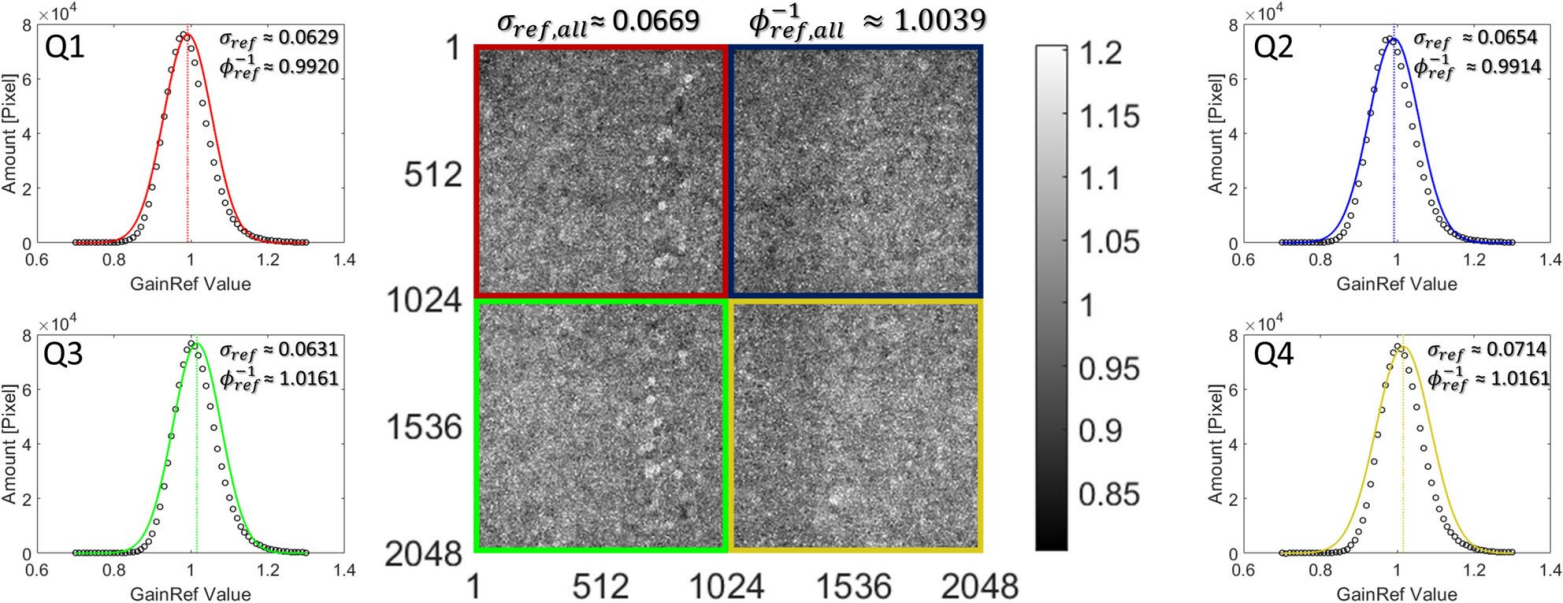
Gain reference of our *US1000FT-XP 2* detector in the middle, acquired by adding up 30 images under homogeneous illumination conditions in TEM mode and then subtracting 30 dark frame images with the same exposure of $t \approx 0.85\, \text {s}$. The mean intensity of each image was targeted at $\hat{S}_{ref} \approx$ 7050 counts. Due to differences in the glue and the fluorescence layer in the manufacturing, the gain reference shows stripes superimposed to artifacts from the fiber optics. The gain reference is surrounded by the individual frequency distributions of the gain values in the four detector quadrants Q1-Q4, where each dot represents an interval of 0.01 around its center. Since the gain reference normalizes with respect to all quadrants, the respective mean values of the quadrants differ from 1. The standard deviations $\sigma \approx 0.067$ are similar for all quadrants indicating a rather homogeneous gain distribution across the detector, however the aforementioned stripes and artifacts lead to small differences. Overall, the gain distributions are slightly skewed to the right for all quadrants, as a result of the inversion. Therefore, the overall mean of the inverse gain reference differs slightly from 1 (see Eq. 7).

Again, by utilizing the convolution rules of two Gaussian distributions Eq. 2 and the multiplication by a constant in Eq. 3, this allows to simplify the resulting normal distribution to:

57 $$\begin{aligned}  \Pr \left[ \xi _{ref,i,j} = \mathscr {X}\!\left[ \bar{g}\!\left( \hat{S}_{ref}\right) \right] _{i,j} \cdot \zeta _{ref,i,j} \right] \approx \mathscr {X}\!\left[ \bar{g}\!\left( \hat{S}_{ref}\right) \right] _{i,j} \cdot \mathscr {N}\!\left[ 1,\,k_{ref,i,j}^{2}\right] \;\;\, \text{, with} \nonumber \\ k_{ref,i,j}^{2} =  \frac{\sum _{w} g^{2} \cdot \beta \cdot \hat{S}_{ref} + 2\cdot \sum _{w} \sigma _{d}^{2}}{\left( g\cdot \sum _{w} \hat{S}_{ref}\right) ^{2}}\quad\;\;\,\text{ ,}\quad\;\;\;\;\, \end{aligned}$$

where the measured signal in counts is given as $\hat{S}_{ref,c} = g\cdot \hat{S}_{ref}$. Further, by approximating the local $k_{ref,i,j} \approx k_{ref}$ by their overall mean value and by taking the inverse distribution, following Eq. 7, we obtain:

58 $$\begin{aligned} \xi _{ref,i,j}^{-1}\!\left( \hat{S}_{ref}\right) = \mathscr {X}\!\left[ \bar{g}\!\left( \hat{S}_{ref}\right) \right] _{i,j}^{-1} \cdot \zeta _{ref,i,j}^{-1}  \quad \text {, with}\end{aligned}$$

59 $$\begin{aligned} \quad\quad \;\;\zeta _{ref,i,j}^{-1} \in \mathscr {N}\left[ 1,\,k_{ref}^{2} \right] \in [0,\,\infty )\quad\;\;\,\text{ ,}\quad\;\;\end{aligned}$$

where $\zeta _{ref,i,j}^{-1}$ is the actual representation of the normal distribution and $k_{ref}$ describes the uncertainty of the gain reference. This gain reference (see Fig. 5) can now be applied to individual images via multiplication, which is faster in processing as divisions. It is important to note that the gain reference is acquired across all detector segments altogether and it is to be assumed that the real gain of each quadrant slightly differs from the others. This results in slightly different *k*-values for any subset of pixels differing from the full detector, as it is the case for EELS in particular. At this point, the acquired gain reference is only valid for a specific intensity $\hat{S}_{ref}$, as deviating from this intensity necessarily shifts the individual gain values for all pixels due to the non-linearity of the detector.

#### The application of a gain reference

Now, that a suitable gain reference has been found, it needs to be applied to images. In general, applying the gain reference frame results in ratio distributions, whose probability density functions are complicated and for which the standard deviation is not always defined. For sufficiently low values of $k_{ref}$, which is a basic requirement of a good gain reference, Eq. 7 is valid and the overall Gaussian of the multiplicand becomes quite narrow. So narrow that is does not change the overall shape of the respective noise distribution significantly, except for slightly broadening its variance.

With the above considerations, a gain reference like the one displayed in Fig. 5 can be applied to dark frame subtracted images (see Eq. 51), which leads to the gain normalized image $\xi ^{*}$, given as:

60 $$\begin{aligned} \xi ^{*}_{i,j} = \xi _{DS,i,j} \cdot \xi _{ref,i,j}^{-1}\!\left( \hat{S}_{ref}\right) \approx \frac{\mathscr {X}\!\left[ \bar{g}\!\left( S_{\Omega ,el}\right) \right] _{i,j}}{\zeta _{ref,i,j} \cdot \mathscr {X}\!\left[ \bar{g}\!\left( \hat{S}_{ref}\right) \right] _{i,j}} \cdot g \cdot \beta \cdot \mathscr {P}\! \left[ \frac{\hat{S}_{\Omega ,el,i,j}}{\beta } \right] + \frac{\mathscr {N}\!\left[ 0 \,,\,2 \sigma _{d}^{2}\right] }{\xi _{ref,i,j}\!\left( \hat{S}_{ref}\right) } \text { ,} \end{aligned}$$

with $\zeta _{ref}^{-1}$ describing the ‘new’ fixed-pattern noise induced by gain normalization, whereas the gain reference is printed into the background noise of the detector.

To see how the application of the gain reference influences the different noises, we separate the above equation into two separate parts, the Poisson distributed signal part and the Gaussian distributed detector noise part. Starting with the influence on the Gaussian distributed detector noises of Eq. 60, we can assume a rather homogeneous distribution of the quantum efficiencies across the detector and roughly approximate $\xi _{ref}^{-1} \approx \mathscr {N} \left[ \phi _{ref}^{-1}\, , \, \frac{\sigma ^{2}_{ref}}{\phi _{ref}^{4}}\right]$, with $\sigma ^{2}_{ref}\approx \sigma _{QE}^{2} + k_{ref}^{2}$. Here, $\phi _{ref}^{-1}$ is the mean value of the inverse gain reference of each specific segment (see Fig. 5). Due to inhomogenieties in the distribution of the gains, these mean values slightly differ from one. The detector noises are given as Eq. 52. From Eq. 6 follows that:

61 $$\begin{aligned} \xi _{ref}^{-1} \cdot \mathscr {N}\!\left[ 0 \,,\,2 \sigma _{d}^{2} \right] \approx \mathscr {N}\!\left[ 0 \,,\, \left( \phi _{ref}^{-2} + \frac{\sigma ^{2}_{ref}}{\phi _{ref}^{4}}\right) \cdot 2 \sigma _{d}^{2}\right] \text { ,} \end{aligned}$$

for each of the four quadrants.

Now, that the effect of the gain reference on the detector noise has been shown, it is to show how it influences the signal noise of Eq. 60. Assuming, for a moment, that the measured image intensity is in close proximity to the image intensity of the gain reference, we can approximate $\mathscr {X}_{R,i,j}= \frac{\mathscr {X}\!\left[ \bar{g}\!\left( S_{\Omega ,el}\right) \right] _{i,j}}{\mathscr {X}\!\left[ \bar{g}\!\left( \hat{S}_{ref}\right) \right] _{i,j}} \approx 1$. We will further elaborate on this term in the next section. Thus, we are left with:

62 $$\begin{aligned} \mathscr {X}_{R,i,j}\cdot \zeta _{ref,i,j}^{-1} \cdot g \cdot \beta \cdot \mathscr {P}\! \left[ \frac{\hat{S}_{\Omega ,el,i,j}}{\beta } \right] = \mathscr {X}_{R,i,j}\cdot \mathscr {N}\left[ 1 \,,\,k_{ref}^{2} \right] \cdot g \cdot \beta \cdot \mathscr {P}\! \left[ \frac{\hat{S}_{\Omega ,el,i,j}}{\beta } \right] \text { .} \end{aligned}$$

By utilizing the Gaussian approximation of the Poisson distribution (see Eq. 9) and the multiplication of distributions (see Eq. 6) it can be shown that:

63 $$\begin{aligned} \mathscr {N}\left[ 1 \,,\,k_{ref}^{2} \right] \cdot g \cdot \beta \cdot \mathscr {P}\! \left[ \frac{\hat{S}_{\Omega ,el,i,j}}{\beta } \right] \approx g \cdot \beta \cdot \mathscr {P}\! \left[ \frac{\hat{S}_{\Omega ,el,i,j}}{\beta } \right] + \mathscr {N} \Big [0\,,\,k_{ref}^{2}\cdot \left( g^{2} \cdot \hat{S}_{\Omega ,el,i,j}^{2} + g^{2}\cdot \beta \cdot \hat{S}_{\Omega ,el,i,j}\right) \Big ] \text { ,} \end{aligned}$$

i.e. the multiplication by the gain reference deviates the expectation value and the variance $\hat{S}_{el}$ of the Poisson distribution by its variance $k_{ref}^{2}$. Combining the results of Eqs. 61 and 63, we can rewrite Eq. 60 as:

64 $$\begin{aligned} \xi ^{*}_{i,j} \approx \mathscr {X}_{R,i,j} \cdot g \cdot \beta \cdot \mathscr {P}\! \left[ \frac{\hat{S}_{\Omega ,el,i,j}}{\beta } \right] + \mathscr {N} \Big [0\,,\,\mathscr {X}_{R,i,j}^{2}\cdot k_{ref}^{2}\cdot \left( g^{2} \cdot \hat{S}_{\Omega ,el,i,j}^{2} + g^{2}\cdot \beta \cdot \hat{S}_{\Omega ,el,i,j}\right) \Big ] + \mathscr {N}\!\left[ 0 \,,\, 2 \sigma _{d}^{*\,2}\right] \text { ,} \end{aligned}$$

with the gain normalized detector noise:

65 $$\begin{aligned} \sigma _{d}^{*\,2} = \left( \phi _{ref}^{-2} + \frac{\sigma ^{2}_{ref}}{\phi _{ref}^{4}}\right) \cdot \sigma _{d}^{2} \text{ ,} \end{aligned}$$

where $\phi$ is the mean value of the respective quadrant varying the detector noises between quadrants and $\sigma _{ref}$ is the standard deviation of the acquired gain reference frame.

To make the gain reference independent of the signal strength of the measurement, saturation and other non-linearity effects must be corrected for in both gain reference and signal.

#### Gain non-linearities

Often, images cover a large dynamic range. Especially in measurements like EELS, we obtain very high intensities in the zero-loss peak (ZLP) and comparably small signals of interest. To gather enough statistic for the signals, the ZLP often reaches into the domain, where saturation effects beside other non-linearities are observed. This leads to deviations between the original and the measured signal intensity. Also, the gain reference, to this point, is only valid for a limited range around the target intensity it was acquired with. Especially for EELS, this is undesirable, as the gain reference is only valid for a small portion of the signal. Thus, it is important to find a general correction for the detector, which linearizes the gain throughout the entire dynamic range.

Adjusting the gains to counteract non-linearities generally changes the noise. To see the actual effects, we need to reconsider Eq. 64, especially with respect to $\mathscr {X}_{R,i,j}$. Due to differences in the thickness in the fluorescence layer and different quantum efficiencies, some pixels collect more charge than others. Considering a large range of signals, some pixels saturate more than others, which leads to deviations between the gain reference and the actual gain distribution represented in another experiment. So, not only the actual measurement but also the gain reference must be corrected for non-linearities. To compensated with an unknown factor $g_{lin,i,j}$, defined to removes the dependency of the gain on the signal level $\hat{S}$, linearizing the gain for the entire dynamic range, we must rescale the intensity of every pixel by its non-linearity correction function. We obtain:

66 $$\begin{aligned} \mathscr {X}_{R,i,j}= \frac{\mathscr {X}\!\left[ g_{lin}\!\left( S_{\Omega ,el}\right) \cdot \bar{g}\!\left( S_{\Omega ,el}\right) \right] _{i,j}}{\mathscr {X}\!\left[ g_{lin}\!\left( \hat{S}_{ref}\right) \cdot \bar{g}\!\left( \hat{S}_{ref}\right) \right] _{i,j}} = \frac{\mathscr {X}\!\left[ \bar{g}\right] _{i,j}}{\mathscr {X}\!\left[ \bar{g}\right] _{i,j}} = 1 \text { .} \end{aligned}$$

Assuming that the gain deviations inside the CCD are much smaller than in the fluorescence layer and fiber optics, we can approximate the linearization correction $g_{lin}$ to be equal for all pixels. We thus obtain a mean photon transfer curve (PTC) for the entire detector as the inverse of $g_{lin}$.

Usually, non-linearities of the detector gain are fitted by a second or third order polynomial as a function of signal strength $g\left( \hat{S}_{c}\right) = g\cdot \left( \hat{S}_{c} - x_{1} - x_{2}\cdot \hat{S}_{c}^{2} - x_{3}\cdot \hat{S}_{c}^{3}\right)$^63^. Here, the coefficients $x_{1,2,3}$ represent the fit parameters. As the signal is subject to different noises, it is better described with the measurement $\xi _{i,j}$ instead of $\hat{S}$, such that a correction factor is given as:

67 $$\begin{aligned} g_{lin}\!\left( \xi _{i,j}\right) = \frac{g\cdot \xi _{i,j}}{g\cdot \left( \xi _{i,j} - x_{1} - x_{2} \cdot \xi _{i,j}^{2} - x_{3}\cdot \xi _{i,j}^{3}\right) }\text { .} \end{aligned}$$

Note that this correction applies to the original measurement without background subtraction, since the offset must be considered for the correct total count.

As the correction factor is obtained by fitting measurements, it is again subject to deviations between fit and the actual gains. Particularly, as the saturation may vary from pixel to pixel. Based on the previous argumentation in this work, it can easily be anticipated that these deviations in the gain appear squared in the noise, which consequently leads to second and forth order terms connected to the Poisson term and to forth and eighth order deviations in the Gaussian term connected to the deviations induced by the gain reference. To maintain reasonably clear noise model, we only consider a second order term and neglect the higher ones. Note however that these deviation of the gain linearization $k_{lin}^{2}$ do not add (much) to the image volatility per se. They rather alter the linearity of the gain throughout the dynamic range. So, it depends on the difference between intensities that are compared. For normal imaging as well as for EELS, $k_{lin}^{2}$ can be neglected and the corresponding noise term is well below the Poisson noise, as we will see later. However, we require this term for a precision analysis for the binning of detector noises, where it will appear as a small offset to the uncertainty of the gain reference. This is the sole reason for its inclusion here.

For a gain normalized and non-linearity corrected image $\xi ^{corr}= \frac{g_{lin}\left( \xi \right) \cdot \xi _{DS}}{g_{lin}\left( \xi _{ref,org}\right) \cdot \xi _{ref}}$, where $\xi _{ref,org}$ denotes the original measurements used for the gain reference and $\xi _{DS}$ is the dark frame subtracted image from Eq. 51, we can write instead of Eq. 64:

68 $$\begin{aligned} \xi ^{corr}_{i,j}\approx & g \cdot \beta \cdot \mathscr {P}\! \left[ \frac{\hat{S}_{\Omega ,el,i,j}}{\beta } \right] + \mathscr {N} \Big [0,\,k^{2}\cdot g^{2} \cdot \hat{S}_{\Omega ,el,i,j}^{2} + k^{2} \cdot g^{2}\cdot \beta \cdot \hat{S}_{\Omega ,el,i,j}\Big ] + \mathscr {N}\!\left[ 0,\, 2 \sigma _{d,corr}^{2}\right] \quad\;\, \text{, with} \end{aligned}$$

69 $$\begin{aligned}  \sigma _{d,corr}^{2} \approx \bar{g}_{lin}^{2}\cdot \sigma _{d}^{*\,2} \qquad \text {and} \qquad \bar{g}_{lin}=\frac{1}{NM}\sum _{i,j}g_{lin,i,j} \qquad \text {as well as} \qquad k^{2}= k_{ref^{*}}^{2} + k_{lin}^{2} \text{ ,} \end{aligned}$$

where the factor *k* includes the uncertainties of the non-linearity corrected gain reference $k_{ref^{*}}$ and the deviation of the gain linearization $k_{lin}$. We utilized Eq. 6 for the multiplication of $\sigma _{d,corr}^{2}$ from Eq. 65 with $g_{lin}$. For the latter, we neglected the contribution of its variance term, since it depends on the distribution of the signal and the overall influence is estimated to be quite small.

Another effect, known as ‘brighter-fatter effect’^64,65^, describes the increase in the detector PSF with increasing signal $\Omega \rightarrow \Omega \left( S\right)$ due to diffusion of charge carriers between pixels. This effect leads to the observation that brighter objects appear larger on the CCD than darker objects, despite being the same size. Following Eq. 37, describing the smoothing of the noise due to convolution $\beta _{conv}$, and Eq. 22, describing the smoothing of the noise due to correlation $\beta _{corr}$, a broader PSF leads to a reduction of the variance described by the smoothing factor $\beta =\beta _{conv}\cdot \beta _{corr}$ and thus to the observation of a reduced smoothed gain $\beta \cdot g$. Thus, we must rewrite the smoothing factor $\beta \rightarrow \beta \!\left( S\right)$. This effect is reported to be reduced by binning or summation of neighboring pixels^64^, such as it is the case for EEL-spectra in one dimension. As it is impossible to differentiate the gain from the smoothing within this experimental setup, we speak of a smoothed gain here.

We neglect the additional convolution for simplicity, but describe the effect on the noise by:

70 $$\begin{aligned} \xi ^{corr}_{i,j} \approx g \cdot \beta \cdot \beta _{BF} \cdot \mathscr {P}\! \left[ \frac{\hat{S}_{\Omega ,el,i,j}}{\beta \cdot \beta _{BF}} \right] + \mathscr {N} \Big [0\,,\,k^{2}\cdot g^{2} \cdot \hat{S}_{\Omega ,el,i,j}^{2} + k^{2} \cdot g^{2}\cdot \beta \cdot \beta _{BF}\cdot \hat{S}_{\Omega ,el,i,j}\Big ] + \mathscr {N}\!\left[ 0 \,,\, 2 \sigma _{d,corr}^{2}\right] \text { .} \end{aligned}$$

As we will show in the following evaluation, the additional convolution induced by the brighter-fatter effect is rather small. It is negligible, even in high-count regimes, where the effect is most pronounced. Its impact on the variance, however, is important when measuring non-linearity effects with the signal-to-variance method. It must be corrected for in order to obtain the valid non-linearity correction $g_{lin}\!\left( \xi _{i,j}\right)$^63^.

### Binning of the detector

Detector binning is often employed to reduce detector noises. When binning the detector, two or more neighboring pixels are transferred to the ADC and are cumulatively read out, causing the binned pixels to appear as one. Relative to signal strength, the detector noises are reduced by binning.

However, due to correlation effects the signal noises change. This is the reason why manufacturers like *Gatan* acquire a set of different gain references for the most important binning settings. To maintain consistency, we focus on post-binning of the images, like it is performed for e.g. EELS measurements. In contrast to regular binning, post-binning does not reduce detector noises, but it alters the signal noise in the same manner as the regular binning.

Following Eq. 70, vertically summing *V* pixel along the columns and horizontally summing *H* pixel along the rows, can be written as:

71 $$\begin{aligned} \xi _{Bin,H,V}^{corr} \approx \sum _{i,j}^{H,V} \bigg (g \cdot \beta \cdot \beta _{BF} \cdot \mathscr {P}\! \left[ \frac{\hat{S}_{\Omega ,el,i,j}}{\beta \cdot \beta _{BF}} \right] + \mathscr {N} \Big [0\,,\,\underbrace{k^{2}\cdot g^{2} \cdot \hat{S}_{\Omega ,el,i,j}^{2}}_{1.} + \underbrace{k^{2}\cdot g^{2}\cdot \beta \cdot \beta _{BF}\cdot \hat{S}_{\Omega ,el,i,j}}_{2.} + \underbrace{2 \sigma _{d,corr}^{2}}_{3.}\Big ]\bigg )\text { .} \end{aligned}$$

Again, we can separate the noise into different parts and treat the summations independently.

Utilizing the addition of Poisson distributions (see Eq. 10) on the first part of Eq. 71 leads to:

72 $$\begin{aligned} \sum _{i,j}^{H,V} g \cdot \beta \cdot \beta _{BF} \cdot \mathscr {P}\! \left[ \frac{\hat{S}_{\Omega ,el,i,j}}{\beta \cdot \beta _{BF}} \right] = g \cdot \beta _{H,V}\cdot \beta _{BF,H,V} \cdot \mathscr {P}\! \left[ \frac{\sum _{i,j}^{H,V}\hat{S}_{\Omega ,el,i,j}}{\beta _{H,V}\cdot \beta _{BF,H,V}} \right] \text { ,} \end{aligned}$$

with the column- and row-wise addition of the signal removing some of the noise correlation induced by the detector PSD. This leads to a reduced smoothing factor $\beta _{H,V} = \beta _{conv,H,V}\cdot \beta _{corr,H,V}$, where *H* and *V* denote the summed pixels or binning values in the respective direction. As described in Eq. 36, convolution leads to correlation, which can be described by the Pearson correlation coefficients. According to Eqs. 26 and 27, these coefficients change under summation. The smoothing due to the convolution $\beta _{conv}$ changes according to Eq. 37, as part of the convolution is removed by summation. Further, the smoothing due to correlation $\beta _{corr}$ changes according to Eq. 22. As a result, we obtain a change for the smoothing factor $\beta _{H,V}$. The same happens to the brighter-fatter smoothing $\beta _{BF,H,V}$.

Summing up the variations of the signal introduced by the fixed-pattern noise of the gain reference (see Eq. 71 1.), however, is more complex, since the column distribution is lost by that summation. As the signal is not uniformly distributed across a given pixel column or row, some pixels contribute more to the signal than others. To address this this, we introduce a distribution factor $\alpha _{H,V}$. Since $k^{2}$ can be assumed to be rather homogeneously distributed across the detector, every pixel adds a fraction of $k^{2}$ equal to its contribution to the overall signal. So, we can sum up the fist part of the normal distribution of Eq. 71 1. as following:

73 $$\begin{aligned} & \sum _{i,j}^{H,V} k^{2} \cdot g^{2} \cdot \hat{S}_{\Omega ,el,i,j}^{2} = k^{2}\cdot g^{2} \cdot \sum _{i,j}^{H,V} \hat{S}_{\Omega ,el,i,j}^{2} = \alpha _{H,V}\cdot \underbrace{\left( k_{ref^{*},H,V}^{2}+k_{lin}^{2}\right) }_{= \,k_{H,V}^{2}} \cdot g^{2} \cdot \left( \sum _{i,j}^{H,V} \hat{S}_{\Omega ,el,i,j}\right) ^{2} \quad \text{, with} \end{aligned}$$

74 $$\begin{aligned}\qquad\qquad\qquad\qquad\qquad\quad\quad\;\;\;\,\alpha _{H,V} = \sum _{i,j}^{H,V} \left( \frac{\hat{S}_{\Omega ,el,i,j}}{\sum _{i,j}^{H,V} \hat{S}_{\Omega ,el,i,j}}\right) ^{2} \qquad\qquad\qquad\qquad\qquad\;\, \text {.} \end{aligned}$$

Under typical conditions, the image signal can be considered rather similar between neighboring pixels and for small binning values, allowing us to often neglect $\alpha _{H,V}\approx 1$. However, for large binning values, as required for EELS, $\alpha _{H,V}$ must be taken into consideration.

As the measurement of the non-linearity corrected gain reference $k_{ref^{*}}^{2}\rightarrow k_{ref^{*},H,V}^{2}$ is also correlated, due to beam correlations and the detector PSF, the same procedure as for the Poisson part has to be applied here, too. Taking a look into Eq. 57 reveals that $k_{ref,H,V}^{2}$ needs an update for the change in the smoothing factor $\beta =\beta _{conv}\cdot \beta _{corr}$, but we observe a change in the expectation value of the non-linearity corrected reference signal $\hat{S}_{ref}^{*}$ as well, due to the summation of pixel intensities during binning. We obtain:

75 $$\begin{aligned} k_{ref^{*},H,V}^{2}= \frac{\sum _{w} g^{2} \cdot \beta _{H,V} \cdot \sum _{i,j}^{H,V}\hat{S}_{ref}^{*} + 2\cdot \sum _{w}\sum _{i,j}^{H,V} \bar{g}_{lin}^{2}\cdot \sigma _{d}^{2}}{\left( g\cdot \sum _{w}\sum _{i,j}^{H,V}\hat{S}_{ref}^{*}\right) ^{2}} \text { ,} \end{aligned}$$

for the binned uncertainty of the non-linearity corrected gain reference, where $\bar{g}_{lin}$ is the mean value of the linearization factor $g_{lin,i,j}$, as shown in Eq. 69.

The factor $k_{lin}$ giving the uncertainty of the linearization correction, however, remains unchanged under summation and thus we obtain $k_{H,V}^{2}$ as defined above.

Similar changes can be found in the summation of the second part of the normal distribution in Eq. 71 2., which describes the variation of the Poisson noise by the fixed-pattern noise of the gain reference, which is given as:

76 $$\begin{aligned} \sum _{i,j}^{H,V} k^{2} \cdot \beta \cdot \beta _{BF} \cdot g^{2}\cdot \hat{S}_{\Omega ,el,i,j} = k_{H,V}^{2} \cdot g^{2}\cdot \beta _{H,V}\cdot \beta _{BF,H,V}\cdot \sum _{i,j}^{H,V}\hat{S}_{\Omega ,el,i,j} \text { .} \end{aligned}$$

The distribution factor $\alpha _{H,V}$ is not required here, since the noise is independent of the distribution of the signal, but just a mere variation of the Poisson noise.

The summation of the detector noise $\sigma _{d,corr}$ in the last part of the normal distribution of Eq. 71 3. is straightforward following Eq. 69, which describes the non-linearity corrected, gain normalized detector noise, and the summation rule of Eq. 25.

Combining the results of the summation of the Poisson noises in Eq. 72, the summation of the signal variation in Eq. 73 and the Poisson noise variation by the gain reference in Eq. 76, along with the addition of detector noises, the vertically binned image and its noises are given as:

77 $$\begin{aligned} \xi _{Bin,H,V}^{corr}&\approx g \cdot \beta _{H,V} \cdot \beta _{BF,H,V} \cdot \mathscr {P}\! \left[ \frac{\sum _{i,j}^{H,V}\hat{S}_{\Omega ,c,i,j}}{ g \cdot \beta _{H,V}\cdot \beta _{BF,H,V}}\right] + \mathscr {N}\!\left[ 0,\, \sigma _{Bin,H,V}^{2}\right] \qquad\qquad\qquad\qquad \text {, with} \\ \sigma _{Bin,H,V}^{2}&= \underbrace{\alpha _{H,V}\cdot k_{H,V}^{2}\cdot \left( \sum _{i,j}^{H,V} \hat{S}_{\Omega ,c,i,j}\right) ^{2}}_{1.}+ \underbrace{k_{H,V}^{2}\cdot g \cdot \beta _{H,V}\cdot \beta _{BF,H,V}\cdot \sum _{i,j}^{H,V} \hat{S}_{\Omega ,c,i,j}}_{2.} + \underbrace{2\sum _{i,j}^{H,V} \sigma _{d,corr}^{2}}_{3.} \text{ ,} \nonumber\end{aligned}$$

where we convert the signal back to counts $\hat{S}_{\Omega ,c}=g\cdot \hat{S}_{\Omega ,el}$, as this is how the microscope presents the results. Note that the row noise $\sigma _{row,j}$ contained in $\sigma _{d,corr}$ is constant along the rows and thus adds up quadratically.

We consider this our noise model for TEM measurements under binning, from which we obtain the noise model of a regular image for $H=1$ and $V=1$. For the sake of clarity, we provide a concise summary of the involved variables. *g* is the overall gain of the detector, which encompasses the gain of the fluorescence layer $g_{fl}>1$, the gain of the fiber optics $g_{opt}<1$, the gain of the CCD detector $g_{CCD}<1$, and a conversion gain $g_{c}$ into counts. Due to the broadening of the signal by the detector PSF, the Poisson noise is smoothed and therefore reduced by a smoothing factor $\beta$. This factor incorporates the smoothing of the noise by the convolution with the detector PSF $\beta _{conv}$ and smoothing that occurs by the effect of the correlation $\beta _{corr}$ induced by this convolution. Since the width of the detector PSF increases with signal strength, known as the brighter-fatter effect, we observe a variation of the smoothing that is accounted for by $\beta _{BF}$. As convolutional effects are mitigated by binning, both $\beta$ and $\beta _{BF}$ are functions of the binning values in the horizontal *H* and vertical directions *V*. The expectation value of the signal $\hat{S}_{\Omega }$ convolved with the detector PSF $\Omega$ in counts *c* on the position $\left( i,j\right)$, is summed through binning. To recover the original Poisson distribution $\mathscr {P}$ of the signal, it is rescaled by the smoothed gain. Furthermore, we identify three Gaussian distributed $\mathscr {N}$ noise contributions: 1. The variation of the signal due to the uncertainty of the gain reference $k_{H,V}$ which is influenced by the binning values and the signal distribution factor $\alpha _{H,V}$, given by Eq. 74. 2. The variation of the Poisson noise resulting from the uncertainty of the gain reference. 3. The detector noise, characterized by the variance $\sigma _{d}$, which is modified by the application of the gain reference and gain-linearization. A factor of 2 arises from the subtraction of a background frame.

In general, EFTEM does not act like an additional convolution on the image and the noise. As the signal is splitted into the respective electron-energy loss energies for EFTEM, we discard most of the incident electrons, changing the effective beam current and thus the beam correlation probability *p* (see Eq. 43). Without further changes to the noise model, the smoothing factor $\beta$ would need a reevaluation. In contrast, STEM-EELS measurements are convolved with the characteristics of the energy distribution of the beam, as we will discuss later. Note that depending on how one looks at the noise, either with respect to the noise within the image or between consecutive images, the correlation smoothing factor $\beta _{corr}$ (see Eq. 22) must be regarded or discarded as a component of the smoothing factor $\beta = \beta _{conv}\cdot \beta _{corr}$. In general, we can state that the overall uncertainty of the measurement is given by not considering the smoothing factor for the data correlation $\beta _{corr}$, whereas considering it describes the volatility of the noise of an image. This is of special interest for a series of consecutive EFTEM images or for EELS mapping.

## Evaluation of the noise model

To validate the proposed noise model, we will demonstrate how the detector and signal noises behave under various operations and provide the methodology for measuring the required parameters and factors. For clarity, we have divided the following section into three main parts: the detector noises, the noises connected to the signal, and measuring the detector PSF.

In “Section Detector noise”, we describe a method for removing cosmic rays and other artificial spike signals from the data. This is followed by an analysis of the various components of detector noise, including their time dependency and correlations, and how post-processing operations, such as applying the gain reference, affect these contributions. Additionally, we examine the effects of binning on the detector noises in “Section Binning of detector noises”.

In “Section Signal and fixed-pattern noise”, we present a detailed analysis of the noises connected to the signal. This includes the acquisition of the gain reference and the fixed-pattern noises arising from the uncertainty associated with its measurement in “Section The fixed-pattern noise of the gain reference”. Additionally, we provide an in-depth examination of the brighter-fatter effect in “Section The brighter-fatter effect”, including a method for determining its impact on measurements. We also investigate the non-linearity effect of the detector gain with increasing signal strength in “Section Correction of gain non-linearities”, highlighting the importance of accounting for the brighter-fatter effect in order to achieve a proper correction. Furthermore, we examine the effects of binning on signal noise and the uncertainty of the gain reference in “Section Binning of signal and fixed-pattern noises”.

Finally, in “Section Beam correlations and the reconstruction of the detector PSF”, we describe a method for determining the detector point spread function using the signal noise, providing a comprehensive understanding of the detector’s behavior.

### Detector noise

**Fig. 6:**
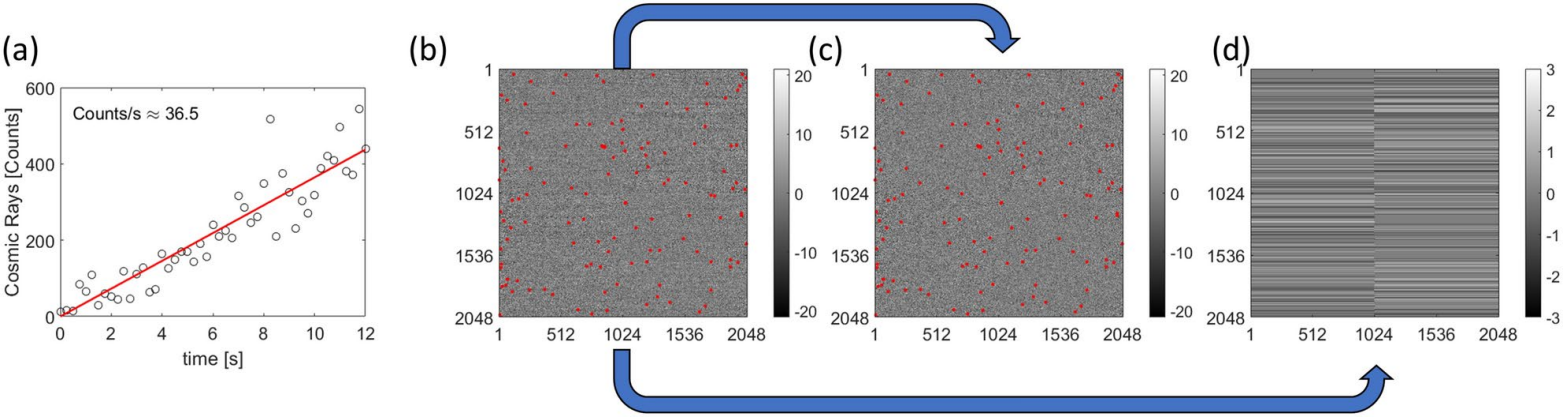
(**a**) The graph shows the cosmic ray count to increase linearly over time at a rate of approximately 36.5 counts/s. Cosmic rays are found as events that are outside by a 5$\sigma$ distance to the mean value of the image and are excluded from the noise statistics. In (**b**) an exemplary dark frame subtracted image $\xi _{DS}$ with an acquisition time of $t_{acq} = 4.75\text { s}$ is shown with the cosmic rays removed (marked as red patches). To make them visible to the eye, the dots are exaggerated as 10 times 10 pixel patches. The image was used as acquired by the *Gatan* software. By calculating the mean value of each quadrants detector rows, the image can then be separated into fast changing (**c**) read-out $\sigma _{read}$ and thermal noise $\sigma _{therm}$ as well as the slow changing (**d**) row noise $\sigma _{row,j}$ occurring as row artifacts. The images are displayed as a $5\sigma$ range around their mean.

**Fig. 7:**
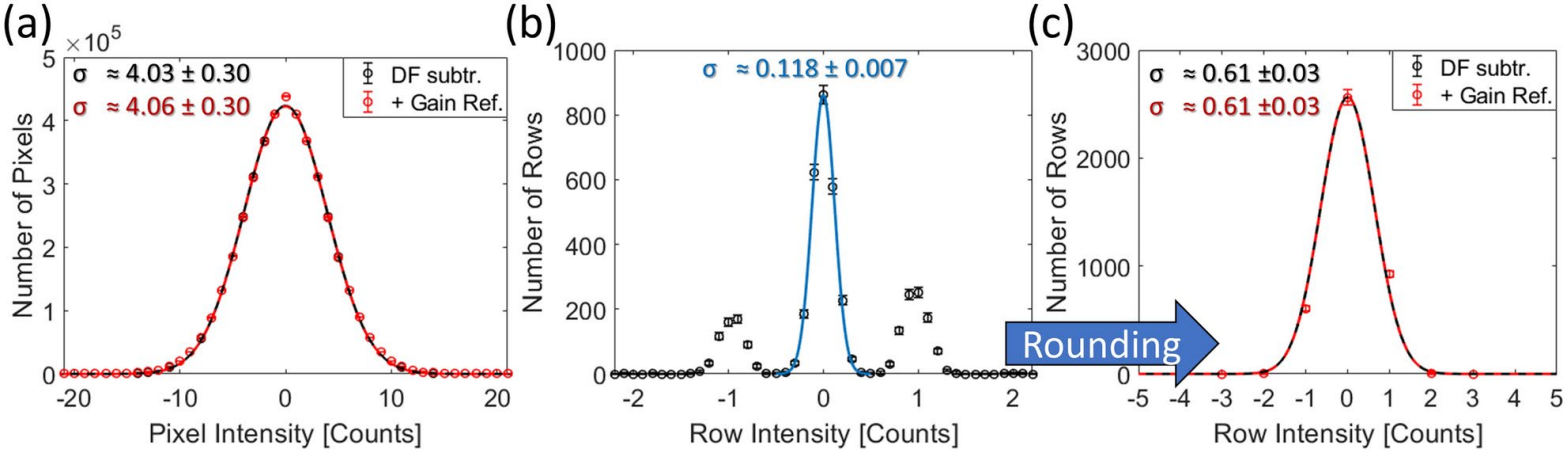
Analysis of a dark frame subtracted image with zero exposure time $t_{acq}=0\text { s}$ and with cosmic rays removed. (**a**) Distribution of the read-out noise $\sigma _{read}$ of the dark frame subtracted image after subtraction of the rounded row artifacts, with a Gaussian fit (black) superimposed. The same image with the gain reference applied (red), exhibits a slightly broadened distribution with the standard deviation changing from $\sigma \approx 4.03$ to $\sigma \approx 4.06$ counts. The error bars for both graphs show the deviations expected by the Bernoulli distributed counts and the chance of calculating a wrong mean value for a row in the row noise. (**b**) Distribution of the row noise, with a Gaussian fit (blue) superimposing the central peak. The markers indicate values within an interval of 0.1 counts around their center. The standard deviation of the Gaussian peak $\sigma _{peak} = 0.118 \pm 0.007$ counts is within the 95$\%$ confidence interval of the standard deviation of the mean $\sigma _{mean} = 0.126 \pm 0.01$ counts of the individual rows. This means that the deviations from the integers can be fully attributed to the uncertainty of measuring the mean value of the rows under the given read-out noise $\sigma _{read}$. (**c**) Distribution of the rounded row noise $\sigma _{row,j}$ of the dark frame subtracted image (black) and the same image with the gain reference applied (red). Here, the markers represent an interval of 1 around their center. The distribution can be approximated by a Gaussian distribution with a standard deviation of $\sigma = 0.61\pm 0.03$ counts for both cases. The application of the gain reference has basically no effect on the row noise.

For the evaluation of the different detector noises, images were acquired with zero emission and thus with no signal measured on the detector. Since unprocessed images do inherit a certain structure (see Fig. 4a), the following argumentation builds upon dark frame subtracted images that are much more homogeneous. However, it has to be considered that subtracting two images doubles the variance. All images were cleared of external counting events such as cosmic rays that accumulate over time (see Fig. 6a). These events appear as spikes in the image and can be detected as pixels leaving a 5$\sigma$ distance around the mean value of the image (see Fig. 6b). Since a subtracted dark frame can also contain cosmic rays, the distance is applied to both sides of the mean. The images were further separated into fractions of read-out noise $\sigma _{read}$ and thermal noise $\sigma _{therm}$ (see Fig. 6c) as well as row noise $\sigma _{row,j}$ that occurs as row artifacts (see Fig. 6d). As the thermal noise is influenced by the temperature of the detector, we cooled it to $T_{Detector}\approx -20^{\circ }$ C. By calculating the mean value of the respective row, it can be observed that the row artifacts seem to be Gaussian distributed around integers (see Fig. 7b). Considering the standard deviation of the mean, the Gaussian can fully be described by the read-out noise $\sigma _{read}$ (see Fig. 7a). Thus, it it useful to round the row artifacts to integer values (see Fig. 7c). By rounding the row mean values to integers, the error of measuring the wrong mean value can be squeezed to the order $\sigma _{mean}\approx 4 \cdot 10^{-5}$ counts, such that it is highly unlikely and statistically occurs only once every few images.

With these separate images, the noise distributions of both can be evaluated. In Fig. 7a, the overall count distribution of the read-out noise is shown to be approximated by a Gaussian. Since the image was taken with zero exposure time, it is to be expected that the thermal noise is negligible. By applying the gain reference, the noise distribution is slightly broadened (see red and black curve in Fig. 7a) according to theory (see Eq. 61). The row noise distribution in Fig. 7c, however, remains unchanged due to the rounding and the quite narrow distribution. It is also in good agreement to a Gaussian distribution. We would like to emphasize that, while being totally insignificant for regular images, the contribution of the row noise $\sigma _{row,j}$ to the overall detector noise becomes dominant for summations, e.g. for the total intensity of EEL spectra. We will show this later.

For the dark frame subtracted image $\xi _{DS}$, the count distribution is close to a Gaussian distribution. Applying the gain reference only leads to a slight broadening of the overall distribution.

To get a good approximation of the values for the read-out noise $\sigma _{read}$ and thermal noise $\sigma _{therm}$, both must be separated. As thermal noise increases over time, as dark currents accumulate, we can utilize linear regression on a time series of dark frame subtracted images $\xi _{DS}$. Further, to verify a change in the noise introduced by the gain reference, we applied the gain reference to the same images. Following Eq. 65, we get to the regression equation:

78 $$\begin{aligned} \sigma _{total}^{2} = \left( \phi _{ref,all}^{-2}+\sigma _{ref,all}^{2}\right) \cdot \left( 2\sigma _{read}^{2} + 2\sigma _{therm}^{2}\right) \quad \text {, with} \quad \sigma _{therm}^{2} \propto I_{dark} \cdot t \text { ,} \end{aligned}$$

where the gain reference introduces a multiplication by the mean value $\phi ^{-2}$ and the variance $\sigma _{ref}^{2}$ of the gain reference to both the read-out and thermal noises, according to Eq. 6. The mean value and the variance of the gain reference can be found in Fig. 5.

**Fig. 8:**
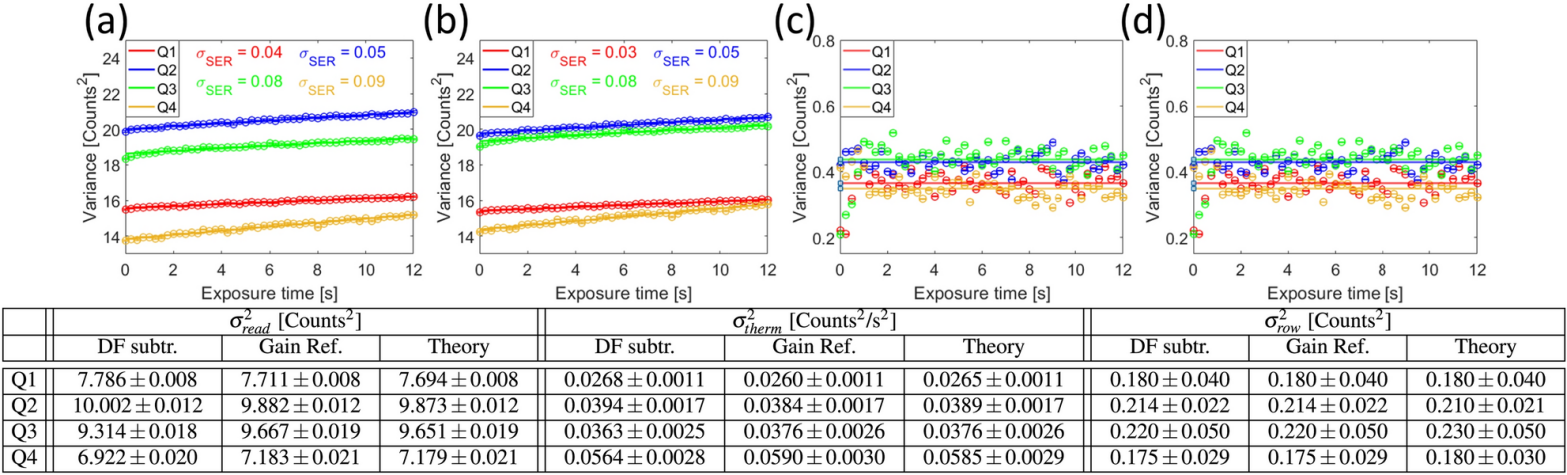
(**a**) Linear regression of the sample variance $\sigma _{total}^{2}$ over exposure time of a series of dark frame subtracted background images, according to Eq. 78, without the gain reference applied. The graphs follow the same color scheme of Fig. 1. The read-out noise $\sigma _{read}^{2}$ is given as the offset and the thermal noise $\sigma _{therm}^{2}$ as the slope of all quadrants. The uncertainties of the results are derived from the standard error of regression $\sigma _{SER}$ and a 95$\%$ confidence interval. The detector was cooled to a temperature $T_{Detector}\approx -20^{\circ }$ C. (**b**) By applying the gain reference, the values change according to Eq. 61. (**c**) The row variance can be found as the mean value of the series and (**d**) does not change with the application of the gain reference due to rounding effects. Below the graphs, the table shows the noise sample variance of the read-out noise $\sigma _{read}^{2}$, thermal noise $\sigma _{therm}^{2}$ and row noise $\sigma _{row,j}^{2}$ of the respective quadrants. These values are derived from the regression analyzes of the above graphs. For each noise, the table shows the values of the dark frame subtracted images (DF subtr.) and the same images with the gain reference (Gain Ref.) applied. We further calculated the theoretical values of the noises for the images treated with the gain reference (Theory), based on the values found in DF subtr. and the values from Fig. 5, according to Eq. 78. The values are in very good agreement, but we noted a small deviation in in the read-out noise of Q1, which is slightly outside of the measurement uncertainty.

By comparing the results of both regressions in Fig. 8a,b, it can be seen, that applying the gain reference changes the read-out noise $\sigma _{read}$, represented as the offset, and the thermal noise $\sigma _{therm}$, represented as the slope of the regression. The change in value due to the application of the gain reference can be fully explained by the $\phi ^{-1}$ and $\sigma$ values from Fig. 5 and Eq. 78. Only the value for the read-out noises $\sigma _{read}$ for Q1 deviates slightly from the theory. Nevertheless, Eq. 78 provides a good approximation. In Fig. 8c,d, the row noise is given as the mean value of the row artifacts across all images, where no time dependency is expected. Figure 8c,d might indicate some kind of different dependency for exposures below a second, which is important for EELS. Possibly due to some internal mode switching. However, the change in value due to the application of the gain reference is again according to theory (see Eq. 78).

**Fig. 9:**
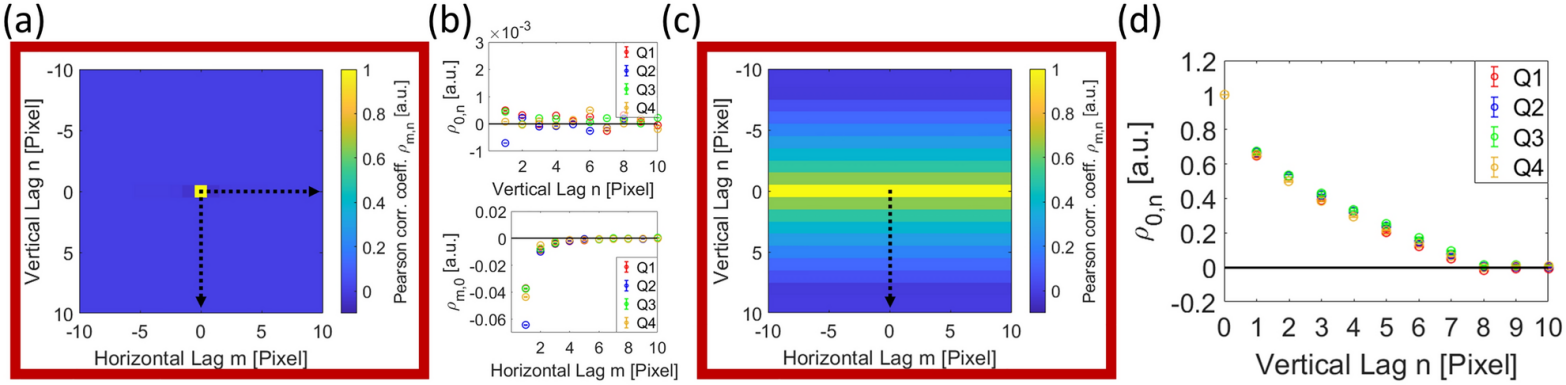
(**a**) Pearson correlation coefficients $\rho _{m,n}$ of the read-out noise $\sigma _{read}^{2}$ for the Q1 detector quadrant, indicated by the red box following the color scheme of Fig. 1. The images used for the autocovariance analyzes were cleared of the row noise (compare Fig. 6c) and averaged across 30 gain corrected background images with $t_{aqc}\approx 0.85 \text { s}$. The thermal noise $\sigma _{therm}^{2}$, which we expect to be uncorrelated, was subtracted from the central element $\sigma _{total}^{2}\cdot \rho _{0,0}$ before normalization. The black arrows indicate the position and direction in which (**b**) depicts the line profiles for all four quadrants across the respective coefficients. The values indicate a small anti-correlation in the horizontal direction, whereas the vertical profiles indicate close to no correlation effects. (**c**) Pearson correlation coefficients $\rho _{m,n}$ of the row noise $\sigma _{row,j}$ (compare Fig. 6d) for the Q1 detector quadrant, averaged across 30 background images with the same acquisition time as before. Again, the red box indicates the quadrant Q1. The black arrow indicates the position and direction in which (**d**) the line profiles for all four quadrants are shown, just like before. The coefficients indicate a strong correlation in the row noise. This phenomenon is commonly known as 1/f-noise and is typical for CCD cameras.

Eventually, an important property of the detector noise is the correlation as expressed by the Pearson correlation coefficients (see “Section Noise correlation effects”). In case of a random signal, or white Gaussian noise, the coefficients are all zero except for the central element $\rho _{0,0}=1$. For correlation, the coefficients $\rho _{m,n}$ are positive and for anti-correlation they are negative. In Fig. 9, the Pearson correlation coefficients of 30 gain corrected background images with $t_{acq}\approx 0.85\text { s}$ are averaged for all quadrants individually after the noise was separated into $\sigma _{read}$ and $\sigma _{therm}$ (compare Fig. 6c) as well as row noise $\sigma _{row,j}$ (compare Eq. 6(d)).

The autocovariance function (see Eq. 28) of the dark frame subtracted image $\xi _{DS}$ yields the Pearson coefficients multiplied by the variance $\sigma _{total}^{2}$ of the image:

79 $$\begin{aligned} K\left( \xi _{DS}\right) = \sigma _{total}^{2}\cdot \rho \!\left( \xi _{DS}\right) \text { ,} \end{aligned}$$

where we obtain $\rho _{m,n}$ as the elements of $\rho \!\left( \xi _{DS}\right)$. To further separate the read-out noise from the thermal noise (compare Eq. 78), we subtracted the values $\sigma _{therm}^{2}$ (see Fig. 8) from the central element $\sigma _{total}^{2}\cdot \rho _{0,0}$, as we expect the thermal noise to be uncorrelated and thus shaped as a Dirac delta peak $\delta$. By normalizing with respect to the central element, we obtain the Pearson correlation coefficients of the read-out noise.

The correlation coefficients for the read-out noise $\sigma _{read}$ in Fig. 9a,b indicate that there is a small anti-correlation in the horizontal row direction, whereas the noise in the vertical column direction seems to be rather uncorrelated. As the matrices derived from an autocorrelation process are necessarily Hermitian, as described in “Section Noise and convolution”, the Pearson correlation coefficients $\rho _{1,n}=\rho _{1,-n}$ and $\rho _{m,1}=\rho _{-m,1}$, which is why we only show the positive coefficients here.

In Fig. 9c,d, the correlation coefficients of the row noise $\sigma _{row,j}$ show total correlation in the horizontal row direction, which is not surprising as all the pixels have the same value (compare Fig. 7c). In vertical direction, the coefficients reveal a strong correlation, which is typically described as 1/f-noise and is often observed in CCD cameras^66^.

From the Pearson correlation coefficients in Fig. 9, we can calculate a factor $\beta _{corr}$ by employing Eq. 22, to see how correlation changes the measured variances. However, the change induced by correlation to $\sigma _{total}^{2}$ is two to three orders of magnitude lower than the uncertainty of the measurement, so it is negligible. With the values obtained from Fig. 9c, we calculate the change to $\sigma _{row,j}^{2}$ to be one order of magnitude below the uncertainty.

#### Binning of detector noises

In the following, we will show how a post-processing binning, like it is used to form EEL spectra, changes the variance of the noise. For this, we added up an increasing number of neighboring pixels to form new ‘binned pixels’, which are further analyzed. As the number of pixels is not divisible by e.g. three, we cropped the acquired images according to Fig. 1c. Since this operation changes the regarded part of the detector, we cropped the images in both directions and took the mean values of the variances.

**Fig. 10:**
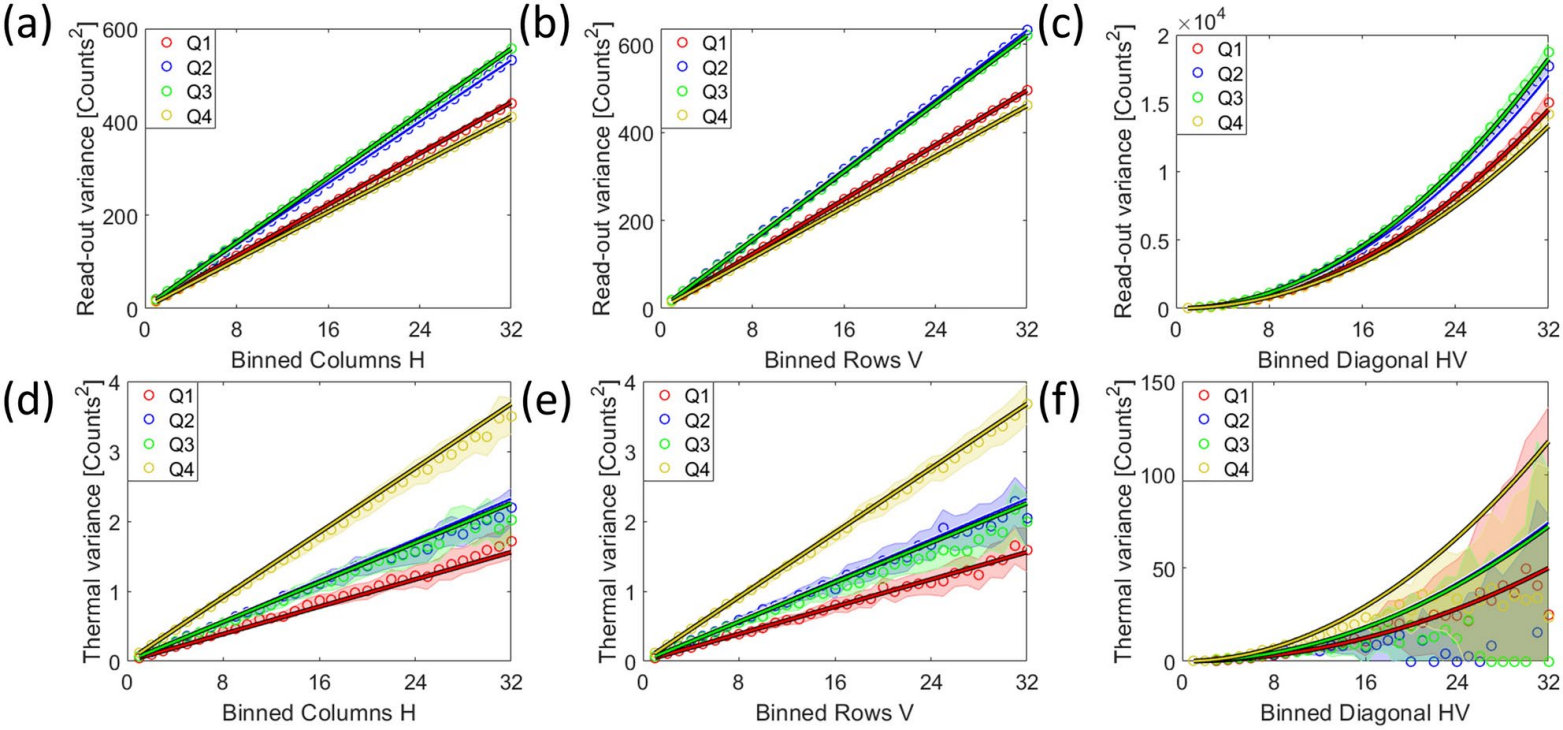
Analyzes of the read-out noise $\sigma _{read}^{2}$ and thermal noise variance $\sigma _{therm}^{2}$ of gain normalized images under post-process binning of neighboring pixels. The lines represent the reconstruction using the Pearson correlation coefficients of Fig. 9 under binning. This is achieved following Eq. 26, which yields the variance of the binned image, as well as by utilizing Eqs. 27 and 22, which allow to calculate effect on the measured variance by the correlation of the binned image. The dots represent the results of the regression analyzes similar to Fig. 8. The colors indicate the respective detector quadrant and the colored shades depict the $95\%$ confidence interval around the measured values. In (**a**), the read-out noise variances $\sigma _{read}^{2}$ of a horizontal binning process are shown in dependence of the binning value *H*. In (**b**), the vertical binning in dependence of the binning value *V* is displayed and in (**c**), the diagonal binning is shown, where rows and columns *HV* were increased equally. In (**d–f**), the summations of the thermal noise $\sigma _{therm}^{2}$ are shown using the same binning values as before. All reconstructions are in good agreement with the regression analyzes. However, the thermal noises seem to be underestimated by the regression analyzes that lead to the dots. As (**d**) and (**e)** show a linear increase of the thermal noise variance within tolerances, one would expect a quadratic increase for the binning in two directions. However, for higher diagonal binning values *HV* bigger than 10, the regression analyzes shows a discrepancy to this model.

Similar to Fig. 8, we used the regression on the same images to separate read-out noise $\sigma _{read}$ and thermal noise $\sigma _{therm}$ in a series of increasing binning values. In Fig. 10, we plot the resulting variances, where we binned *H* columns horizontally along the rows, shown in (a) and (d). We further binned *V* rows vertically along the columns, shown in Fig. 10b,e, and along a diagonal, where we binned columns and rows *HV* equally, as shown in Fig. 10d–f.

Under summation, the noise variance changes due to the covariances between the pixels, according to Fig. 25. We obtained the reconstructed noise variance from the Pearson correlation coefficients (see Fig. 9), shown as the colored lines for all quadrants in Fig. 10, to be within the uncertainty (shown as a colored shade) for the measured variances. The graphs indicate that the increase of both read-out and thermal variances grows nearly linear with the increasing number of added pixels. This is expected, as the correlation coefficients are small.

**Fig. 11:**
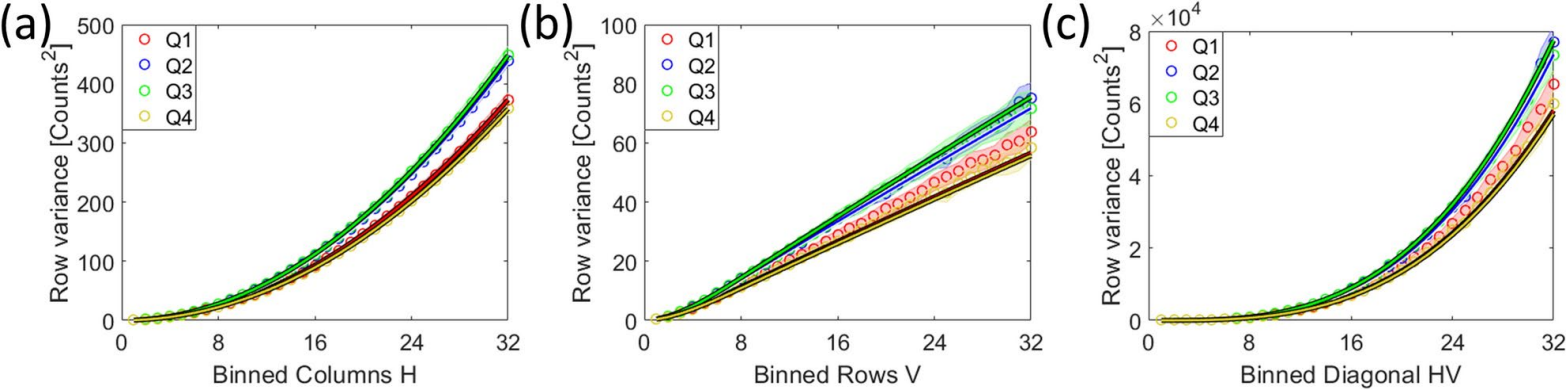
Analyzes of the row variance $\sigma _{row,j}^{2}$ of gain normalized images under post-process binning of neighboring pixels. The lines represent the reconstruction using the Pearson correlation coefficients of Fig. 9 under binning. This is achieved following Eq. 26, which yields the variance of the binned image, as well as by utilizing Eqs. 27 and 22, which allow to calculate effect on the measured variance by the correlation of the binned image. The dots represent the results of the regression analyzes similar to Fig. 8. The colors indicate the respective detector quadrant and the colored shades depict the $95\%$ confidence interval around the measured values. In (**a**), the row variance $\sigma _{row,j}$ of a horizontal binning process is shown in dependence of the binning value *H*. In (**b**), the vertical binning in dependence of the binning variable *V* and in (**c**), the diagonal binning is shown, where rows and columns were *HV* were increased equally. The Pearson reconstructions generally are in good agreement with the regression. However, the regression analysis of Q1 leading to the red dots, shows a slight deviation to higher values.

The same analysis can be conducted for the row noise $\sigma _{row,j}$ in Fig. 11. Since the mean value is identical for all pixels in a row, Fig. 11a reveals a quadratic increase of the noise variance under summation in this direction. In contrast to the read-out and thermal noises, we found a significant discrepancy between reconstruction and measured variances under binning for Q1 in Fig. 11b. Most likely, this occurs due to the high correlation of the noise and the considerably smaller sample size compared to the other noises. As the value only changes between rows, this reduces the sample size per image to a total of 1024 values per quadrant instead of 1024 times 1024 for the others. However, we think that the results of the reconstruction for the other three quadrants fit quite well.

In Fig. 11c, we obtain a cubic increase, when binning rows and columns equally. Again, we observe deviations due to the effects described in (b). One can easily see in comparison to Fig. 10 that this noise contribution becomes dominant for higher binning values. So far, we have shown that our model for the binning of detector noises in all directions is in good agreement with the theory shown in “Section Binning of the detector”.

Having shown that the detector noises behave according to theory, it is yet to be shown, how signal and fixed-pattern noises behave under different operations and how they are affected by binning in order to confirm our noise models for binning (see Eq. 77).

### Signal and fixed-pattern noise

To evaluate the signal and fixed-pattern noises, a very homogeneous signal is needed, which can be achieved in TEM-mode by spreading the beam disc at low magnification. All following signal measurements were performed at 200 kV accelerating voltage. To further reduce variations of the signals mean value, a new gain reference is acquired prior to the experiments. Afterwards, the measurements are carried out without changing the beam parameters. By employing this approach, we isolate the variations in the signal to be solely caused by Poisson and fixed-pattern noises. These noises are superimposed with the detector noises, which are thoroughly described in the previous section. We begin our investigation of the signal noises by analyzing the gain reference..

#### The fixed-pattern noise of the gain reference

For the gain reference, we acquired 30 homogeneous signal $\xi _{ref,SF}$ and dark frames $\xi _{ref,DF}$, both with the acquisition time of $t_{acq} = 0.84\text { s}$. This results in a mean target intensity of $\hat{S}_{ref,c}\approx 7050$ counts for the measured signal. The dark frames $\xi _{ref,DF}$ were recorded before any signal hit the detector to avoid delayed fluorescence or phosphorescence of the scintillation layer. The dark frames were subtracted from the signal frames:

80 $$\begin{aligned} \xi _{ref,Sig}= \xi _{ref,SF}-\xi _{ref,DF} \text { ,} \end{aligned}$$

to obtain images that are solely consisting of the signal being altered by the distribution of quantum efficiencies superimposed by noise. For further analysis, we acquired an additional 30 pairs.

From the first 30 pairs, we determine a gain reference $\xi _{ref} = \nicefrac {\xi _{ref,Sig}}{\hat{S}_{ref,c}}$ according to “Section The acquisition of a gain reference”. The mean intensity of the electrons can be converted into counts $\hat{S}_{ref,c}=g \cdot \hat{S}_{ref}$, where *g* is the gain of the detector and $\hat{S}_{ref}$ is the original signal in electrons. As described in Eq. 57, the $k_{ref}$-value describes the uncertainty of the gain reference. Subtracting two gain references, acquired with the same mean signal, therefore allows to determine the $k_{ref}$-value in dependence of the target signal strength $\hat{S}_{ref,c}$ using the standard deviation (SD) of the difference image between both. By averaging an increasing number *w* of difference images with the same target intensity, this approach allows to check Eq. 57. We choose to increase the signal strength by adding up difference images of a fixed intensity rather than increasing the exposure time to avoid non-linearities, which could alter the relation between pixels, as described in “Section Gain non-linearities”.

By increasing the number of summed frames *w*, we obtain the added pure-signal frames $\xi _{ref,Sig,w}= \sum _{w}\xi _{ref,Sig}$ and consequently a gain reference $\xi _{ref,w} = \nicefrac {\xi _{ref,Sig,w}}{\sum _{w}\hat{S}_{ref,c}}$, according to “Section The acquisition of a gain reference”. For a gain reference, the uncertainty $k_{ref}$ is determined as:

81 $$\begin{aligned} k_{ref,w} = \frac{\mathop {\textrm{SD}}\limits \left\{ \xi _{ref,w,1} -\xi _{ref,w,2}\right\} }{\sqrt{2\cdot \left( 1 + \sigma _{ref,all}^{2}\right) }} \text { ,} \end{aligned}$$

divided by $\sqrt{2}$, since the noise of both references is considered, and by the deviation of the quantum efficiencies $\sqrt{1+\sigma _{ref,all}^{2}}$, which alters the noise according to Eqs. 57 and 6. As we do not use the inverse gain reference for our analysis (compare Eq. 78), we set the mean value $\phi =1$. To get the best results, we increased the statistics by averaging the respective $k_{ref,w}$-values utilizing all other acquired images, too.

**Fig. 12:**
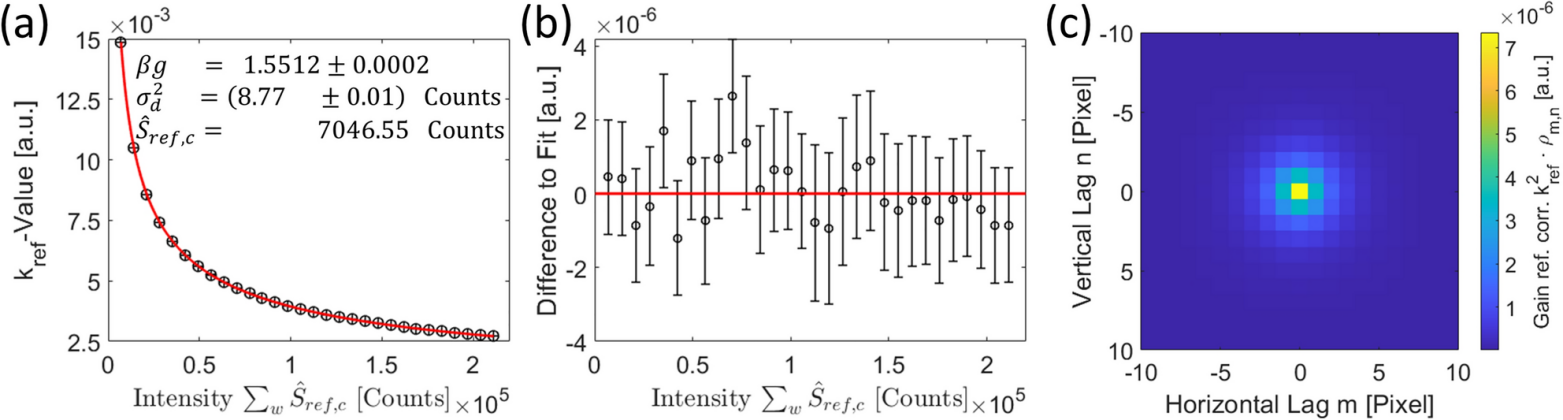
(**a**) The $k_{ref}$-value as the standard deviation of the difference of two gain references according to Eq. 81 as a function of signal strength. The signal strength was increased by summing up *w* frames with a target intensity of $g\cdot \hat{S}_{ref,c}\approx 7050$ Counts. By fitting Eq. 57 to the data, the smoothed gain $\beta \cdot g$ of the detector can be found as a parameter knowing the variance of the detector noise $\sigma _{d}^{2}$. The resulting curve is shown in red. To demonstrate the validity of the model (**b**) shows the residual plot of the difference between fit and measured data, which indicates no significant deviation. The errorbars are mainly given as the uncertainty of the standard deviation of the difference images from the signal and background frames. In (**c**) the autocovariance function resulting from Eq. 83 of the gain reference as acquired from 30 signal and background frames is shown, where the central element gives $k_{ref}^{2}$. Comparing the $k_{ref}$-values from method (**a**) $k = 0.0027111 \pm 0.000002$ with method (**c**) $k = 0.0027101 \pm 0.0000002$, according to Eq. 83 shows marginal differences most likely occurring due to the restriction of Eq. 41. To get a correct comparison between both methods, we altered (**c**) by a smoothing with Eq. 22 due to the correlation.

The uncertainty of measuring the true gain reference comprises a part that depends on the Poisson noise connected to smoothed gain $\beta \cdot g$, where *g* is the gain and $\beta$ describes the smoothing of the noise variance by convolution and correlation, according to Eqs. 37 and 22. The other part of Eq. 57 depends on the detector noise $\sigma _{d}$. In Fig. 12a the standard deviation of the difference images $k_{ref}$ is shown as a function of the summed up mean signal $\sum _{w}S_{ref,c}$ of the utilized gain references, displayed as black dots.

We obtain the detector noise as the standard deviation of the difference frames of the acquired backgrounds $\sigma _{d}^{2}=8.77 \pm 0.01$ counts. One can compare this value with a combination of the values from our previous measurement in Fig. 8, added according to Eq. 52. This results in $\sigma _{d} = 8.74 \pm 0.06$ counts, which is in good agreement not only with the previous method, but also with the value determined by the fit of the $k_{ref}$-value. By knowing the detector noise, we can rearrange Eq. 57 and obtain:

82 $$\begin{aligned} \beta \cdot g = \hat{S}_{ref,c}\cdot \left( k_{ref,w}^2 - 2\cdot \frac{\sum _{w} \sigma _{d}^{2}}{\sum _{w} \hat{S}_{ref,c}}\right) \text{ ,} \end{aligned}$$

the smoothed gain $\beta \cdot g \approx 1.5512\pm 0.0002$ as the mean value across all summed up difference pairs. With both the smoothed gain and the detector noise, we can calculate the theoretical value of $k_{ref}$ in dependence of the signal, according to Eq. 57. The resulting red curve is shown in Fig. 12a, where it is observed that the difference between the curve and the values obtained from Eq. 81 are insignificant. The difference between both are displayed in Fig. 12b as a residual plot. Considering the high precision of the experiment, we can confirm Eq. 57 as valid. By adding up 30 images for the gain reference shown in Fig. 5, we obtain a value for the uncertainty of $k_{ref} = 0.0027111 \pm 0.0000002$.

As the detector correlates the signal between pixels, we also observe the pixel values of the gain reference to be correlated. Utilizing the autocovariance function from Eq. 28 allows to determine the Pearson correlation coefficients multiplied by the variance of the gain reference $k^{2}$ in Fig. 12c.

As variances are additive, we can simply add and subtract autocovariance functions from each other and try to rebuild Eq. 57 with them. By subtracting two dark frame subtracted (pure-)signal frames $\xi _{ref,Sig}$ from each other, we obtain the difference signal image $\xi _{ref,DS}$ to inherit twice the noise variance of the signal and four times the detector noise of the dark frame (see Eq. 80). Due to the subtraction, we obtain a mean value close to zero for all pixels, which is necessary for determining the autocovariance function. Since we aim to rebuild Eq. 57, the autocovariance function must be corrected for two times the detector noise of the dark frame, which can easily be found by the autocovariance function of a difference image of two dark frames $\xi _{ref,DB}$. So, by subtracting both autocovariance functions $K\!\left( \xi _{ref,DS}\right) - K\!\left( \xi _{ref,DB}\right)$, we obtain the correct relation between signal and detector noises needed for Eq. 57. Similar to Eq. 81, we need to correct the noise for the quantum efficiency variation. To determine both, variance and Pearson coefficients of the gain reference, we repeat the process as described above, until all signal and dark frames used for the gain reference have been used for the respective autocorrelation functions and add them following:

83 $$\begin{array}{*{20}l} {k_{{ref}}^{2} \cdot \rho \left( {\xi _{{ref}} } \right) = \frac{{\sum\nolimits_{w} ( K^{ + } (\xi _{{ref,DS}} ) - K(\xi _{{ref,DB}} ))}}{{\left( {1 + \sigma _{{ref,all}}^{2} } \right) \cdot \sum\nolimits_{w} {\hat{S}_{{ref,c}} } }}} & {{\text{, with}}\qquad \xi _{{ref,DS}} = \xi _{{ref,Sig,1}} - \xi _{{ref,Sig,2}} } \\ {} & {{\;\;\text{and}}\qquad\, \xi _{{ref,DB}} = \xi _{{ref,DF,1}} - \xi _{{ref,DF,2}} \text{ ,}}\nonumber \end{array}$$

where $K^{+}$ denotes that all negative entries of the autocovariance function were set to zero, in order to correct for Eq. 41. Since the detector noise exhibits some anti-correlation effects leading to negative entries in the autocovariance function (as shown in Fig. 9), we decided not to correct these values of $K\!\left( \xi _{ref,DB}\right)$. Summing up *w* pairs, contributing to the gain reference, and dividing by the summed total intensity reveals Fig. 12c and a $k_{ref} \approx 0.00271$.

#### The brighter-fatter effect

Expanding on the idea of Eq. 83, in a next step, we try to obtain the autocovariance function of the signal itself using Eq. 64. Again, all autocovariance functions must be of zero mean for all pixels for reliable results. We can rebuild Eq. 64, if we take the autocovariance function of a dark frame subtracted and gain normalized homogeneous signal frame with its mean value subtracted $K\!\left( \xi ^{*}-\hat{S}_{c}\right)$. By doing so, we see that again we must subtract the noise variance contribution of twice the dark frame, which we obtain as the autocovariance function of the dark frame difference image $K\!\left( \xi _{ref,DB}\right)$, as defined in Eq. 83. Further, we must subtract the noise of the gain reference altering the signal. We multiply the autocovariance function of the gain reference $K\!\left( \xi _{ref}\right)$ by the mean signal $\hat{S}_{c}$. Eventually, we divide by $\left( 1+k_{ref}^{2}\right)$ to correct the signal from the alteration of the Poisson noise by the gain reference.

This procedure allows to calculate the smoothed gain and its Pearson coefficients as:

84 $$\begin{aligned} \beta _{conv}\cdot g\cdot \rho \left( \xi _{Sig}\right) = \frac{K\left( \xi _{Sig}\right) }{\hat{S}_{c}} \quad \text {, with}\quad K\left( \xi _{Sig}\right) = \frac{K^{+}\left( \xi ^{*} - \hat{S}_{c} \right) - \hat{S}_{c}\cdot K^{+}\left( \xi _{ref} \right) - K\left( \xi _{ref,DB} \right) }{\left( 1+k_{ref}^{2}\right) } \text { .} \end{aligned}$$

Again, $K^{+}$ denotes that all negative entries of the autocovariance function were set to zero. The resulting autocovariance function yields the smoothed gain $\beta \cdot g$ as its central element, shown in Eq. 84(a). For the additionally acquired signal frames, which have the same intensity as the gain reference, we obtain a smoothed gain value $\beta \cdot g \approx 1.550$, slightly smaller than predicted by Eq. 83, which was $\beta \cdot g \approx 1.551$. This difference, however, can be explained by the restriction of Eq. 41.

**Fig. 13:**
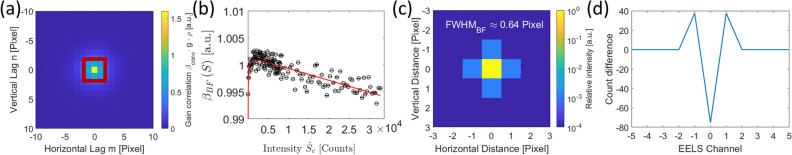
(**a**) Autocovariance function of the signal according to Eq. 84. The central value, altered by Eq. 22 due to correlation, yields $\beta \cdot g = 1.55025 \pm 0.00002$. The red box marks the nearest neighbor coefficients that were used to calculate (**b**), which depicts the relative change in the smoothing factor $\beta _{conv}$ when changing the intensity of the signal relative to the signal of the gain reference. The red curve depicts a Padé approximation of order [4/5]^67^. It can be observed that the smoothing factor decreases sharply for very low and decreases rather linearly with increasing intensities. In (**c**) the PSF of the brighter-fatter effect is shown for a spike signal of 30k counts. The PSF was reconstructed by the smoothing value of $\beta _{BF} \approx 0.995$ counts obtained in (**b**), by reversing Eq. 40 and utilizing Eq. 37. In (**d**), we show the effect under vertical summation, as it is performed e.g. in EELS. It can be seen that the effect is negligible and well below the expected Poisson noise, as only 75 counts migrate to the neighboring channels as a result of the brighter-fatter effect.

Normalizing the autocovariance function in Fig. 13a with respect to the central element yields the Pearson coefficients. Successively increasing the exposure times of the signal frame allows for a comparison of the smoothing factors $\beta$ throughout the intensity range. By following the idea of Eq. 37 that the smoothing is induced by the convolution with a detector PSF, one can easily imagine that a broader PSF leads to an increased smoothing. Adding up the Pearson coefficients, allows to determine the smoothing factor $\beta _{conv}$.

Following Astier et al.^63^, we added up the Pearson coefficients of only the neighboring pixels, marked in red in Fig. 13a, to avoid noise in the higher coefficients. We obtain the relative change of the brighter-fatter smoothing factor $\beta _{BF}$ in Fig. 13b, relative to the intensity of the gain reference, which we normalized to 1. This allows to describe the $\beta _{BF}$ factor to model the smoothing with respect to a fixed reference point, making further analysis easier to understand. For very low intensities, we observe a sharp decrease of the smoothing factor indicating an increase of the detector PSF. A reason for this behavior could be an imperfect charge transport, where a small amount of a charge carriers belonging to a given pixel is read-out into its neighbor. This leads to an increased correlation between them^63^. Further, we observe the expected brighter-fatter-effect building up for higher intensities, as charge diffusion into neighboring pixels increases^63^. We found the Padé approximation^67^, an extension of the Taylor series, of order [4/5] to fitting the signal strength dependency of $\beta _{BF}$ quite well (see red curve in Fig. 13b).

The smoothing by the brighter-fatter effect is given by a squared Gaussian kernel, resembling the PSF. So, by using the values from Fig. 13b, we can reconstruct the brighter-fatter PSF by reversing Eq. 40 and utilizing Eq. 37, which yields the smoothing coefficient as a function of the Pearson coefficients. We can estimate the brighter-fatter PSF for e.g. a 30k counts high intensity spike signal, modeled by a Dirac delta. For this specific signal strength, we obtain a slight broadening resembled by the PSF in Fig. 13c, with a standard deviation of $\sigma _{BF}\approx 0.27$ pixel or equivalently a FWHM $\approx 0.64$ pixel. Summing up the peak as in the standard procedure for EELS measurements, reveals Fig. 13d, where we show the change from to original Dirac delta peak signal to the broadened signal. We observe that for this specific signal, a total of 75 counts migrates to neighboring channels due to the brighter-fatter effect. A value that is totally negligible considering the Poisson noise connected to such a high signal and the width of a typical ZLP.

#### Correction of gain non-linearities

With all this, we can estimate the non-linearity function of the detector, following Eq. 64. The correction depends on the images before dark frame subtraction $\xi _{i,j}$ to include all offsets and is applied pixel-wise, because of large deviations in the gain distribution leading to a spectrum of different intensities within a single image. Assuming that the non-linearity function is similar for all pixels and the gain distribution is mainly altered by the fluorescence layer and the fiber optics, the goal is to determine the correction according to Eq. 67, which corrects the gain in both the image and the gain reference. As the gain is expected to change after the correction, we need to determine four fit parameters: $x_{1}$, $x_{2}$, $x_{3}$ and the smoothed gain $\beta \cdot g$.

In a series of images with homogeneous signal distribution and increasing exposure times, the total variance $\sigma _{total}$ of the images can be obtained via the sample standard deviation. It is expected to follow Eq. 70, from which we can derive a formula for the total noise variance $\sigma _{total}^{2}$ of the image, depending on the mean signal strength $\hat{S}_{c}$ in counts:

85 $$\begin{aligned} \sigma _{total}^{2} = \left( 1+k^{2}\right) \cdot \beta _{BF}\cdot \beta \cdot g \cdot \hat{S}_{c} + k^{2} \cdot \hat{S}_{c}^{2} + 2 \sigma _{d,corr}^{2} \text { .} \end{aligned}$$

where $k^2 = k_{ref}^{2} + k_{lin}^{2}$ is the uncertainty of the gain reference and the uncertainty of the linearization correction and $\beta =\beta _{conv}\cdot \beta _{corr}$ yields the smoothing factor of the measured gain $\beta \cdot g$. Further, it is important to take the brighter-fatter effect $\beta _{BF}$ into account, since we regard a large range of signal strengths. The aim of the following minimization is to find a correction function $g_{lin}\left( \xi _{i,j}\right)$ following Eq. 67 that we apply to all contributing images of the gain reference $\xi _{ref,i,j}^{corr} = g_{lin}\left( \xi _{ref,i,j}\right) \cdot \xi _{ref,i,j}$ to correct it. By further applying it to the gain normalized image $\xi _{i,j}^{Corr} = g_{lin}\left( \xi _{i,j}\right) \cdot \xi _{i,j}^{*}$ that inherits the corrected gain reference, the linearization minimizes the difference of the functional:

86 $$\begin{aligned} \mathop {\mathrm {arg\,min}}\limits _{x_{1},x_{2},x_{3},\beta \cdot g} \sum _{z}^{Z} \left( \sigma _{total,z}^{*\,2} - \left( 1+k^{*\,2}\right) \cdot \beta _{BF}\cdot \beta \cdot g^{*} \cdot \hat{S}_{c,z}^{*} - k^{*\,2} \cdot \hat{S}_{c,z}^{*\,2} - 2 \sigma _{d,corr}^{*\,2}\right) ^{2} \text { ,} \end{aligned}$$

across the entire intensity range with *Z* different intensities, in our case 160 images. We marked all variables that are changed in this minimization process with an asterisk $*$. First, the mean signal strength of the image (in counts) $\hat{S}_{c}^{*} = \frac{1}{M,N}\cdot \sum _{i,j}^{M,N} g_{lin}\left( \xi _{i,j}\right) \cdot \xi _{i,j}^{*}$ changes with the linearization $\hat{S}_{c}= g \cdot \hat{S}_{el}$, as it encompasses the gain *g* that is to be linearized. Second, changing the gain automatically changes $k_{ref}$ (see Eq. 57), as the gain is contained in the uncertainty. Third, the detector noise changes according to Eq. 69. Eventually, the sample variance of the linearization corrected image $\sigma _{total}^{*\,2} = \mathop {\textrm{VAR}}\limits \left\{ g_{lin}\left( \xi _{i,j}\right) \cdot \xi _{i,j}^{*}\right\}$ changes due to the changed noise contributions.

To further avoid correcting all signals to zero, which indeed would be the minimum of the functional, we must restrict the correction function in Eq. 67. This is achieved by normalizing the correction function in Eq. 67 by the value of the correction function at the mean signal strength of the gain reference frames $g_{lin}\left( \xi _{ref}\right)$. Therefore, all signals are corrected with respect to the actual gain at that specific reference signal.

**Fig. 14:**
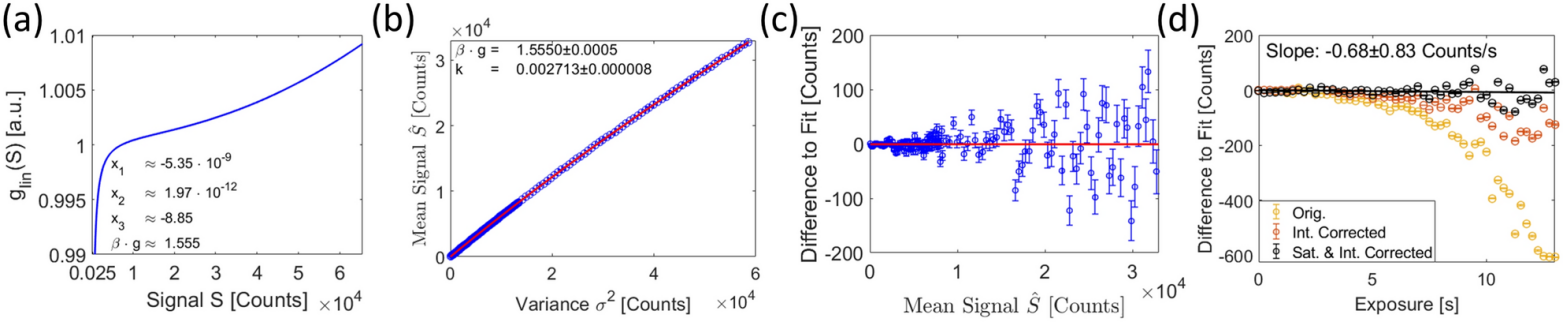
(**a**) Non-linearity correction function from Eq. 67 as a result of the minimization of Eq. 86. The fit parameters are provided within the graph. (**b**) The mean value of the corrected signal in dependency of the variance of the corrected signal and (**c**) the residual plot of the difference to the fit. (**d**) To show that the gain non-linearity correction actually works as intended, a second measurement was conducted by using the bracketed repeat-exposure (BRE) method. In contrast to (**b**), we analyzed the mean value of the image (without dark frame subtraction) as a function of the exposure time. To pronounce deviations from a linear model that we aim to archive, we used the region below 6600 counts to fit a sloped line to the respective data and subtract it. The uncorrected original data (yellow) shows a drop in value with increasing exposure time relative to a linear increase. After correcting for deviations in the beam current (orange) by the BRE-method, the drop is significantly reduced but still observable. Applying both, the non-linearity correction from (**a**) and the beam current correction, leads to the desired linear behavior, where the difference to a linear fit shows randomly distributed deviations with increasing time. A linear fit across the entire range of the data, shown as the black line, shows no significant slope within tolerances.

By iteratively choosing $x_{1}$, $x_{2}$, $x_{3}$ and $\beta \cdot g$ as fit parameters in Eq. 67, one eventually finds an optimal linearization correction for the gain, which minimizes Eq. 86 across the entire intensity range, as shown in Fig. 14a.

Using the autocovariance function on the corrected gain reference frames and on the additionally acquired signal frames of similar intensity, we obtain both the gain and k-value for the non-linearity corrected images $k \approx 0.002714$ and $g\approx 1.5544$, similar to Figs. 13a and 12c, which we can compare to the values of the regression fit in Fig. 14b. Here, we observe only small deviations. The residual plot Fig. 14c shows the differences between fit and data to be randomly distributed around the fit, showing that the applied correction really linearizes the acquired data across the entire intensity range.

However, to show that this correction actually corrects for the gain non-linearities of the system and not for some other effects, we conducted a second experiment some days apart. Again, we acquired a series of homogeneous signal frames with increasing exposure times. In contrast to the previous technique, where we utilized the noise properties of the signal in dependence on the mean value, we decided to use the mean value of the signal in dependence on the exposure time.

By employing this approach, we can completely ignore the brighter-fatter effect, as it does not alter the mean value of the signal but only the noise - carrier diffusion preserves the total amount of counts and thus the mean value. In this setup, we require the signal strength to be constant over time, such that we observe a linear relation between signal and exposure time. Deviations from the linear model can then be attributed to non-linearity effects. The problem with this technique is that the signal strength is not constant over time, but is affected by a decrease of the beam current over time.

To minimize this effect, we use a bracketed repeat-exposure (BRE) method to acquire a reference frame with a fixed exposure time of 1 s in between measurements, e.g. 0 s, 1 s, 1 s, 2 s, 1 s, 3 s, 1 s, 4 s and so on, and use the 1 s reference frames to correct for the changes in beam currents. To make deviations from a linear model easy to track, we used the region below 6600 counts to fit a sloped line to the respective data and subtract it. Thereby, we obtain the original relation as acquired (yellow) depicted in Fig. 14d, as corrected by the beam intensity decrease (orange) and additionally corrected by the non-linearity curve obtained in Fig. 14a (black). As can be seen, the correction curve linearizes the gain of this second measurement as well. We further fitted a sloped line (black) to the saturation corrected data. This slope is not significantly different from zero, which shows that the correction Fig. 14a indeed corrects for gain non-linearities and saturation effects.

We would like to note that the second experimental setup yields significantly larger deviations than the previous noise method, which we therefore consider as the better option. Again, we would like to point out that the non-linearity correction and the brighter-fatter effect are similar in magnitude. Thus, not having corrected for it in the noise-method would have lead to a significant overestimation of the non-linearity correction, which would draw visible effects in Fig. 14d. Everything fitting together so nicely is a good indication that the applied corrections are indeed valid.

#### Binning of signal and fixed-pattern noises

So far, we have shown that our model for the binning of detector noises is in good agreement with the theory shown in “Section Binning of the detector”. As a final step in verifying our noise model, we need to show how the signal and fixed-pattern noises add up under binning. Adding these noises as a result of binning primarily affects the smoothing factor $\beta \rightarrow \beta _{H,V}$, with $\beta =\beta _{conv}\cdot \beta _{corr}$, and $\beta _{BF}\rightarrow \beta _{BF,H,V}$, with $\beta _{BF}=\beta _{BF,conv}\cdot \beta _{BF,corr}$, which both change the measured gain of the detector (see Eqs. 26 and 27). Since both $\beta$ and $\beta _{BF}$ act on the gain as a smoothing factor, we combine both into a new $\beta ^{*}= \beta \cdot \beta _{BF}$. It follows that part of the convolution is removed in accordance to Eq. 37 as a consequence of binning. Therefore, the correlation of the data changes with Eq. 22.

To show the effects of detector correlation on the binning of pixels, we post-binned the data shown in the regression Fig. 14b with respect to the rows and the columns. Binning with values, which are not a divisor of 2048, leads to a remainder of pixels on one end of the detector. These pixels are discarded (see Fig. 1c). We take the average of the standard deviation of images binned both ways, front-to-end and end-to-front, in such cases. Smoothed gain $\beta ^{*}\cdot g$ and the k-value, describing both uncertainty of the gain reference and the gain linearization $k^2 = k_{ref^{*}}^2+k_{lin}^2$, are then parameters of the quadratic regression fit that can be plotted as a function of binning.

Higher exposure times, as were required for the non-linearity correction, were left out due to the increased number of cosmic rays (see Fig. 6). Replacing cosmic rays and their neighboring pixels (affected by the detector PSF) by the mean signal value of the image, causes the correlation of data to increase. Because of this, we decided to only use 1/5 of the total range of the detector, corresponding to an exposure time of around 2 s, where we expect less than 100 of such events. Further, we avoid larger uncertainties of the non-linearity correction, which might change the slope or curvature of the fit. Still, the regression analyzes contain 113 different intensities for a high precision measurement.

**Fig. 15:**
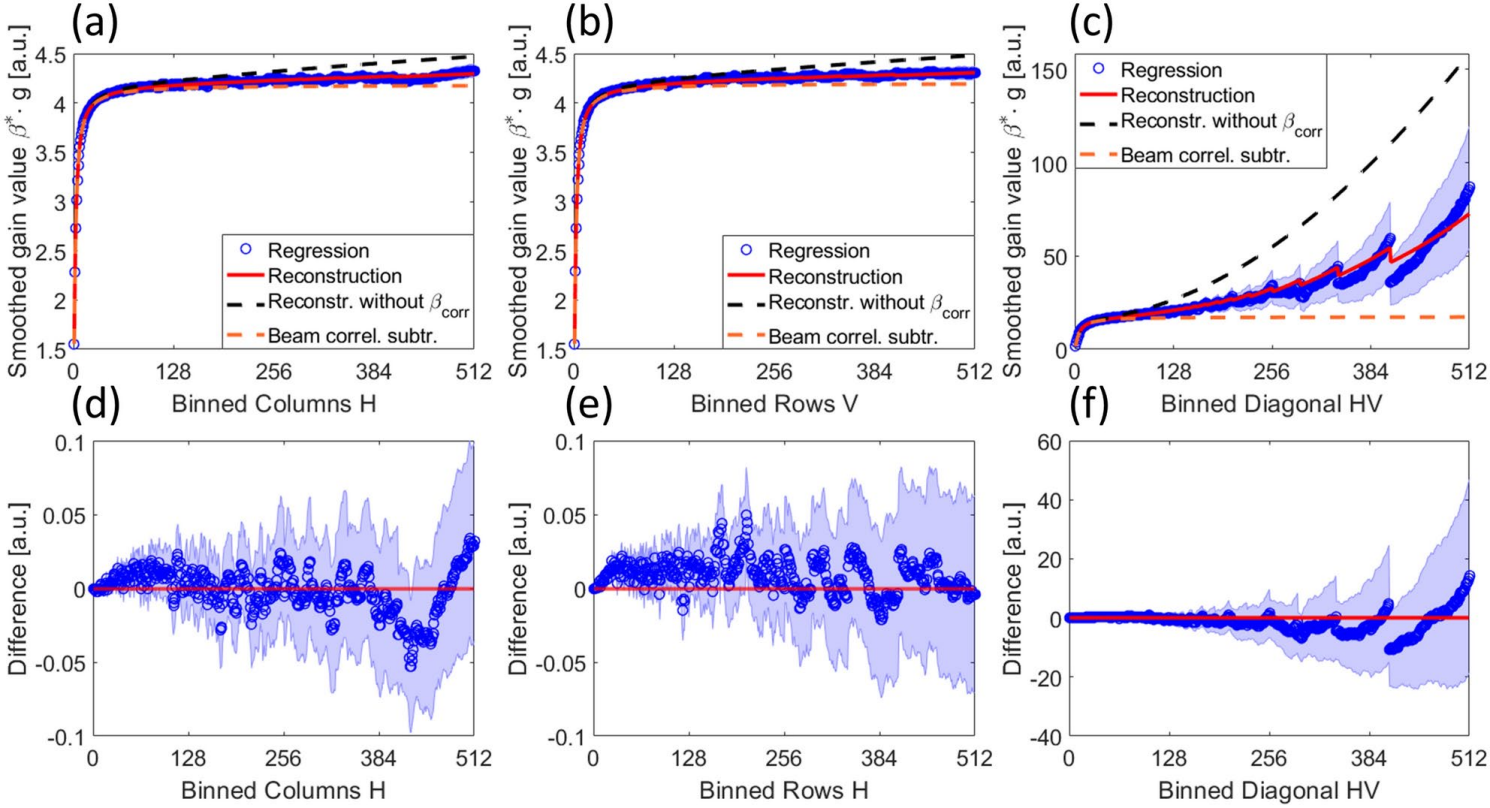
(**a–c**) The smoothed gain $\beta \cdot g$, with $\beta =\beta _{corr}\cdot \beta _{conv}$ is depicted as a function of binned pixels, as described in Eq. 72. The blue dots represent the results based on a regression analyzes, similar to Fig. 14b, but with binned pixels. The red line displays the reconstruction, based on the Pearson correlation coefficients from Fig. 13a, following Eq. 26. Since binning changes the correlation within the data, we calculate the impact of it on the smoothing $\beta _{corr}$ by Eqs. 27 and 22. The black dashed line represents the same reconstruction, but without regarding $\beta _{corr}$. The orange dashed line represents the reconstruction with the beam correlation corrected Pearson correlation coefficients. The results are shown for (**a**) horizontal binning with the binning value *H* (**b**) vertical binning with the binning value *V* and (**c**) diagonal binning along horizontal and vertical direction equally *HV*. It can be seen that both, beam correlation and $\beta _{corr}$, are necessary to find a good fit to the regression data. In the lower row (**d–f**), we provide the difference between both, the regression and the reconstruction methods. The shades depict the uncertainty within a $95\%$ confidence interval, with again the red line representing the same reconstruction as in the upper row. It can be seen that the reconstructions via Pearson coefficients are in good agreement with the regressions.

**Fig. 16:**
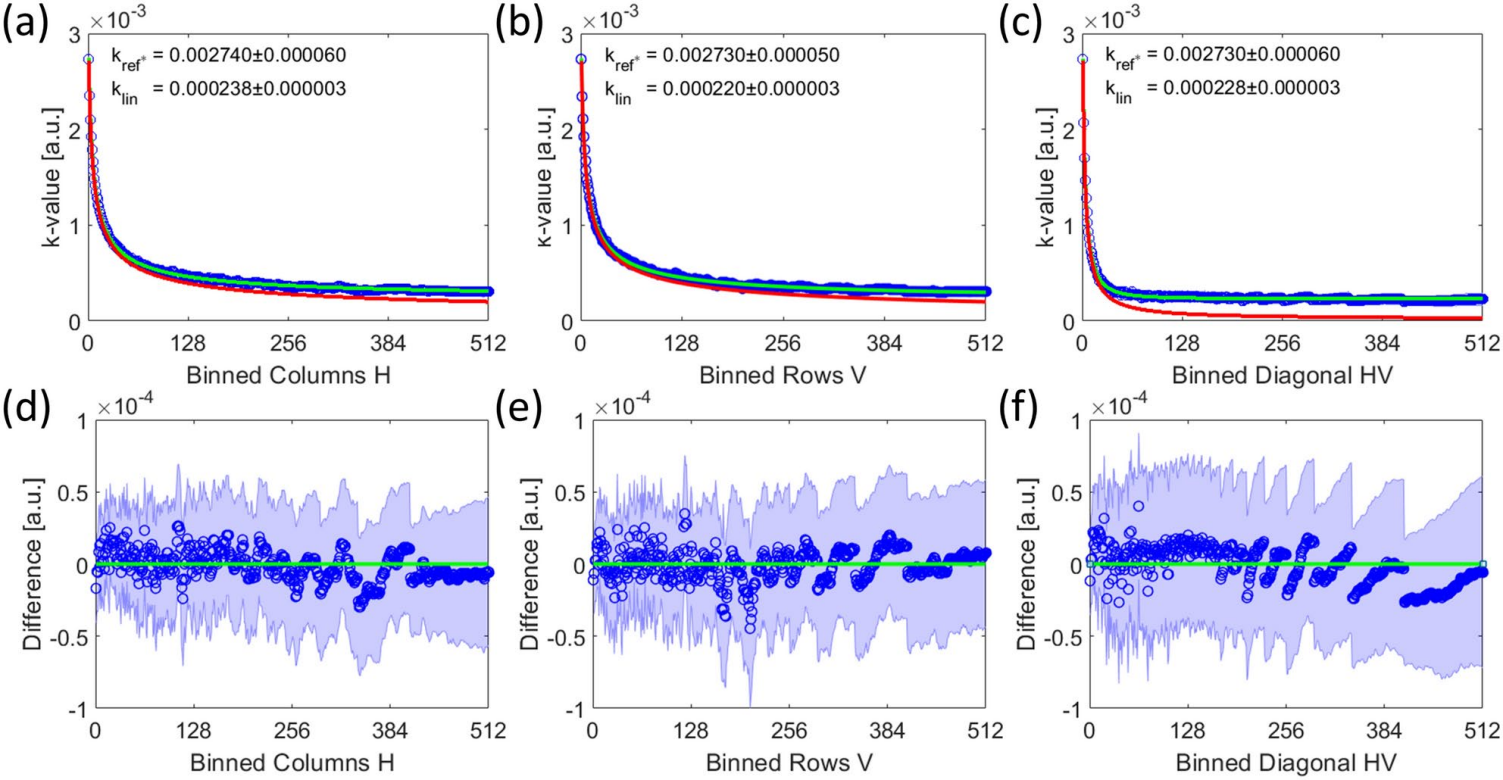
(**a–c**) The *k*-value is displayed as a function of binned pixels, as shown in Eq. 73. Here, the distribution factor $\alpha =1$, as the signal is homogeneously distributed across the detector. The blue dots represent the results based on a regression analyzes, similar to Fig. 14b, but with binned pixels. The red line displays the reconstruction based on the Pearson correlation coefficients from Fig. 12c, following Eqs. 26, 27 and 22. The uncertainty of the non-linearity correction $k_{lin}^{2}$ is independent of the binning (see Eq. 73). It is obtained by fitting an offset to the reconstructed uncertainty of the gain reference under binning $k_{H,V}^{2} = k_{ref^{*},H,V}^{2} + k_{lin}^{2}$. Combining both uncertainties under a square root yields the green line. The results are shown for (**a**) horizontal binning with the binning value *H* (**b**) vertical binning with the binning value *H* and (**c**) diagonal binning along horizontal and vertical direction equally *HV*. In the lower row (**d–f**), we provide the difference between both methods. The shades depict the uncertainty within a $95\%$ confidence interval. It can be seen that the reconstructions via Pearson coefficients plus the $k_{lin}$ uncertainty are in good agreement with the regression analyzes.

In Figs. 15a–d and 16a–c, we show the resulting gains and k-values of the regression fits as a function of the binning value in horizontal *H*, vertical *V* and diagonal direction *HV*, where we binned in both directions simultaneously. In red, we show the reconstruction solely based on adding the Pearson correlation coefficients (as described in Eq. 27) of the autocorrelation functions, shown in Figs. 12c and 13a. Additionally, the correlation effect $\beta _{corr}^{*}$ was calculated using Eq. 22. We provide the differences between both methods in Figs. 15d–f and 16d–f in the lower rows.

The graphs in Fig. 15 demonstrate that the reconstruction is a good estimator to determine the gain under binning, as the difference between the reconstruction via Pearson coefficients does not differ significantly from the regressions. We can thus confirm the summation in Eq. 72. Especially, we would like to emphasize that in Fig. 15c, we observe a huge increase in the gain for higher diagonal binning, approaching the total intensity measured on the detector. Considering that a useful detector is expected to have a rather narrow PSF, this is a contradiction at first glance, which we will resolve in a moment. For Fig. 15a,b, the gain value seems to approach a constant value, which is expected.

Further, to show the necessity of regarding the $\beta _{corr}^{*}$ coefficient (see Eq. 22), the same reconstructions without accounting for $\beta _{corr}^{*}$ are shown as the black dashed line for (a-c) and can be seen to overestimate the gain, especially for the diagonal binning (c).

In Fig. 16, the development of the k-value is shown under binning (green curve). The signal is homogeneously distributed across the detector and thus we obtain the distribution factor $\alpha \approx 1$ (see Eq. 74), making the addition as found in Eq. 73 easier. As described in “Section Gain non-linearities”, we observe an offset of the k-values obtained from the regression, relative to the reconstruction of the autocovariance function (red curve). This offset represents the uncertainty of the gain linearization $k_{lin}$. Correcting the saturation and other non-linearities is limited to the precision of the measurement and the applicability of the model. Small deviations from the real gain non-linearities lead to a small increase in the noise, but as these deviations are not correlated between pixels, we obtain no observable change for higher binning. Theoretically, this offset should be a constant independent of the direction of binning. Here, we calculate $k_{lin}$ based on a regression fit of the difference between the coefficients found by the mean-to-variance plot and the reconstruction by the Pearson coefficients. The latter must necessarily satisfy Eq. 41, as discussed previously, but mathematically it is unclear where the deviation in the Pearson coefficients manifests and thus the values might be more compromised in one direction than the other, which would explain the small deviations. Nevertheless, the deviation is so little that it accounts for only a few variance counts on the maximum intensity of the detector (without binning). Again, as $k_{lin}$ acts on the gain linearity, it acts on the difference of the intensity of signals. Considering a maximum signal of $\sim 65$k counts, we see that across the entire dynamic range of the detector, $k_{lin}$ adds a maximum of 15 counts to the overall uncertainty of the measurement. This is well below the Poisson noise of that signal with  400 counts for the standard deviation. As images often consist of small gradients forming structures, one can easily see that $k_{lin}$ is negligible for imaging.

We can thus confirm the summation Eqs. 73 and 76, as the results would otherwise deviate significantly. In total, we have shown that the binning derived in “Section Binning of the detector”, based on the changes in the smoothing factor $\beta ^{*}$ ,are according to theory.

We believe that the above results provide evidence that there is a good agreement between the shown reconstruction based on the autocovariance functions and the regression analyzes. It can therefore be assumed that the autocovariance function resembles the ‘true’ correlations within the data.

### Beam correlations and the reconstruction of the detector PSF

**Fig. 17:**
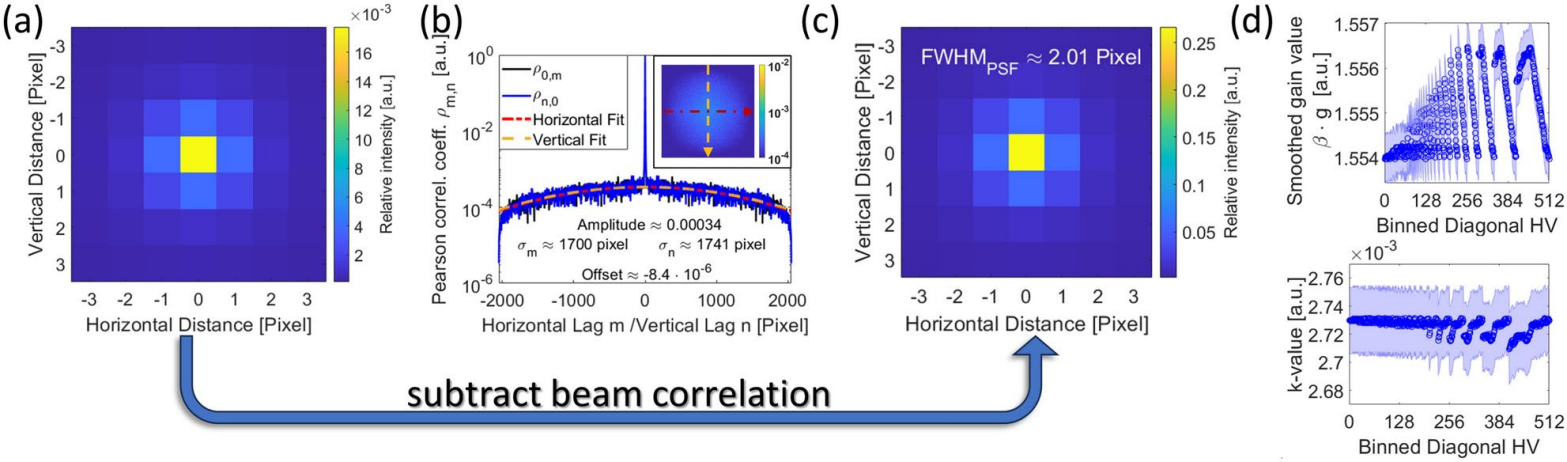
(**a**) Shows the reconstructed PSF, following Eq. 40, from the Pearson correlation coefficients depicted in Fig. 13a. The distribution implie that only 18 out of 1000 photons are detected within the intended pixel, which is unlikely given the good contrasts measured with the detector. (**b**) Shows the Pearson correlation coefficients $\rho _{m,n}$ as line profiles with $m=0$ for the horizontal profile and $n=0$ for the vertical profile. The red and orange dotted lines represent the offsetted Gaussian surface fit, with which the beam correlation is determined. The fit parameters of the surface fit are provided within the graph along with an inset, showing all the Pearson coefficients of the signal on the detector logarithmically scaled for visibility. The red and yellow lines on the inset represent the direction the respective line profiles were taken. Subtracting the beam correlation before reconstructing the PSF leads to (**c**) the detector PSF. In comparison to (**a**) the probability of photons being measured in the intended pixel is significantly increased, making it more reasonable. (**d**) Shows the change in the measured smoothed gain (above) and k-value (below), when cropping the acquired frames according to the diagonal binning Fig. 15d but without the actual binning. It can be seen that the smoothed gain increases for ‘binning values’ that discard most of the detector, contrasting Fig. 15c. Further, it can be seen that independently of the gain, the k-value decreases for the same image section.

Having determined the Pearson correlation coefficients of the signal by normalizing Eq. 84, allows to reconstruct the PSF of the detector following Eq. 40. This is feasible here, because the gain of the fluorescence layer is sufficiently larger than 1. Furthermore, it is the first layer of the detector allowing the photons to spread out with the detector PSF, as described in “Section Noise and convolution”. Typically, several hundreds to a few thousand photons are generated per incident beam electron. However, the reconstructed PSF from Fig. 17a shows a very low central peak and non-zero values in the periphery. Upon normalizing the distribution to determine the relative intensities of the PSF, we observe that only 18 out of 1000 counts are measured within the intended pixel. This is attributed to the large tails. Convolving any image with such a PSF renders it rather useless, as such a PSF diminishes any contrast. The very fact that we have contrast in the TEM is a good indication that the tails of this PSF cannot be connected to anything related to the detector itself.

Therefore, we need to take one step back and reexamine the distribution of the Pearson correlation coefficients, from which the PSF was derived. Interestingly, we observe large tails of the correlation distribution shown in (b), which means that despite the obvious small peak connected to the signal broadening as a result of diffusion, we have another low magnitude but far reaching correlation within the signal. So, the correlation must come from the electron beam itself as described in Eq. 43. Considering that effects leading to beam correlations are described and measured in^54,55^, it is highly likely that the observed long tails in the distribution of Pearson coefficients can be attributed to such effects.

In order to determine the PSF of the detector $\Omega _{d}$, the tails or offsets must be subtracted from the overall distribution of the Pearson correlation coefficients as Eq. 46 suggests. We found it suitable to approximate the beam correlation in the Pearson correlation coefficients as an offsetted 2D Gaussian $\mathscr {G}_{corr}$, which we fitted to the data shown in the inset of Fig. 17b. By omitting the region where the detector PSF dominates, we obtain the beam correlation shown as the red and the orange dashed lines in Fig. 17b. After subtracting this surface fit from the covariance function of the signal $K\!\left( \xi _{Sig}\right)$, described in Eq. 84, we obtain the detector PSF shown in Fig. 17c by following Eq. 40 as:

87 $$\begin{aligned} \Omega _{d}^{*} = \mathscr {F}^{-1}\!\left[ \left( K\left( \xi _{Sig}\right) - \mathscr {G}_{corr}\right) ^{\nicefrac {1}{2}}\right] \text { ,} \end{aligned}$$

where $\Omega _{d}^{*}$ needs to be normalized with respect to the sum of all entries to obtain the relative probabilities of the detector PSF $\Omega _{d}$. We observe that the PSF has lost the long tails, compared to Fig. 17a, and has a much more reasonable central element and a width of FWHM $\approx 2.01$ pixel, which is in the typical range for such a scintillation-based CCD detector^68^. By following Eq. 44, we can further reconstruct the probability for correlated electrons *p* from the parameters from the fit Eq. 46 and determine $p\approx 1.7\cdot 10^{-4}$. We can compare this value with the correlation found by Kodama et al.^54^, which is in the order of $p_{Kodama} \approx 2\cdot 10^{-3}$. Our electron beam exhibits correlation one order of magnitude lower, which is entirely reasonable given the different beam parameters and microscope. Thus, the correlation that we have measured falls well within the range that is already reported. The values for the width of the $\Omega _{TEM}$ distribution $\sigma _{TEM}\approx 1700$ pixel, however, are below our detector dimensions. For a homogeneous detector illumination, we would at least expect to see at least $\sigma _{TEM}\approx 4000$ pixel in width. This is a significant deviation, we cannot fully explain yet. At least Eq. 41 gives a reasonable explanation for this behavior of the autocovariance function.

For the development of the smoothed gain under binning, however, both beam and detector correlations have to be necessarily regarded. To illustrate this, we added an additional orange dashed line to Fig. 15, which shows the reconstruction according to Eqs. 27 and 22, similar to the red line, but omitting the beam correlation. Particularly in Fig. 15c, it is evident that the beam correlation is the dominant factor for the total intensity measured with the detector using higher diagonal binning. Here, the beam correlation leads to a steady increase of the smoothed gain, whereas the subtraction of these effects leads to a rather constant smoothed gain despite increasing the binning. For a detector with a small PSF, this constant behavior would be expected.

Far-reaching correlation has another notable effect on the smoothed gain $\beta ^{*} \cdot g$, which is expected when only part of the detector is analyzed. This is specifically the case for high binning values, where we need to discard larger and larger parts of the detector like in Fig. 15c. To make the effect visible, we discarded parts of the detector like in Figs. 15c and 16c, and evaluated $\beta ^{*} \cdot g$ in the non-discarded parts without binning. The results are shown in Fig. 17d, reveal the gain to steeply increase for ‘binning’ values that correspond to larger discarded parts of the detector. The reason for this behavior is that we cut out part of the correlation in the signal at the boundaries without changing the signal strength itself. From Eq. 22 follows that this must lead to a slight increase of the smoothed gain. This increase contrasts Fig. 15c at first glance, where we see the exactly opposite behavior, namely a steep decrease in the smoothed gain, where we discard large parts of the detector. Cutting away part of the correlation function drastically changes the smoothing factor to the downside, as under binning, these values now discarded would have been added up following Eq. 27. One can easily imagine that boundary effects dramatically increase for binned detectors. This explains the larger difference between the Pearson reconstruction and the regression values in Fig. 15c.

For the k-values we consider the distribution of the quantum efficiencies, according to “Section The acquisition of a gain reference”, to be more important than correlation or boundary effects, which is why we see an increase in k-value even though the gain in the upper graph drops for the same detector cutout.

**Fig. 18:**
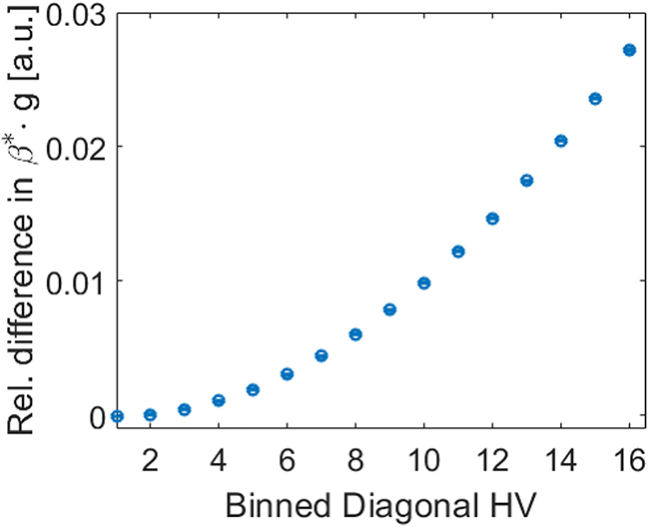
Shows the relative difference, normalized to the initial smoothed gain without binning, between the red curve of Fig. 15c, representing the binning of both the beam correlation and the detector PSF, and the orange curve, representing only the detector PSF. The relative difference shows the impact of the beam correlation on the smoothing factor under binning. Considering that diagonal binning with a factor of 2 corresponds to halving the width of the beam disc, one can see that adjusting the beam disc has a slight but measurable impact on the smoothed gain $\beta ^{*}\cdot g$. The relative difference super-linearly increases for higher binning values, which represent further contraction of the beam disc. While being rather negligible for small adjustments in the beam disc, this effect potentially plays a role for EELS measurements in STEM mode, where the beam appears as a small disc on the entrance aperture of the energy-filter.

Importantly, the beam correlation changes, when expanding or contracting the beam disc in TEM mode. The loss or gain of parts of the signal is equal to changing the beam current. With a constant correlation time, the beam correlation obviously changes. Further, by adjusting the beam, which is modeled by the deflection $\Omega _{TEM}^{*}$, the smoothing factor $\beta _{TEM,conv}$ (see Eq. 37) changes. So it is to be expected that the observed smoothed gain changes when (re-)optimizing the beam for different magnifications. This change is anticipated to be quite small. In Fig. 15c, we have shown the development of the gain with respect to binning horizontally and vertically, which with respect to the beam correlation to contracting the beam disc. Here, we must look in the difference between binning both detector PSF and beam correlation, as shown in the red curve, and the orange curve, which only gives the binning of the detector PSF. The difference should give a good approximation on how the beam correlation changes the smoothing factor $\beta$ and is shown in Fig. 18. Thus, we must abandon the notion that there is just a single ‘correct’ gain to be measured, which is universally applicable for all experiments within a certain time frame, and instead accept that only a good enough approximation is possible.

## Theoretical considerations on STEM-EELS measurements

Since the detector is also widely used for STEM-EELS measurements, we consider it important to also describe a noise model for EELS. Despite the change in operation mode, Eq. 48 remains valid for STEM with a few modifications, which we will explain in the following.

Due to a different beam path in comparison to TEM, we obtain the correlated beam from the point-source-like tip of the electron gun $S_{src,el}$ convolved with a different deflection kernel $\Omega _{STEM}^{*}$, such that $S_{STEM,el}= \Omega _{STEM}^{*}\otimes S_{src,el}^{*}$ alters the beam correlation at the entrance aperture of the energy loss spectrometer that again cuts away part of the signal. $\Omega _{STEM}^{*}$ is again normalized to the height of one. By directing the electrons through magnetic lenses, the beam disperses into the energy distribution of the probe before being projected onto the detector. We can write the dispersed STEM probe as $B_{disp}= \Omega _{disp}\otimes \Omega _{STEM}^{*}\otimes S_{src,el}$, where the two convolutions $\Omega _{disp}$ and $\Omega _{STEM}^{*}$ affect the signal like in Eq. 42: the convolutions are written inside the Poisson distribution for the uncorrelated electrons $\mathscr {P}\!\left[ \Omega _{disp}\otimes \Omega _{STEM}^{*}\otimes \hat{S}_{src,el}\right]$ and outside for the correlated electrons $\Omega _{disp}\otimes \Omega _{STEM}^{*}\otimes \mathscr {P}\!\left[ \hat{S}_{src,el}\right]$. We obtain:

88 $$\begin{aligned} B_{disp}&= p_{STEM}\cdot \left( \Omega _{disp} \otimes \Omega _{STEM}^{*} \otimes \mathscr {P}\! \left[ \hat{S}_{src,el}\right] \right) + \left( 1-p_{STEM}\right) \cdot \mathscr {P}\! \left[ \Omega _{disp} \otimes \Omega _{STEM}^{*} \otimes \hat{S}_{src,el}\right] \nonumber \\ & \approx \mathscr {P}\! \left[ \Omega _{disp} \otimes \Omega _{STEM}^{*} \otimes \hat{S}_{src,el}\right] \qquad\qquad\qquad\qquad\qquad\qquad\qquad\qquad\qquad\qquad\qquad\qquad\;\;\text { .} \end{aligned}$$

Since the signal width is expected to be rather narrow, it is unclear but likely that this cutoff changes the probability for correlated electrons $p_{STEM}$ significantly. Due to the reduced beam current flowing through the aperture, we cannot use the *p*-value from the analysis in Fig. 17b. In case that we can neglect the beam correlation, we can approximate $p_{STEM}\approx 0$. Otherwise we obtain the Pearson correlation coefficients as:

89 $$\begin{aligned} \rho _{disp,m,n}&= \left[ \left( p_{STEM}\cdot \Omega _{disp}^{*}\otimes \Omega _{STEM}^{*} + \left( 1-p_{STEM}\right) \cdot \delta \right) \star \left( p_{STEM}\cdot \Omega _{disp}^{*}\otimes \Omega _{STEM}^{*} + \left( 1-p_{STEM}\right) \cdot \delta \right) \right] _{n,m} \\ &\approx \left[ p_{STEM}^{2}\cdot \left( \Omega _{disp}^{*}\otimes \Omega _{STEM}^{*}\right) \otimes \left( \Omega _{disp}^{*}\otimes \Omega _{STEM}^{*}\right) \, + \,2\cdot p_{STEM}\cdot \left( 1-p_{STEM}\right) \cdot \Omega _{disp}^{*}\otimes \Omega _{STEM}^{*} + \left( 1-p_{STEM}\right) ^{2}\cdot \delta \right] _{n,m}\text{ ,} \nonumber \end{aligned}$$

like in Eq. 43 on the basis of Eq. 40. Under the assumption that both the distribution on the entrance aperture $\Omega _{STEM}$ and the dispersion $\Omega _{disp}$ are symmetrical, one can replace the autocorrelation $\star$ by a convolution $\otimes$ (see Eq. 30). This should be the case for $\Omega _{STEM}$. However, a symmetrical $\Omega _{disp}$ requires the ZLP to be symmetrical. Since this is often not the case, especially not for measurements with all the scattering interactions, it would be favorable to retain the autocorrelation. Still, one can argue that the ZLP is rather symmetrical and thus writing a convolution instead of autocorrelation is possible. However, this is rather to be seen as an approximation. Again, $\Omega _{disp}^{*}$ and $\Omega _{dist}^{*}$ denote the normalized PSFs to the height of one of the respective distributions. Since the two convolutions are way smaller than $\Omega _{TEM}^{*}$ for a broad beam disc in TEM mode, we cannot approximate the first term as a constant.

Again, we observe the convolution with the detector PSF $\Omega _{d}$ to influence both the correlated and uncorrelated part of the signal equally, such that we obtain the 2D EELS signal in electrons as $S_{EELS,el}= \Omega _{d} \otimes B_{disp}$ with the Pearson correlation coefficients $\rho _{EELS} = \Omega _{d}^{*}\otimes \Omega _{d}^{*}\otimes \rho _{disp}$.

The three convolutions of the beam $\Omega _{STEM}^{*}$, the dispersion $\Omega _{disp}$ and the detector PSF $\Omega _{d}$ can easily be measured in vacuum, as they describe the 2D representation of the ZLP of that measurement $\Omega _{ZLP}^{*}=\Omega _{d}\otimes \Omega _{disp}\otimes \Omega _{STEM}^{*}$. Thus, we obtain:

90 $$\begin{aligned} \rho _{EELS,m,n} & \approx \left[ p_{STEM}^2\cdot \left( \Omega _{ZLP}^{*}\star \Omega _{ZLP}^{*}\right) \, + \,2\cdot p_{STEM}\cdot \left( 1-p_{STEM}\right) \cdot \left( \Omega _{d}^{*}\otimes \Omega _{ZLP}^{*}\right) + \left( 1-p_{STEM}\right) ^{2}\cdot \left( \Omega _{d}^{*}\otimes \Omega _{d}^{*}\right) \right] _{n,m} \nonumber \\ & \approx \left[ \Omega _{d}^{*}\otimes \Omega _{d}^{*}\right] _{n,m}\end{aligned}\text{ ,} $$

with $\Omega _{ZLP}^{*}$ denoting the normalized distribution to the height of one and, in case the beam correlation is negligible, we can again simplify the equation.

Under considerable beam correlation, it is rather impossible to directly reconstruct the smoothing factor $\beta$ from the TEM mode for STEM-EELS. To obtain the smoothing factor $\beta$ under such conditions, a direct measurement of the Pearson correlation coefficients of such an EELS signal would be necessary. This measurement should be possible by utilizing Eq. 28, but requires the expectation value of the signal to be subtracted from each point on the CCD camera. Since the signal is inhomogeneously distributed, a good estimate on the expectation value of the signal can be found as the mean value of several measurements, if the microscope remains very stable.

Fortunately, the PSF of the detector is identical for both operation modes and can be considered valid in TEM as well as in STEM mode. Provided, the impact of the beam correlation in STEM mode is rather small, the PSF might give an estimate for the Pearson correlation coefficients using Eqs. 37 and 22. In this case, we can indeed use measurements from TEM mode to obtain the smoothed gain in STEM mode, since the smoothing is only connected to the detector PSF, which we have already determined in Eq. 40. By correcting Eq. 84 for the beam correlation, as shown in the previous section, and reducing the signal strength $\hat{S}_{c}$ by the correlated electrons, we obtain the smoothed gain for STEM mode:

91 $$\begin{aligned} g\cdot \beta _{EELS}\cdot \rho \left( \xi _{Sig}\right) = \frac{K\left( \xi _{Sig}\right) -\mathscr {G}_{corr}}{\left( 1-p\right) \cdot \hat{S}_{c}} \text { .} \end{aligned}$$

As $\beta$ is only determined by the detector PSF and the signal is fully confined on the detector, which is usually the case for EELS, Eq. 27 simplifies to the simple sum across all entries $\beta _{EELS} = \sum _{m,n}^{M,N} \rho _{EELS,m,n}$ (see Eq. 90). We can thus add up all Pearson correlation coefficients of the corrected autocovariance function of the signal, as shown in Fig. 17b, and determine $\beta \approx 0.09$, from which we can calculate $g \approx 17.43$ counts/beam electron as the gain of our detector. Hart et al. reported a similar gain of 12.6 counts/beam electron^69^ for an older model of our detector, the *US1000FTXP*, within a *Gatan GIF*.

Since the 2D EELS signal is summed up vertically along the columns with $V=260$ to form a spectrum on our detector, we need to consider the addition of the Pearson correlation coefficients Eq. 90 as shown in Eqs. 26 and 27. With it, we obtain $\beta _{EELS,1,260}= \beta _{EELS,conv,1,260}\cdot \beta _{EELS,corr,1,260} \approx 0.24$, following Eq. 37, for the smoothing by convolution and, following Eq. 22, for the smoothing by the correlation as values for our detector. We neglect the influence of the brighter-fatter effect, as it is contained in the measurement of a vacuum ZLP and alters it slightly with respect to the shape. As shown in Fig. 13d, it falls below the Poisson noise and is thus negligible for most measurements.

Implementing all this into Eq. 77, describing the post-binning of the detector, leads to the noise model:

92 $$\begin{aligned} \xi _{EELS}^{corr}&\approx g \cdot \beta _{EELS,1,V} \cdot \mathscr {P}\! \left[ \frac{\sum _{j}^{V}\hat{S}_{EELS,c,i,j}}{ g \cdot \beta _{EELS,1,V}}\right] + \mathscr {N}\!\left[ 0,\, \sigma _{EELS}^{2}\right] \qquad\qquad\qquad\qquad\qquad\qquad\qquad\qquad\quad\; \text {, with}\\ \sigma _{EELS}^{2}&= \alpha _{1,V}\cdot k_{ref^{*},1,V}^{ 2}\cdot \left( \sum _{j}^{V} \hat{S}_{EELS,c,i,j}\right) ^{2}+ k_{ref^{*},1,V}^{2}\cdot g \cdot \beta _{EELS,1,V}\cdot \sum _{j}^{V} \hat{S}_{EELS,c,i,j} + 2\sum _{j}^{V} \sigma _{EELS,corr}^{2}\text { ,} \nonumber\end{aligned}$$

where the EELS signal in counts is found as $S_{EELS,c} = g\cdot S_{EELS,el}$. Due to the vertical post-binning *V* to generate a spectrum from the 2D data for EELS, the distribution factor $\alpha _{1,V}$ can be derived from a 2D ZLP image, under the assumption that $\alpha _{1,V}$ is not (much) influenced by a specimen. Similar to Eq. 74, we obtain:

93 $$\begin{aligned} \alpha _{1,V} = \sum _{j}^{V} \left( \frac{\hat{S}_{EELS,el,i,j}}{\sum _{j}^{n} \hat{S}_{EELS,el,i,j}}\right) ^{2} \text { .}\ \end{aligned}$$

As described previously, the uncertainty of the gain linearization $k_{lin}$ can be neglected for normal measurements. So, we neglect it here and leave it with the uncertainty of the non-linearity corrected gain reference $k_{ref^{*}}$. Since the gain reference does not change between the operational modes of TEM and EELS, we can use the values from Fig. 16b for the amount of rows used for EELS and determine $k_{ref^{*},1,260}\approx 2.8\cdot 10^{-4}$.

For our detector, we observe that the EELS region is located in the middle of the four quadrants (see Fig. 1). Thus, we obtain the detector noise as the sum of the individual detector noises of Q1 and Q3 as well as Q2 and Q4, due to the positioning of the EELS region on the camera. As the noise depends on the respective quadrant and the EELS signal is spread across all of them, the respective value sets for the gain normalized and linearization corrected noises are given as $\sigma _{EELS,d}^{2} = \sum _{j}^{V}\sigma _{d,corr}^{2}$ (see Eq. 52 with Eqs. 65 and 70). We consider this our EELS noise model.

As an EELS signal in vacuum consists of a large ZLP rather than a homogeneous signal, the only way verifying this model is by comparing vacuum EEL spectra measured multiple times, which is quite complex. Energy-drifts must be corrected, intensity changes of the beam must be normalized and energy jitters alter the shape of a ZLP. All of these noise features must be regarded and mathematically be described. Due to the complexity of verifying this model with an actual EELS measurement and considering the already lengthy paper until now, we leave it at that.

EELS is also just a signal on the detector, that in theory, should obey the above considerations. We have good evidence that the proposed noise model is valid for regular TEM measurements and thus we have little reason to believe that EELS fundamentally changes things on a theoretical level. A follow-up paper addressing the noises in EELS is provided in reference^70^.

## Conclusions

In this work, the mathematical derivation of a noise model for scintillation-based CCD detectors is presented and it is shown how corrective operations like gain normalization affect it. We have shown which different kinds of noises are to be expected by measuring gain normalized images. Contributions of both detector and signal noise are separated and analyzed in detail. As an example for the detector architecture, we considered a *Gatan GIF Quantum ER* image filter employing the widespread *US1000FX-XP 2* CCD detector.

By utilizing the autocovariance function in Eqs. 28 and 84, we could determine the Pearson correlation coefficients of a flat image in dependence of increasing signal strength in TEM mode, which allowed to examine the brighter-fatter effect of the detector. Based on our noise model in Eq. 48, we have shown how the gain non-linearity function can be obtained by utilizing a signal-to-variance fit to counteract gain non-linearities in the CCD camera. To further prove the applicability of this correction, we have validated it using a bracketed-repeat-exposure (BRE) technique on a different data set, corrected by this method. Such a non-linearity correction is necessary to calibrate the detector to the highest precision. It also allows to use a single gain reference for the entire dynamic range of the detector, whereas recent gain references are only valid for a limited intensity range around the target intensity they were acquired with. This is specifically important for EEL spectra that cover a high dynamic range, with the ZLP often being a thousand times more intense than the typical energy-loss features of a specimen.

With the Pearson correlation coefficients of several flat images, used to obtain the gain reference to correct images with, we found a Gaussian distributed spatial correlation in the electron beam as an offset to the coefficients related to the PSF. This offset must be subtracted before reconstructing the detector PSF.

Again, we would like to point out that getting the Pearson correlation coefficients from the signal and the gain reference in TEM mode is mathematically not expensive and comprises just a few Fourier transformations and simple subtractions and divisions. It should in theory be feasible to get them as a side-product of the acquisition of a suitable gain reference using a homogeneous signal. In this paper, we have shown both theoretically and experimentally how to acquire them. We could further use these coefficients to reconstruct the change in gain under binning of neighboring pixels following Eqs. 27, 37 and 22, which is a very good indication that these coefficients are valid. With a few more steps, one could calculate the detector PSF as an automated process.

Further, we have shown that the beam correlation must necessarily change when contracting or broadening the beam disc and with it, the smoothing factor $\beta$ to the gain changes. Beam correlation contributes to the overall smoothed gain of a measurement and cannot be neglected for detector binning. This beam correlation has broader implications for STEM-EELS measurements.

Considering that beam correlation is not negligible in STEM-EELS, we concluded that it is rather impossible to reliably determine the smoothing factor $\beta$ of a STEM measurement, e.g. for STEM-EELS, by only using a TEM measurement, without further knowledge on the change of the beam correlation probability *p* between TEM and STEM. This probability naturally changes due to different beam paths and apertures. Nevertheless, we could find a noise model for EELS measurements as a special case for the regular CCD measurement, where only the beam correlation probability *p* remains as an unknown. This probability would be necessary to obtain the Pearson correlation coefficients for EELS. Therefore, we described an experimental technique to determine the Pearson correlation coefficients for EELS.

However, the point spread function and the uncertainty of the gain reference remain valid for both modes. In case that the beam correlation is indeed negligible in STEM-EELS, due to reduced beam currents, correlation effects are only determined by the detector point spread function and can be genuinely reconstructed using measurements in TEM mode, as shown in “Section Theoretical considerations on STEM-EELS measurements”.

All in all we found a consistent noise model, which allows to explain every change in the noise as a logical consequence of the previous image acquisition steps. We found mathematical descriptions and approximations of all these processes, which are mostly valid within the tolerance of our measurements and showed ways to utilize the noise to correct a CCD detector.

Noise and its statistics are powerful tools in the hands of operators, who know how to use them for their benefit. Even though it is often considered annoying to work with, we hope to have shown that sometimes it is worth to take a closer look at the noise.

## Supplementary Information

Supplementary Information.

Supplementary Information.

## Data Availability

The data sets generated during this study are available from the corresponding author upon request. Additionally, the MATLAB scripts used to analyze the noises are provided in the supplementary materials section for open access and use.
