# Supplementary material for: An applied noise model for scintillation-based CCD detectors in transmission electron microscopy
Source: Sci Rep. 2025 Jan 30;15:3815. doi: 10.1038/s41598-025-85982-4 (PMC11782531; doi:10.1038/s41598-025-85982-4)
Supplement: Supplementary file 1 — Supplementary Information. [file 41598_2025_85982_MOESM1_ESM.pdf]

An applied noise model for scintillation-based CCD detectors in transmission electron microscopy

Christian Zietlow[1,*] **and Jörg K. N. Lindner**[1]

[1]Nanopatterning-Nanoanalysis-Photonic Materials Group, Department of Physics, Paderborn University, Warburgerstr. 100, 33098 Paderborn
[*]christian.zietlow@upb.de

A Supplementary material

Our noise model contains a lot of (slightly) different variables. This supplementary information aims to support the readability of the paper by giving a short explanation of the respective variables.

Running indices and miscellaneous:

i,j	- index of the respective row, column.
m,n	- index of the respective horizontal, vertical lag.
M,N	- number of total pixels in horizontal, vertical direction.
H,V	- number of horizontally, vertically added pixels.
w	- added images.
p	- probability of correlated electrons in the electron beam.
p_{STEM}	- probability of correlated electrons in the electron beam for STEM-mode.
ρ_{TEM}	- Pearson correlation coefficient of the electron beam in TEM mode.
ρ	- Pearson correlation coefficient of the complete image formation.
ρ_{disp}	- Pearson correlation coefficient of the dispersed electron beam in STEM mode.
ρ_{EELS}	- Pearson correlation coefficient of a 2D EELS signal.
$K(\xi)$	- autocovariance function of the image ξ.
$K^{+}(\xi)$	- positivity constrained autocovariance function of the image ξ.
x_1, x_2, x_3	- fit parameters for the gain linearization correction.
Z	- used images for the non-linearity functional.
$\mathscr{G}_{corr}$	- offsetted Gaussian fit for the beam correlation in the Pearson correlation coefficients of the signal.

Gains and gain distributions:

g_{fl}	- gain of the fluorescence layer.
g_{opt}	- gain of the fiber optics layer.
g_{CCD}	- gain of the CCD detector.
g_c	- conversion gain from charge carriers in the CCD to counts.
g_d	- mean gain of the total detector including the gains g_{fl}, g_{opt} and g_{CCD}.
G_d	- gain of the detector including the variations from pixel to pixel.
$\mathscr{X}[g]$	- distribution of the quantum efficiencies from pixel to pixel.
g	- mean gain of the total detector g_d with the conversion gain g_c to convert charge carriers into counts.
$\mathscr{X}[\bar{g}]$	- normalized distribution of the quantum efficiencies from pixel to pixel around the mean gain g_d.
$\mathscr{X}_R$	- ration between the gain distribution at a given signal level and the signal level the gain reference was acquired with.
g_{lin}	- correction to linearize the non-linear gain throughout the whole dynamic range of the detector.
$\bar{g}_{lin}$	- mean value of the gain linearization of an image.

Noises, dark currents and uncertainties:

σ_{read} - read out noise of the CCD.
σ_{therm} - thermal noise of the CCD.
$\sigma_{row,j}$ - row noise of the CCD, where the offset value of a given row is constant over all pixels in that row.
μ_{row} - offset value of a given row.
$\hat{\mu}_{read}$ - offset value of the detector due to row noise and the bias of the image to prevent negative counts.
μ_{therm} - offset value of the detector due to dark currents.
$\Upsilon[\mu_{therm}]$ - distribution of dark currents.
I_{dark} - dark current.
t_{acq} - acquisition time.
σ_d - detector noise, inheriting σ_{read}, σ_{therm} and $\sigma_{row,j}$.
k_{ref} - uncertainty of the gain reference.
$k_{ref,w}$ - uncertainty of the gain reference with w summed up signal and background frames.
σ_{ref} - standard deviation of the gain reference.
ϕ_{ref} - mean value of the gain reference (all quadrant differ from 1).
σ_d^* - detector noise varied by the standard deviation σ_{ref} of the gain reference and its respective mean value ϕ_{ref} of each detector quadrant.
σ_{QE} - standard deviation of the quantum efficiencies of the detector.
k_{lin} - uncertainty of the gain linearization correction.
k_{ref^*} - uncertainty of the linearization corrected gain reference.
$\sigma_{d,corr}$ - detector noise varied by the the gain referenec and the gain linearization.
k - combined uncertainty of the linearization corrected gain reference and the linearization correction.
$k_{ref^*,H,V}$ - uncertainty of the linearization corrected gain reference under binning H pixels horizontally and V pixels vertically.
$\alpha_{H,V}$ - distribution factor of the signal on the binned pixels.
σ_{total} total noise standard deviation of a given image containing all noise contributions.

Signals of the electron beam:

$\hat{S}$ - expectation value of any of the following signals.
$\bar{S}$ - mean value of any of the following signals.
$S_{src,el}$ - stream of electrons from a point source or electron gun.
B_{el} - total electron beam (consisting of uncorrelated and correlated electrons).
S_{el} - total electron beam, broadened by the deflection of the electron-optical system.
S_d - total electron beam converted into charge carriers by the CCD.
S_c - total electron beam converted into counts.
$S_{\Omega,el}$ - total electron beam broadened by the detector point spread function.
S_{ref} - total electron beam used for the acquisition of the gain reference.
$S_{ref,c}$ - total electron beam used for the acquisition of the gain reference, converted into counts.
$S_{ref,c,w}$ - total electron beam used for the acquisition of the gain reference, converted into counts and with w summed up signal and background frames.
B_{disp} - dispersed total electron beam (consisting of uncorrelated and correlated electrons) in STEM mode.
$S_{EELS,el}$ - 2D EELS signal in electrons.
$S_{EELS,c}$ - 2D EELS signal converted into counts.

Signal broadening point spread functions (PSF) / convolution kernels:

Ω^* - denotes the normalization of the respective PSF to the height of one.
Ω_{TEM} - deflection acting on of all the beam electrons to form a beam disc in TEM mode, normalized to the height of one.
Ω_{fl} - PSF of the fluorescence layer of the detector.
Ω_{opt} - PSF of the fiber optics layer of the detector.
Ω_{CCD} - PSF of the CCD of the detector.
Ω_d - PSF of the complete detector, including Ω_{fl}, Ω_{opt} and Ω_{CCD}.
Ω_{STEM} - deflection acting on of all the beam electrons to form a point like signal in STEM mode.
Ω_{disp} - deflection acting on of all the beam electrons in the energy filter of the *Gatan GIF* to form a dispersed energy signal used for EELS measurements.
Ω_{ZLP} - combines both the dispersion PSF Ω_{disp} and the detector PSF Ω_d to represent the PSF leading to a ZLP in vacuum.

Smoothing factors:

$\beta_{TEM,conv}$ - smoothing factor of the beam convolution with Ω_{TEM}.
$\beta_{TEM,corr}$ - smoothing factor of the beam correlation in TEM mode.
β_{TEM} - smoothing factor including both beam smoothing factors for correlation and convolution in TEM mode.

$\beta_{fl,conv}$ - smoothing factor of the convolution of the fluorescence layer Ω_{fl}.
$\beta_{fl,corr}$ - smoothing factor of the correlation induced by the fluorescence layer.
β_{fl} - smoothing factor including both fluorescence layer smoothing factors for correlation and convolution.

$\beta_{opt,conv}$ - smoothing factor of the convolution of the fiber optics layer Ω_{opt}.
$\beta_{opt,corr}$ - smoothing factor of the correlation induced by the fiber optics layer.
β_{opt} - smoothing factor including both fiber optics layer smoothing factors for correlation and convolution.

$\beta_{CCD,conv}$ - smoothing factor of the convolution of the CCD Ω_{CCD}.
$\beta_{CCD,corr}$ - smoothing factor of the correlation induced by the CCD.
β_{CCD} - smoothing factor including both CCD smoothing factors for correlation and convolution.

β_{conv} - smoothing factor of all of the above convolutions.
β_{corr} - smoothing factor of all of the above correlations.
β - smoothing factor including both the smoothing factors for correlation and convolution of all of the above smoothing factors.
β_{BF} - smoothing by the brighter-fatter effect.
$\beta_{H,V}$ - smoothing factor after binning H pixel horizontally and V pixel vertically.
$\beta_{BF,H,V}$ - brighter-fatter smoothing factor after binning H pixel horizontally and V pixel vertically.
β^* - combines the smoothing factor β with the brighter-fatter smoothing β_{BF}.
β_{EELS} - smoothing factor of a 2D EELS signal (similar to β_{TEM}).
$\beta_{EELS,1,V}$ - smoothing factor of an EEL spectrum after binning V pixels vertically.

Images:

ξ - unprocessed image as acquired.
ξ_{dark} - dark frame image.
ξ_{DS} - dark frame subtracted image.
$\xi_{ref,SF}$ - unprocessed signal frame image of the gain reference.
$\xi_{ref,DF}$ - dark frame image of the gain reference.
$\xi_{ref,DS}$ - difference signal image of the gain reference.
$\xi_{ref,DB}$ - difference background image of the gain reference.
ξ_{ref} - gain reference.
$\xi_{ref,w}$ - gain reference with w summed up signal and background frames.
ζ_{ref} - actual representation of the noise on top of the gain reference.
ξ^* - dark frame subtracted and gain normalized image.
ξ^{corr} - dark frame subtracted, gain normalized and gain linearized image.
$\xi^{corr}_{Bin,H,V}$ - dark frame subtracted, gain normalized and gain linearized image, after binning H pixel horizontally and V pixels vertically.
ξ^{corr}_{ref} - non-linearity corrected gain reference.
ξ^{corr}_{EELS} - dark frame subtracted, gain normalized and gain linearized EEL spectrum.
